# A Systematic Study of Auxochrome Effects on Fluorescence Under Conditions of Varying Temperature, pH, or Viscosity: Photo‐induced electron transfer and Twisted Intramolecular Charge Transfer

**DOI:** 10.1002/open.70264

**Published:** 2026-07-21

**Authors:** Amina Bibi, Rebecca Puntorieri, Simon Wheeler

**Affiliations:** ^1^ Leicester School of Pharmacy The Gateway Leicester UK

**Keywords:** analytical chemistry, chemistry, electron transfer, fluorescence, intramolecular force, light emission, molecule, quenching, viscosity

## Abstract

It is well understood that the intensity of light emission from fluorescent molecules is affected by numerous factors including the activity of nonradiative decay pathways. Prominent among these are two intramolecular processes—photoinduced electron transfer (PET) and twisted intramolecular charge transfer (TICT). However, the variation of these quenching pathways with environmental parameters has never been systematically studied. We combine eight amine auxochromes with three fluorogenic cores to produce a matrix of 24 compounds and then examine their fluorescence while varying temperature, pH, or viscosity. We show that the activity of both PET and TICT is increased at higher temperatures, that PET can be blocked at low pH values while TICT is largely unaffected, and that increasing viscosity disrupts both pathways. This work will enhance our ability to design fluorescent molecules that are sensitive to key environmental parameters.

## Graphical abstract

The effects of temperature, pH, and viscosity on the fluorescence quenching processes PET and TICT are systematically studied by combining eight amine donors with each of three fluorogenic cores. Fluorescence intensity results from an interplay of structural and environmental factors.

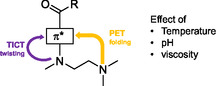

## 1 Introduction

**SCHEME 1:**
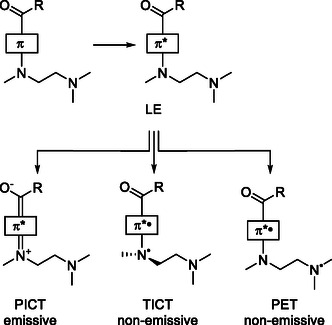
The locally excited state of fluorescent molecules can evolve into both emissive and nonemissive states

A popular class of fluorophores is D–π–A molecules where an electron donor, usually an amine, and an acceptor, usually a carbonyl, are separated by an aromatic system (Scheme 1). On irradiation they enter a state often termed locally excited (LE). Charge can be transferred, not necessarily simultaneously with LE formation [1], from the donor to the acceptor through the aromatic system causing the molecule to enter a planar intramolecular charge transfer (PICT) state. This state is emissive. It is also possible for the donor to transfer one electron to the π‐system with a simultaneous rotation around the N—C_aryl_ bond entering the twisted intramolecular charge transfer (TICT) state (first report [2], reviewed [3, 5]). The resulting diradical species is weakly emissive at most and thus the ability of the molecule to enter the TICT state reduces the quantum yield. If the molecule also contains a second unconjugated amine, e.g., pendant to the donor nitrogen, and its lone pair is in an orbital of suitable energy, then one electron can be transferred from this amine to the excited π‐system. This process is termed photoinduced electron transfer (PET) and the resulting species is nonemissive. Thus, both TICT and PET act as fluorescence quenching pathways.

A prominent theme in recent fluorophore research is to increase brightness by suppressing TICT—this has focused on modifications to the amine auxochrome. Because the twist and the charge transfer are concerted, suppressing either is sufficient to suppress the whole process. Twist suppression can be accomplished by using amine substituents that are sufficiently small to allow coplanarity with the aromatic and thus overlap of the nitrogen lone pair with the π‐system. This has been demonstrated with primary amines [6, 7] and with small cycloalkylamines [8, 10]. Charge transfer suppression can be achieved with amines containing an electron withdrawing group at either the α‐ or β‐position [11, 14], which reduces the availability of the nitrogen electrons. Some amine substituents arguably have their effect through both mechanisms [15, 16]. Also, multiple groups [6, 12, 17] have suggested that some auxochromes capable of TICT‐suppression also inhibit H‐bonding with solvent consequently reducing transfer of electronic excitation to vibrational modes [18] and contributing to increased quantum yields.

There has, however, been no systematic attempt as yet to examine how TICT‐enabled and TICT‐suppressed fluorophores may behave differently as environmental parameters are varied. To our knowledge, only a single study has reported the impact of a TICT‐inhibited auxochrome on the temperature sensitivity of fluorescence [8] and another has examined the effect on viscosity sensitivity [19]. In both cases, TICT‐suppression led to reduced environmental sensitivity compared to TICT‐enabled controls. Recent work [20] has shown that conformation, and not merely relative orbital energies, is important for the PET process—optimal PET quenching results when the donor is sufficiently close to the fluorophore. This suggests that the environment may affect fluorescence from PET‐enabled fluorophores, though this has not been systematically studied either. All these considerations are important because of the widespread use of fluorescent molecules in cell biology—the interior of lysosomes is a low pH, high viscosity environment [21, 22] while the operating temperature of mitochondria may be as high as 50 °C [23]. Building fluorescent sensors for use in these organelles therefore requires fluorophores that are insensitive to variations in these properties and will thus respond only to changes of interest (e.g., enzyme activity) and not to changes in the organellar environment.

**FIGURE 1:**
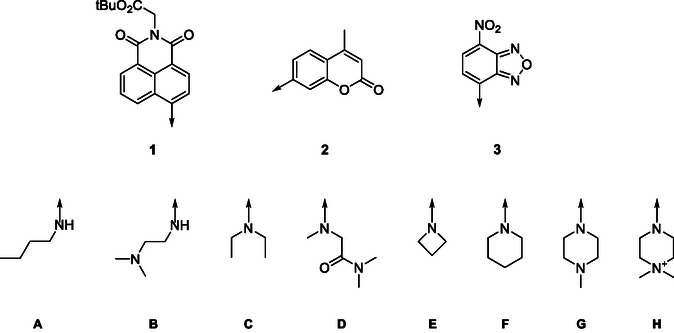
Matrix of compounds used in this study.

To supply this lack in the chemical literature, we paired naphthalimides (**1**), coumarins (**2**), and nitrobenzoxadiazoles (NBDs, **3**) with a structurally diverse array of amine auxochromes; this matrix of compounds is shown in Figure 1. We chose amines that were expected to be able to show neither TICT nor PET (**A**, **D**, **E**, **H**), TICT but not PET (**C**, **F**), PET but not TICT (**B**), and both TICT and PET (**G**). TICT‐suppression was achieved through coplanarity (**A**, **E**) or inductive withdrawal (**D**, **H**).

## 2 Results and Discussion

### 2.1 Synthesis

Naphthalimides (**1**) were prepared from the corresponding bromide [9] by displacement [24, 25], coumarins (**2**) from the requisite triflate [26] by Buchwald–Hartwig coupling [9], and NBDs (**3**) from the commercially available chloride by displacement [12]. Quaternary piperazines (**H**) were prepared from the tertiary amines (**G**) by treatment with methyl iodide. Full experimental details and characterizing data are given in the Supporting Information.

### 2.2 Photophysical Properties

Basic photophysical data were measured in acetonitrile (MeCN) as a dipolar, aprotic solvent and are summarized in Table 1. Absorption spectra, including calculation of *ε*, are presented in Figures S1–S3; emission spectra in Figure S4.

**TABLE 1:** Summary photophysical data for all compounds in MeCN.

	1				2				3			
	*λ*_abs_, nm	*ε*, M^−1^ cm^−1^	*λ*_em_, nm	*φ*	*λ*_abs_, nm	*ε*, M^−1^ cm^−1^	*λ*_em_, nm	*φ*	*λ*_abs_, nm	*ε*, M^−1^ cm^−1^	*λ*_em_, nm	*φ*
**A**	434	14,400	525	0.65	355	34,700	425	0.24	464	18,100	530	0.45
**B**	432	24,800	527	0.17	354	24,700	424	0.03	465	16,200	535	0.002
**C**	425	9100	531	0.02	368	28,200	434	0.38	486	36,500	576	0.006
**D**	422	13,500	526	0.43	358	18,600	428	0.52	475	20,900	539	0.17
**E**	444	15,000	532	0.67	357	25,100	437	0.80	485	19,100	547	0.19
**F**	409	9600	542	0.04	358	22,200	441	0.66	490	24,300	549	0.004
**G**	400	7500	531	0.03	352	21,500	436	0.05	481	19,200	555	<0.001
**H**	372	14,400	502	0.64	339	12,400	429	0.69	454	15,500	533	0.57

Patterns in quantum yield data for series **1** and **3** were broadly as expected. Thus, PET‐enabled **B** series compounds gave less intense fluorescence than their **A** analogs without this pathway; likewise TICT‐enabled auxochromes (**C**, **F**) gave fluorophores with lower fluorescence than those where TICT was suppressed (**A**, **D**, **E**, **H**). The quantum yields of **G** series compounds, where both TICT and PET can operate, were lower than those from the compounds with access to only one of these quenching pathways (**B**, **F**), indicating that both deactivation mechanisms contribute to fluorescence quenching in the same sample. In these series, the increased brightness of quaternary piperazines (**H**) relative to piperidines (**F**) must be attributed to suppressed TICT as reported [11] as neither auxochrome is PET‐enabled.

In contrast, quantum yields from **2F** and **2H** are essentially identical and there is only a small difference in quantum yield between **2C**, where TICT would be expected, and **2D**, where it would not. This is consistent with other findings [17] (see also discussion in [3]) and indicates that TICT has at most a negligible effect on fluorescence from 4‐methyl‐7‐aminocoumarins. PET is active in this series evidenced by the significantly lower quantum yields obtained with **2B** and **2G** compared with their respective controls **2A** and **2F**.

### 2.3 Effect of Temperature

We then proceeded to examine the effect of temperature on fluorescence in MeCN. Although exceptions exist (e.g., [27]), increasing temperature generally reduces the intensity of fluorescence partly due to transfer of electronic excitation to vibrational modes of the fluorophore. Turning to specific deactivation processes, the energetics of PET are described by the Rehm–Weller equation [28] which includes a term for the work done to bring donor and acceptor to sufficient proximity—this can also be seen as an energy barrier for the PET process. At higher temperatures, increased bond rotation will lead to increased formation of a conformation appropriate for PET [20], thereby enabling the molecule to surmount the energy barrier and making PET more energetically favorable. Additionally, higher temperatures will produce increased rotation of the N—C_aryl_ bond, likely leading to increased TICT. It is therefore likely that, in addition to conversion of electronic excitation to higher vibrational states, deactivation through PET and TICT will also be temperature‐dependent.

**FIGURE 2:**
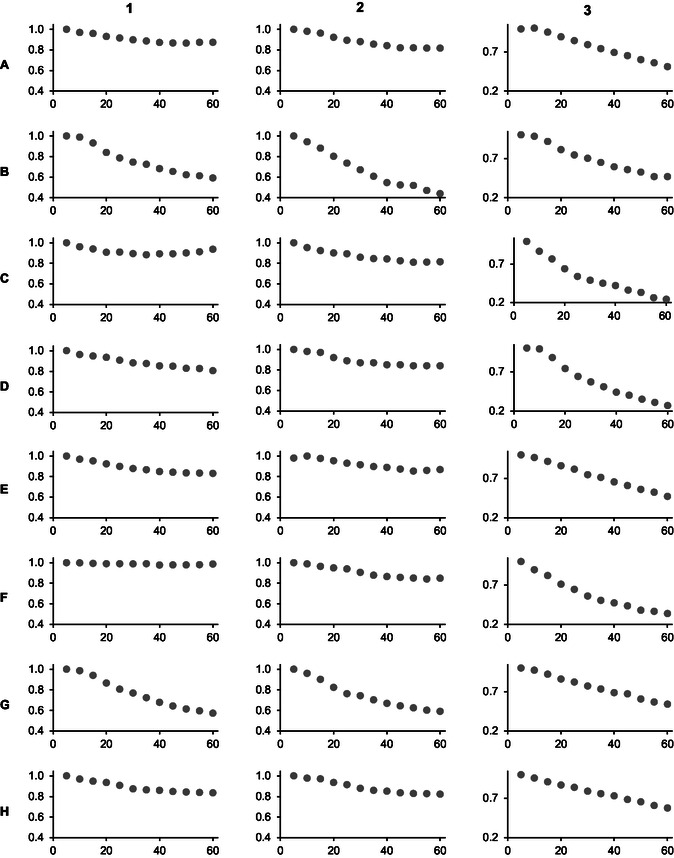
Variation of fluorescence with temperature. Temperature is plotted on the *x*‐axes and relative fluorescence at *λ*_em_ on the *y*‐axes. Note that *y*‐axes scales vary with compound series.

Consistently, we observed a significant decrease in fluorescence intensity from PET‐enabled **1B** on increasing the temperature from 5 to 60 °C in MeCN and a much smaller decrease from its control **1A** (Figure 2). To our knowledge, this is the first time that PET from an amine donor has been shown to affect the fluorescence of a molecule in a temperature‐dependent manner. There was, however, little difference in the relative decrease in fluorescence intensity between TICT‐enabled compounds **1C**, **F** and their TICT‐suppressed counterparts **1A**, **D**, **E**, **H**, indicating that, in this series, TICT does not vary significantly in the temperature range studied. The decrease in fluorescence from **1G** can therefore be attributed to increased activation of the PET channel.

The pattern in series **2** is identical to that in series **1** (Figure 2): the only significant decreases in emission intensity are from PET‐enabled **2B** and **2G**. Molecules that are in theory TICT‐enabled do not show a greater decline in fluorescence than their TICT‐suppressed counterparts (e.g., **2C** vs. **2A**, and **2F** vs. **2E**).

The NBDs (**3**) show a much more dramatic temperature‐induced fluorescence decline than the other series (Figure 2). In this context, the reduction in relative fluorescence from PET‐enabled **3B** was the same as that from control **3A**. Likewise, fluorescence from PET‐enabled **3G** does not decline more than that from its analog **3F** that lacks the possibility of PET quenching. We interpret this finding as implying that PET operates in this compound to its fullest extent even at 5 °C in MeCN and therefore increasing the temperature does not increase the degree of PET quenching. A previous computational study [20] found that formation of the polar PET state was energetically favorable for **3B** regardless of solvent polarity which similarly suggests that PET is easy for this compound. Conversely, in this series, fluorescence quenching from the TICT channel does increase with increased temperature. Thus, fluorescence from TICT‐enabled **3C** and **3F** declines slightly more than that from their respective TICT‐suppressed controls **3A** and **3E**.

In summary, PET quenching in series **1**, **2** is incomplete at 5 °C in MeCN but increases at higher temperatures likely due to conformational effects while TICT does not appreciably change in the temperature range we studied. PET quenching of series **3** is energetically easier and thus does not alter with temperature, although TICT increases slightly. This may indicate the potential of TICT‐allowed NBDs to be used as fluorescent temperature sensors.

### 2.4 Effect of pH

Turning to pH we would expect the effect of acidity to be opposite to that of temperature for PET‐enabled fluorophores: more acidic pH levels are likely to result in protonation of the distal amine group and consequent reduction in the quenching process. TICT‐inhibited fluorophores are unlikely to change emission at lower pH as the aniline nitrogen lone pair will be rendered less available by delocalization into the aromatic system and/or inductive withdrawal.

**FIGURE 3:**
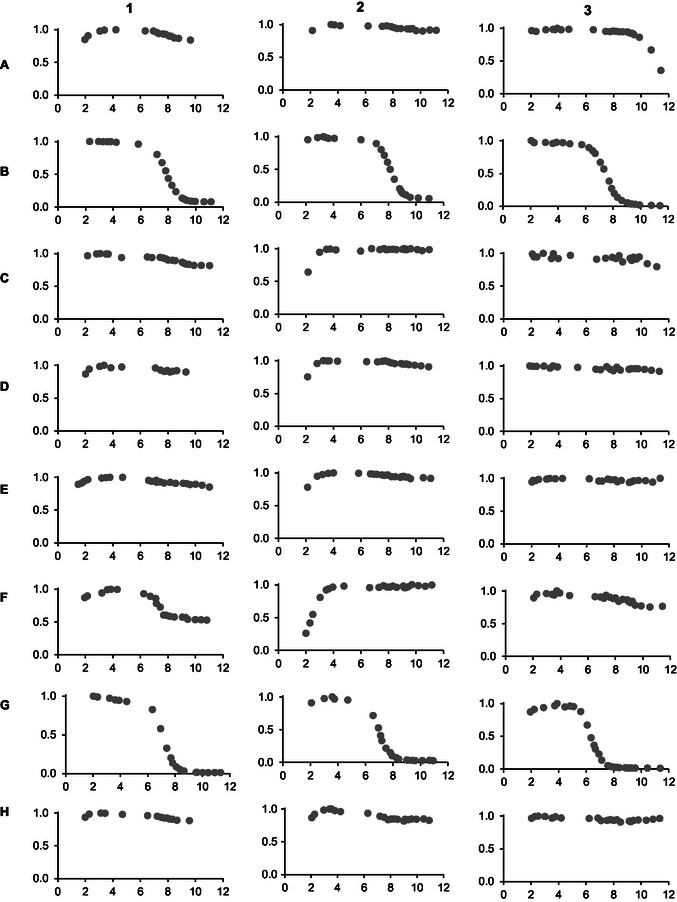
Variation of fluorescence with pH. pH is plotted on the *x*‐axes and relative fluorescence at *λ*_em_ on the *y*‐axes. Note that *y*‐axes scales vary with compound series.

The pH dependence of fluorescence from napthalimides substituted with auxochromes **B** has been studied previously [29] and showed significant increases at lower pH values in line with expectations. We sought to generalize these findings by examining fluorescence in 1:1 mixtures of MeCN/buffer at various pH values (see Supporting Information for experimental details). Consistently fluorescence from all PET‐enabled **B** series compounds increased in intensity on titrating from pH 11 to pH 2 (Figure 3) without change in spectral form (Figure S5).

Similarly, there are isolated examples of the pH dependence of fluorescence from napthalimides substituted with auxochrome **G** [30, 31]. Protonation of compounds of this series renders them isoelectronic to those of the **H** series and thus suppresses TICT as well as PET [11], resulting in significant increases in fluorescence intensity (Figure 3).

All other compounds gave approximately stable fluorescence over the pH range studied with the exceptions of **2F** at low pH, which we attribute to protonation of the piperidine nitrogen, and **3A** at high pH, which we attribute to deprotonation of the aniline nitrogen. Allowing for these exceptions there was little difference in behavior between TICT‐enabled (series **C**, **F**) and TICT‐suppressed (series **A**, **D**, **E**, **H**) compounds. This leads to the previously unreported conclusion that TICT in these D–π–A molecules is largely invariant with pH except in the special case of piperazine (series **G**).

### 2.5 Effect of Viscosity

**FIGURE 4:**
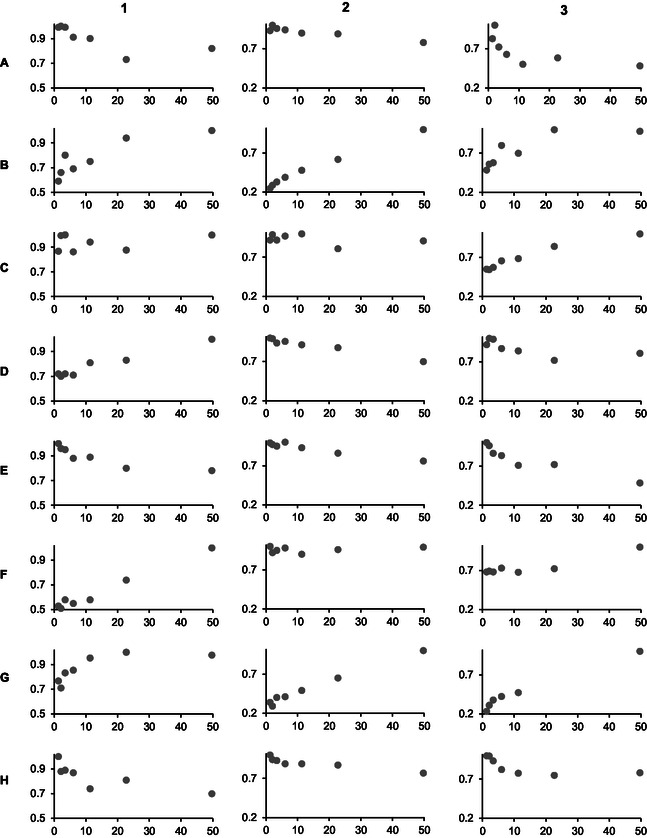
Variation of fluorescence with viscosity. Viscosity (cP) is plotted on the *x*‐axes and relative fluorescence at *λ*_em_ on the *y*‐axes. Note that *y*‐axes scales vary with compound series. See Table S2 for *λ*_ex_ and *λ*_em_ for each sample.

Finally, we turned our attention to the effect of viscosity on fluorescence. It is widely accepted that increased solvent viscosity will produce increased microfriction resulting in decreased molecular rotation therefore decreased nonradiative decay and increased emission [32, 33]. In particular, increased viscosity may inhibit the ability of a molecule to adopt a conformation where donor and acceptor are sufficiently close to allow PET and/or the rotation of an N—C_aryl_ bond to allow TICT. We examined the behavior of our compounds in the relatively narrow range of 1–50 centipoise (cP) using the standard method [34, 35] of measuring fluorescence in methanol–glycerol mixtures with well‐defined viscosities—the results are shown in Figure 4.

In the PET‐enabled **B** series, fluorescence intensity reliably increased with viscosity. This phenomenon has been reported previously in a single example [20]. Our data suggest that it is likely to be general. Increasing viscosity probably retards the folding necessary for the molecules to attain the correct conformation for PET to occur.

Compounds which are TICT‐enabled but without PET (**1F**, **3C**, **3F**) also showed increases in fluorescence at higher viscosity likely resulting from inhibition of N—C_aryl_ twisting and thus TICT suppression. (Consistent findings have been reported with an analog of **1F** [36]) **1C** is an exception to this pattern, implying that at ambient temperature it is in a twisted conformation at all viscosity values and thus unable to attain the more emissive PICT state. In support of this we found that the Stokes shift of this compound is not solvatochromic (Figure S7) unlike all other compounds in series **1,** indicating that its dipole moment does not change on excitation. Neither **2C** nor **2F** showed fluorescence intensity changes with viscosity (previously reported for **2C** [37]) consistent with negligible TICT in this series. Therefore, the fluorescence enhancement seen with **2G** results from suppression of PET while that from **1G** and **3G** can likely be attributed to suppression of a combination of both PET and TICT.

We also asked the previously unaddressed question of whether the emission intensity from fluorophores bearing TICT‐suppressed amine donors varies in a viscosity‐dependent manner. Our data show that regardless of whether TICT is suppressed by enforced coplanarity (series **A**, **E**) or inductive electron withdrawal (series **D**, **H**) increased viscosity resulted in decreased fluorescence intensity in all three series. This included the coumarins (**2**) where TICT does not appear to operate. We speculate that, just as increased viscosity inhibits the twisting of amine substituents out of plane to access the TICT state, it can also inhibit twisting into plane to access the emissive PICT state. If TICT is already suppressed by the nature of the amine substituent, then increasing viscosity inhibits only PICT formation and thereby reduces emission intensity. At the same time, we cannot exclude a contribution from H‐bond‐mediated quenching [18] given the higher hydrogen bonding capacity of glycerol than methanol.

## 3 Conclusion

This study of D–π–A fluorophores has made a number of observations that are novel to the best of our knowledge and has offered generalizations of others that were previously known only from isolated examples. First, we have shown that both PET and TICT deactivation channels can operate simultaneously in a single sample of a fluorophore. Second, fluorescence quenching of this fluorophore class through both PET and TICT channels can increase with temperature in acetonitrile, though the changes in TICT are generally much less pronounced and sometimes undetectable. Third, we have confirmed that acidic conditions that protonate a pendant nitrogen block PET and so increase fluorescence while TICT is largely unaffected by pH changes as the aniline‐type nitrogens are not basic enough to be protonated except at quite extreme values. Fourth, increasing viscosity reduces fluorescence quenching by both mechanisms; however, fifth, if TICT is already suppressed by the structure of the amine donor, then increasing viscosity leads to a decrease in fluorescence.

We hope that these observations will further our understanding of fluorophore design and so help in the ongoing work to develop fluorescent sensors where sensitivity to the parameters we have studied is demanded. Equally, given that some amine substituents are relatively unaffected by these parameters we hope that it will enable the design of chemical sensors whose output remains unchanged when temperature, pH, and viscosity vary. For example, substituent **G** has previously been used to generate sensors targeted to lysosomes [11] whose interior is in the approximate pH range 4–5.5. In this environment, **G** will become protonated making it isoelectronic with **H**. It is thus important for the construction of lysosomal probes that the fluorescence from compounds substituted with **H** does not vary with viscosity as this parameter does not appear to be constant in these organelles (e.g., [21]).

TICT is suppressed (by inductive electron withdrawal) in sarcosinamide auxochrome **D** [12], but this series sometimes shows behavior that is apparently inconsistent with this. Thus, the temperature sensitivity of **3D** is more similar to TICT‐enabled **3C** than to TICT‐suppressed **3H** (Figure 2), and fluorescence from **1D** increases with viscosity while that of all other TICT‐suppressed compounds decreases. (We examined fluorescence from this compound in water with and without 0.2% w/v carbomer and obtained equivalent results (Figure S8), thus eliminating compound‐specific solvent effects as an explanation.) The amide provides an attractive functional handle that can potentially be derivatized, e.g., with SNAP‐Tag, to localize sensors containing this amine to desired subcellular compartments. However, the slightly unpredictable behavior induced by this moiety demands that any sensors based on it would need to be thoroughly tested in vitro before being deployed in cellular contexts. Work to understand better the characteristics of this substituent is underway in our laboratories and will be reported in due course.

## Funding

This work was supported by the Royal Society of Chemistry (R24‐4710323201).

## Conflicts of Interest

The authors declare no conflicts of interest.

## Supporting information

Supplementary Material

## Data Availability

The data that support the findings of this study are available from the corresponding author upon reasonable request.
