# Supplementary material for: A Systematic Study of Auxochrome Effects on Fluorescence Under Conditions of Varying Temperature, pH, or Viscosity: Photo‐induced electron transfer and Twisted Intramolecular Charge Transfer
Source: ChemistryOpen. 2026 Jul 21;15(8):e70264. doi: 10.1002/open.70264 (PMC13388028; doi:10.1002/open.70264)
Supplement: Supplementary file 1 — Supplementary Material [file OPEN-15-e70264-s001.pdf]

Supporting Information for

The variation of fluorescence quenching pathways PET and TICT under conditions of varying temperature, pH or viscosity

Amina Bibi (0009-0005-1934-4916), Rebecca Puntorieri (0009-0006-5984-0436) and Simon Wheeler (0000-0003-0215-8648)

Leicester School of Pharmacy, The Gateway, Leicester, LE1 9BH, UK

Synthetic details	2
Photophysical experiments	15
NMR data	30
HRMS data	54
References	60

Synthesis

Chemicals were obtained from commercial suppliers and used without further purification unless otherwise stated. Dioxane was Extra Pure grade (Fisher); petroleum ether refers to the fraction boiling at 40-60 °C. NMR spectroscopy was carried out in the stated deuterated solvents using a JEOL 600 MHz spectrometer at 298 K. Chemical shifts are expressed in ppm. High resolution mass spectra were obtained using a Waters XevoG2-XS with a QTOF detector.

Preparation of series 1

1A – **1H** were prepared from intermediate **X** prepared as previously described and giving identical spectroscopic data to that reported.[1]

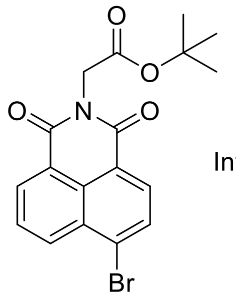

Intermediate **X**

Preparation of **1A**

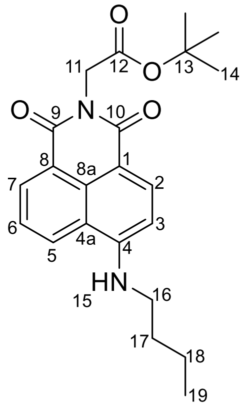

In a ReactiVial n-butylamine (1mL, xs) and intermediate **X** (0.106 g, 0.272 mmol, 1 equiv) were added and stirred overnight at 50°C, TLC showed completion of the reaction, and n-butylamine was evaporated. Crude product was purified by column chromatography over silica eluting with (15% EtOAc/Hexane). Relevant fractions were combined and evaporated to a yellow solid product (65.2 mg, 0.170 mmol, 63 %).

^{1}H NMR ($CDCl_3$, 600 MHz): 0.98 (3H, t, J= 7.22 Hz, H19), 1.47 (2H, p, H18), 1.55 (9H, s, H14), 1.70 (2H, m, H17), 3.24 (2H, t, J=7.39, H16), 4.81 (2H, s, H11), 6.37 (1H, d, J= 7.9 Hz, H3), 7.23 (1H, appears t, J= 7.7 Hz, H6), 7.97 (1H, appears d, J= 8.3 Hz, 1.7 Hz, H5), 8.13 (2H, two proton multiple, H2, H7)
^{13}C NMR ($CDCl_3$, 100 MHz):13.9 (C19), 20.5 (C18), 28.3 (C14), 30.8 (C17), 41.9 (C11), 43.7 (C16), 82.4 (C13), 104.2 (C3), 108.7 (C1), 119.9 (C4a), 121.8 (C8), 124.3 (C6), 126.8 (C5), 129.3 (C8a), 131.0 (C7), 134.4 (C2), 150.1 (C4), 163.5 (C10), 164.3 (C9), 168.8 (C12).
HRMS (ESI) *m/z* calculated for $C_{22}H_{26}N_2O_4.Na^+$ 405.1790, found 405.1772.

Preparation of **1B**

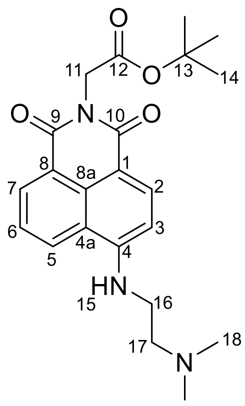

To a solution of intermediate **X** (104 mg, 0.267 mmol, 1 equiv) in 2-methoxyethanol (3 mL) was added $CuSO_4.5H_2O$ (34 mg, 0.289 mmol, 1 equiv), N, N-dimethylethylenediamine (55 μL, 0.513 mmol, 2 equiv), and Triethylamine (54 μL, 0.384 mmol, 1.5 equiv). The mixture was heated at 130°C in a ReactiVial. After 22h, TLC showed the completion of the reaction, and the mixture was poured into water (10 mL). The resulting suspension was extracted with ethyl acetate (20 mL). The combined organic layers were washed with brine, dried over $MgSO_4$, filtered, and evaporated. Crude product was purified by column chromatography over silica, eluting with ethyl acetate, then 95:5:0.5 (DCM/MeOH/880 NH_3). Relevant fractions were combined and evaporated to obtain product (19.8 mg, 0.0498 mmol, 19%) as a yellow solid.

^{1}H NMR ($CDCl_3$, 600 MHz): 1.48 (9H, s, H14), 2.38 (6H, appears bm, H18), 2.79 (2H, appears bm, H17), 3.40 (2H, appears dd, H16), 4.81 (2H, s, H11), 6.59 (1H, d, J= 8.4 Hz, H3), 7.57 (1H, appears t, J= 8.0 Hz, H6), 8.20 (1H, dd, J= 8.3 Hz, 1.7 Hz, H5), 8.40 (1H, d, J= 8.4 Hz, H2), 8.53 (1H, dd, J=7.2, 0.7 Hz, H7).
^{13}C NMR ($CDCl_3$, 100 MHz): 28.2 (C14), 40.2 (C16), 42.0 (C11), 44.9 (C18), 56.9 (C17), 82.0 (C13), 104.3 (C3), 109.7 (C1), 120.6 (C4a), 122.6 (C8), 124.7 (C6), 127.1 (C5), 130.1 (C8a), 131.5 (C7), 134.9 (C2), 149.9 (C4), 163.8 (C10), 164.5 (C9), 167.8 (C12).
HRMS (ESI) *m/z* calculated for $C_{22}H_{27}N_3O_4.H^+$ 398.2080, found 398.2086.

Preparation of **1C**

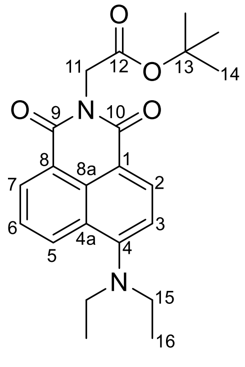

To a solution of intermediate **X** (102 mg, 0.261 mmol, 1 equiv) in 2-methoxyethanol (3 mL) was added $CuSO_4.5H_2O$ (33 mg, 0.131 mmol, 0.5 equiv), diethylamine (532 μL, 5.143 mmol, 19.7 equiv). The mixture was heated at 130°C in a ReactiVial overnight. TLC showed completion of the reaction, and the mixture was poured into water (10 mL). The resulting suspension was extracted with ethyl acetate (20 mL). The combined organic layers were washed with brine, dried over $MgSO_4$, filtered, and evaporated.

Crude product was purified by column chromatography over silica, eluting with 5% EtOAc/Hexane. Relevant fractions were combined and evaporated to obtain the product (7.40 mg, 0.0194 mmol, 7 %) as a yellow solid.
^{1}H NMR ($CDCl_3$, 600 MHz): 1.15 (6H, t, J=7.1 Hz, H16), 1.47 (9H, s, H14), 3.41 (4H, q, H15), 4.82 (2H, s, H11), 7.21 (1H, d, J= 8.1 Hz, H3), 7.65 (1H, dd, J= 7.7, 7.9 Hz, H6), 8.49 (2H, appears d, H5, H2), 8.56 (1H, d, J= 7.2 Hz, H7).
^{13}C NMR ($CDCl_3$, 100 MHz): 12.2 (C16), 28.2 (C14), 42.0 (C11), 47.8 (C15), 82.1 (C13), 117.0 (C3), 115.6 (C1), 122.8 (C8), 125.4 (C6), 127.5 (C4a), 130.5 (C8a), 131.3 (C5), 131.5 (C7), 132.4 (C2), 155.1 (C4) 163.7 (C10), 164.3 (C9), 167.4 (C12)
HRMS (ESI) *m/z* calculated for $C_{22}H_{26}N_2O_4.H^+$ 383.1971, found 383.1978.

Preparation of **1D**

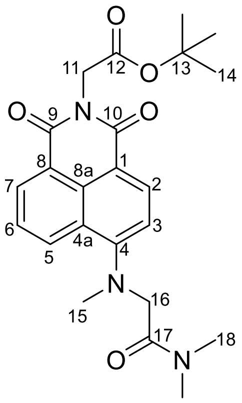

To a solution of intermediate **X** (0.100 g, 0.257 mmol, 1 equiv) in 2-methoxyethanol (2mL) was added $CuSO_4.5H_2O$ (36 mg, 0.128 mmol, 0.5 equiv), triethylamine (54 μL, 0.384 mmol, 1.5 equiv), and N^1, N^1,N^2-trimethylglycinamide (66 μL, 0.513 mmol, 2 equiv). The mixture was heated at 130°C in a ReactiVial overnight. TLC showed completion of the reaction, and the mixture was poured into water (10 mL). The resulting suspension was extracted with ethyl acetate (100 mL). The combined organic layers were washed with brine, dried over $MgSO_4$, filtered, and evaporated. Crude product was purified by column chromatography over silica, eluting with 50 % EtOAc/Hexane and then pure EtOAc. Relevant fractions were combined and evaporated to obtain the product (29 mg, 0.068 mmol, 26%) as a yellow solid.
^{1}H NMR ($CDCl_3$, 600 MHz): 1.46 (9H, s, H14), 2.95 (3H, s, H18a), 3.01 (3H, s, H18b), 3.16 (3H, s, H15), 4.14 (2H, s, H16), 4.81 (2H, s, H11), 7.22 (1H, d, J= 8.1 Hz, H3), 7.63 (1H, appears t, J= 7.4 Hz, H6), 8.41 (1H, dd, J= 7.9 Hz, H5), 8.47 (1H, d, J= 8.1 Hz, H2), 8.55 (1H, dd, J=6.5 Hz, H7).
^{13}C NMR ($CDCl_3$, 100 MHz): 28.1 (C14), 35.9 (18b), 36.6 (18a), 42.1 (C11), 42.3 (C15), 58.8 (C16), 82.1 (C13), 115.1 (C3), 115.4 (C1), 122.9 (C8), 125.45 (C6), 125.54 (C4a), 130.5 (C8a), 131.0 (C5), 131.5 (C7), 132.8 (C2), 156.1 (C4), 163.6 (C10), 164.3 (C9), 167.4 (C12), 168.5 (C17).
HRMS (ESI) *m/z* calculated for $C_{23}H_{27}N_3O_5.H^+$ 426.2029, found 426.2041.

Preparation of **1E**

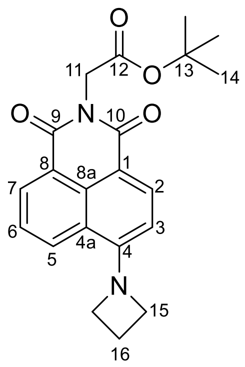

To a solution of intermediate **X** (105 mg, 0.269 mmol, 1 equiv) in 2-methoxyethanol (2.5 mL) was added $CuSO_4.5H_2O$ (32 mg, 0.128 mmol, 0.5 equiv), azetidine hydrochloride (120 mg, 1.28 mmol, 5 equiv), and K_2CO_3 (71 mg, 0.207 mmol, 2 equiv). The mixture was heated at 130°C in a ReactiVial. After 16h, TLC showed completion of the reaction and the mixture was poured into water (10 mL). The resulting suspension was extracted with ethyl acetate (30 mL). The combined organic layers were washed with brine, dried over $MgSO_4$, filtered, and evaporated. Crude product was purified by column chromatography over silica, eluting with 5% EtOAc/Hexane then 50% EtOAc/Hexane. Relevant fractions were combined and evaporated to an orange solid product (20.9 mg, 0.057 mmol, 22 %)

^{1}H NMR ($CDCl_3$, 600 MHz): 1.46 (9H, s, H14), 2.55 (2H, m, H16), 4.47 (4H, t, J= 7.6 Hz, H15), 4.80 (2H, s, H11), 6.34 (1H, d, J=8.6 Hz, H3), 7.46 (1H, dd, J=7.4, 8.4 Hz, H6), 8.21 (1H, dd, J= 1.0, 8.4 Hz, H5), 8.35 (1H, d, J=8.6 Hz, H2), 8.50 (1H, dd, J=1.0, 7.4 Hz, H7).

^{13}C NMR ($CDCl_3$, 100 MHz): 17.1 (C16), 28.2 (C14), 42.1 (C11), 55.4 (C15), 81.9 (C13), 106.2 (C3), 109.6 (C1), 121.0 (C4a), 122.3 (C8), 123.7 (C6), 130.5 (C5), 130.9 (C8a), 131.5 (C7), 133.7 (C2), 152.7 (C4), 163.7 (C10), 164.5 (C9), 167.7 (C12).

HRMS (ESI) *m/z* calculated for $C_{21}H_{22}N_2O_4$. Na^+ 389.1477, found 389.1483.

Preparation of **1F**

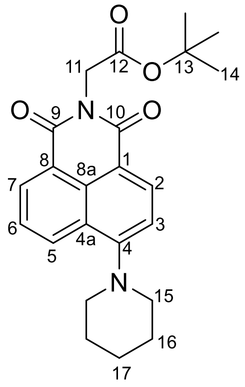

To a solution of intermediate **X** (102 mg, 0.261 mmol, 1 equiv) in 2-methoxyethanol (2 mL) was added $CuSO_4.5H_2O$ (34.3 mg, 0.137 mmol, 0.5 equiv), piperidine (101 mL, 1.03 mmol, 4 equiv), and triethylamine (54 mL, 0.384 mmol, 1.5 equiv). The mixture was heated at 130°C in a ReactiVial. After 16h, TLC showed completion of the reaction, and the mixture was poured into water (10 mL). The resulting suspension was extracted with ethyl acetate (30 mL). The combined organic layers were washed with brine, dried over $MgSO_4$, filtered, and evaporated. Crude product was purified by column chromatography over silica, eluting it with 5% EtOAc/Hexane then 50% EtOAc/Hexane. Relevant fractions were combined and evaporated to a yellow solid product (37.5 mg, 0.0951 mmol, 36 %)

^{1}H NMR ($CDCl_3$, 600 MHz): 1.47 (9H, s, H14), 1.71 (2H, bm, H17), 1.87 (4H, m, H16), 3.22 (4H, appears bm, H15), 4.81 (2H, s, H11), 7.16 (1H, d, J= 8.3 Hz, H3), 7.66 (1H, dd, J= 8.4, 7.2 Hz, H6), 8.39 (1H, dd, J= 8.4, 1.0 Hz, H5), 8.48 (1H, d, J= 8.1 Hz, H2), 8.55 (1H, dd, J=7.2, 1.2 Hz, H7).
^{13}C NMR ($CDCl_3$, 100 MHz): 24.4 (C17), 26.2 (C16), 28.2 (C14), 42.0 (C11), 54.7 (C15), 82.1 (C13), 114.9 (C3), 115.8 (C1), 122.8 (C8), 125.5 (C6), 126.3 (C4a), 130.2 (C8a), 131.0 (C5), 131.4 (C7), 133.0 (C2), 157.3 (C4), 163.7 (C10), 164.3 (C9), 167.4 (C12).
HRMS (ESI) *m/z* calculated for $C_{23}H_{26}N_2O_4.H^+$ 395.1971, found 395.1978.

Preparation of **1G**

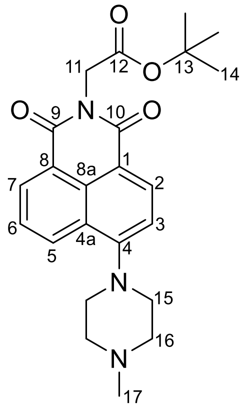

To intermediate **X** (109 mg, 0.278 mmol, 1 equiv) n a ReactiVial was added 1-methyl piperazine (1mL, xs) and the whole stirred overnight at 50°C. TLC showed completion of the reaction and the mixture was poured into water (20 mL). The resulting suspension was extracted with ethyl acetate (40 mL). The combined organic layers were washed with brine, dried over $MgSO_4$, filtered, and evaporated to obtain a yellow solid product (98.7 mg, 0.250 mmol, 90%)
^{1}H NMR ($CDCl_3$, 600 MHz): 1.47(9H, s, H14), 2.52 (3H, s, H17), 2.89 (4H, appears s, H16), 3.37 (4H, bm, H15), 4.81(2H, s, H11), 7.21 (1H, d, J=8.1 Hz, H3), 7.67 (1H, dd, J=8.4, 7.4 Hz, H6), 8.37 (1H, dd, J= 8.4,1.2 Hz, H5), 8.50 (1H, d, J=7.9 Hz, H2), 8.56 (1H, dd, J= 7.2,1.0 Hz, H7).
^{13}C NMR ($CDCl_3$, 100 MHz): 28.2 (C14), 42.1 (C11), 45.6 (C17), 52.3 (C15), 54.9 (C16), 82.2 (C13), 115.3 (C3), 116.7 (C1), 123.0 (C8), 125.9 (C6), 126.3 (C4a), 130.1 (C8a), 130.4 (C5), 131.5 (C7), 132.9 (C2), 155.7 (C4), 163.6 (C10), 164.1 (C9), 167.3 (C12).
HRMS (ESI) *m/z* calculated for $C_{23}H_{27}N_3O_4$. H^+ 410.2080, found 410.2090.

Prepration of **1H**

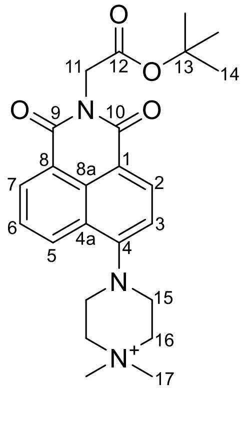

Prepared according to the procedure from.[2] To a solution of **1G** (26 mg, 0.065 mmol) in DMF (1.45 mL) was added methyl iodide (98 µL, 1.574 mmol) and the whole stirred at room temperature overnight.

Diethyl ether (4mL) was added causing formation of a precipitate which was collected by centrifuging at 4500 rpm/min for 5 minutes and discarding the supernatant. The precipitates were then triturated with EtOAc (20 mL), and dried *in vacuo* to obtain an orange solid product (21 mg, 0.049 mmol, 76 %).
^{1}H NMR (CD_3OD, 600 MHz): 1.46 (9H, s, H14), 3.37 (6H, s, H17), 3.62 (4H, appears bm, H15), 3.85 (4H, appears bm, H16), 4.74 (2H, s, H11), 7.48 (1H, d, J=8.1 Hz, H3), 7.78 (1H, dd, J=8.4, 7.4 Hz, H6), 8.45 (1H, d, J= 8.1 Hz, H2), 8.50 (2H, two proton multiple, H2, H7).
^{13}C NMR (CD_3OD, 100 MHz): 27.0 (C14), 41.7 (C11), 46.5 (C15), Under MD_3OD (C17), 61.8 (C16), 82.1(C13), 116.3 (C3), 117.4 (C1), 122.5 (C8), 126.5 (C6), 126.0 (C4a),129.5 (C8a), 130.4 (C5), 131.2 (C7), 132.2 (C2), 154.0 (C4), 163.5 (C10), 163.9 (C9), 167.8 (C12).
HRMS (ESI) *m/z* calculated for $C_{24}H_{30}N_3O_4^+$ 424.2236, found 424.2242.

Preparation of series 2

2A – **2H** were prepared from intermediate **Y** which prepared as previously described and giving identical spectroscopic data to that reported.[3]

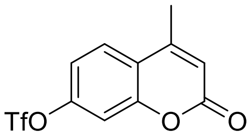

Intermediate **Y**

Preparation of **2A**

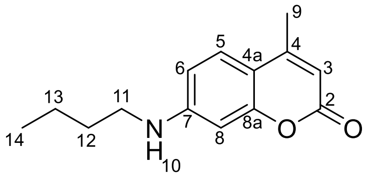

A ReactiVial was charged with intermediate **Y** (151 mg, 0.487 mmol, 1equiv), tris(dibenzylideneacetone) dipalladium (0) (22 mg, 0.024 mmol, 0.05 equiv), Xantphos (28 mg, 0.049 mmol, 0.1 equiv), and Cs_2CO_3 (312 mg, 0.958 mmol, 2equiv). The vial was sealed and flushed with N_2 (x3). Dioxane (4 mL) was mixed with n-butylamine (96 µL, 0.970 mmol, 2 equiv), this solution added to the ReactiVial and the mixture degassed with N_2 The vial was sealed with parafilm and stirred at 100°C overnight. Then,TLC (10:80:10 = DCM/petroleum ether/EtOAc) demonstrated the completion of the reaction, and the mixture was poured into water (15mL). The resulting suspension was extracted with EtOAc (45 mL). The organic layer was washed with brine, dried over $MgSO_4$, filtered, and evaporated. Crude product was purified by column chromatography over silica eluting with (10:80:10 = DCM/ petroleum ether /EtOAc). Relevant fractions were combined and evaporated to an off-white solid product (22.3 mg, 0.091 mmol, 19 %).

^{1}H NMR ($CDCl_3$, 600 MHz): 0.94 (3H, t, J= 7.4 Hz, H14), 1.41 (2H, m, H13), 1.62 (2H, m, H12), 2.31 (3H, d, J=1.0 Hz, H9), 3.15 (2H, t, J= 7.2 Hz, H11), 5.95 (1H, d, J=1.0 Hz, H3), 6.48 (1H, d, J= 2.2 Hz, H8), 6.54 (1H, dd, J= 2.2, 8.6 Hz, H6), 7.33 (1H, d, J=8.6 Hz, H5).
^{13}C NMR ($CDCl_3$, 100 MHz): 13.8 (C13), 18.6 (C9), 20.2 (C12), 30.7 (C11), 44.9 (C10), 100.5 (C8), 110.5 (C3), 112.0 (C6), 112.2 (4a), 125.7 (C5), 149.4 (C7), 152.8 (C4), 155.7 (C8a), 161.7 (C2)
HRMS (ESI) *m/z* calculated for $C_{14}H_{17}NO_2.H^+$ 232.1338, found 232.1349.

Preparation of **2B**

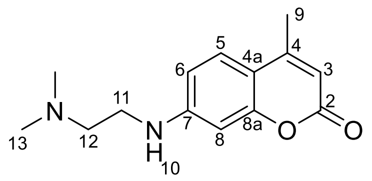

A reactivial was charged with interediate **Y** (150 mg, 0.485 mmol, 1equiv) tris(dibenzylideneacetone) dipalladium (0) (22 mg, 0.024 mmol, 0.05 equiv), Xantphos (28 mg, 0.049 mmol, 0.1 equiv), and Cs_2CO_3 (316 mg, 0.970 mmol, 2 equiv). The vial was sealed and flushed with N_2 (3x). Dioxane (4 mL) was mixed with N,N-dimethylethylenediamine (96 µL, 0.970 mmol, 2 equiv), this solution added to the ReactiVial and the mixture degassed with N_2. The vial was sealed with parafilm and stirred at 100°C overnight. Then TLC (98:2:0.5 = DCM/MeOH/880 NH_3) demonstrated the completion of the reaction, and the mixture was poured into water (15 mL). The resulting suspension was extracted with EtOAc (45 mL). The organic layer was washed with brine, dried over $MgSO_4$, filtered, and evaporated. Crude product was purified by column chromatography over silica eluting with 97:3:0.5 (DCM/MeOH/880 NH_3). Relevant fractions were combined and evaporated to an off-white solid product (19.7 mg, 0.080 mmol, 16 %).

^{1}H NMR (CD_3OD, 600 MHz): 2.29 (6H, s, H13), 2.34 (3H, appears d, J= 1.0 Hz, H9), 2.57 (2H, t, H12), 3.26 (2H, appears d, J= 6.9 Hz, H11), 3.28 (1H, appears quintet, H10) 5.90 (1H, Appears d, J=1.0 Hz, H3), 6.41 (1H, d, J= 2.2 Hz, H8), 6.61 (1H, dd, J= 2.2, 8.8 Hz, H6), 7.42 (1H, d, J= 8.8 Hz, H5).
^{13}C NMR (CD_3OD, 100 MHz): 17.2 (C9), 40.3 (C11), 44.2 (C13), 57.5 (C12), 96.6 (C8), 107.3 (C3), 109.7 (C4a), 110.6 (C6), 125.6 (C5), 152.7 (C7), 155.1 (C4), 156.0 (C8a), 163.3 (C2).
HRMS (ESI) *m/z* calculated for $C_{14}H_{18}N_2O_2.H^+$ 247.1447, found 247.1456.

2C was purchased from Fluorochem.

Preparation of **2D**

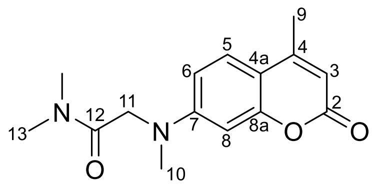

A ReactiVial was charged with intermediate **Y** (154 mg, 0.498 mmol, 1 equiv), $Pd_2(dba)_3$ (24 mg, 0.026 mmol, 0.05 equiv), XantPhos (31 mg, 0.053 mmol, 0.1 equiv), and Cs_2CO_3 (311 mg, 0.956 mmol, 2 equiv). The vial was sealed and flushed with N_2 (3x). Dioxane (4 mL) was mixed with N^1,N^1,N^2-trimethylglycinamide (125 µL, 0.970 mmol, 2 equiv) this solution added to the ReactiVial and the mixture degassed with N_2. The vial was sealed with parafilm and stirred at 100°C overnight. TLC (EtOAc) demonstrated the completion of the reaction, and the mixture was poured into water (10 mL). The resulting suspension was extracted with EtOAc (45 mL). The organic layer was washed with brine, dried over $MgSO_4$, filtered, and evaporated. Crude product (EtOAc) was purified by column chromatography over silica, eluting with EtOAc. Relevant fractions were combined and evaporated to an off-white solid product (13.8 mg, 0.050 mmol, 10 %).

^{1}H NMR ($CDCl_3$, 600 MHz): 2.32 (3H, d, J= 1.0 Hz, H9), 2.97 (3H, s, H10) 3.1 (6H, s, H13), 4.18 (2H, s, H11), 5.96 (1H, d, J=1.0 Hz, H3), 6.43 (1H, appears d, J=2.8 Hz, H8), 6.59 (1H, dd, J=2.6, 8.9 Hz, H6), 7.39 (1H, d, J= 8.9 Hz, H5).

^{13}C NMR ($CDCl_3$, 100 MHz): 18.6 (C9), 35.8 (C10), 36.4 (13a), 40.0 (13b), 53.8 (C11), 98.8 (C8), 109.1 (C6), 109.7 (C3), 110.4 (C4a), 125.6 (C5), 152.5 (C7), 153.0 (C4), 155.7 (C8a), 162.2 (C2), 168.0 (C12).
HRMS (ESI): $C_{15}H_{18}N_2O_3.H^+$ requires 275.1396, found 275.1396

Preparation of **2E**

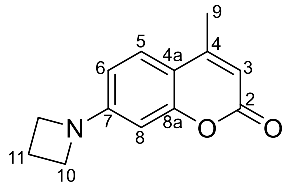

A reactivial was charged with intermediate **Y** (149 mg, 0.483 mmol, 1equiv), RuPhos (22 mg, 0.049 mmol, 0.1 equiv), RuPhos-G3-palladacycle (41 mg, 0.049 mmol, 0.1 equiv), and K_2CO_3 (112 mg, 0.809 mmol, 1.7 equiv). The vial was sealed and flushed with N_2 (3x). Dioxane (4 mL) was mixed with Azetidine (36 µL, 0.534 mmol, 1.1 equiv) this solution added to the ReactiVial and the mixture degassed with N_2. The vial was sealed with parafilm and stirred at 100°C overnight. TLC 1:99 (EtOAc:DCM) demonstrated the completion of the reaction, and the mixture was poured into water (15mL). The resulting suspension was extracted with EtOAc (45 mL). The organic layer was washed with brine, dried over $MgSO_4$, filtered, and evaporated. Crude product was purified by column chromatography over silica (50mL) eluting with 1:99 (EtOAc: DCM). Relevant fractions were combined and evaporated to an off-white solid product (48.6 mg, 0.226 mmol, 47 %).

^1H NMR ($CDCl_3$, 600 MHz): 2.31 (3H, d, J= 1.0 Hz, H9), 2.41 (2H, p, H11), 3.96 (4H, t, H10), 5.93 (1H, Appears d, J=1.20 Hz, H3), 6.18 (1H, d, J= 2.2 Hz, H8), 6.28 (1H, dd, J= 2.2, 8.6 Hz, H6), 7.34 (1H, d, J= 8.8 Hz, H5).
^{13}C NMR ($CDCl_3$, 100 MHz): 16.6 (C11), 18.6 (C9), 51.8 (C10), 97.1 (C8), 107.7 (C6), 109.4 (C3), 110.3 (C4a), 125.4 (C5), 153.1 (C4), 154.0 (C7), 155.7 (C8a), 162.0 (C2).
HRMS (ESI): $C_{13}H_{13}NO_2.H^+$ requires 216.1025, found 216.1025

Preparation of **2F**

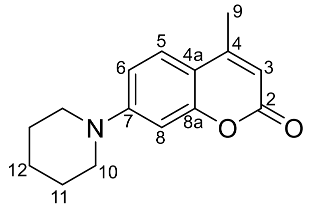

A reactivial was charged with intermediate **Y** (149 mg, 0.485 mmol, 1equiv), RuPhos (22 mg, 0.048 mmol, 0.1 equiv), RuPhos-G3-palladacycle (41 mg, 0.049 mmol, 0.1 equiv), and K_2CO_3 (112 mg, 0.809 mmol, 2equiv). The vial was sealed and flushed with N_2 (3x). Dioxane (4 mL) was mixed with piperdine (72 µL, 0.728 mmol, 1.5 equiv) this solution added to the ReactiVial and the mixture degassed with N_2. The vial was sealed with parafilm and stirred at 100°C overnight. TLC (DCM) demonstrated the completion of the reaction, and the mixture was poured into water (15mL). The resulting suspension was extracted with EtOAc (45 mL). The organic layer was washed with brine, dried over $MgSO_4$, filtered, and evaporated. Crude product was purified by column chromatography over silica, eluting with DCM, then (2-4 % EtOAc/DCM). Relevant fractions were combined and evaporated to an off-white solid product (35.6 mg, 0.146 mmol, 30 %).

^{1}H NMR ($CDCl_3$, 600 MHz): 1.64 (6H, appears bm, H11, H12), 2.32 (3H, d, J= 1.1 Hz, H9), 3.31 (4H, t, J=10.8 Hz, H10), 5.98 (1H, d, J=1.0 Hz, H3), 6.68 (1H, s, H8), 6.80 (1H, d, J= 7.7 Hz, H6), 7.37 (1H, d, J= 8.9 Hz, H5).
^{13}C NMR ($CDCl_3$, 100 MHz): 18.5 (C9), 25.3 (C11), 49.1 (C10), 101.2 (C8), 110.3 (C3), 111.0 (C4a), 111.6 (C6), 125.3 (C5), 152.7 (C4), 153.8 (C7),155.7 (C8a), 162.0 (C2)
HRMS (ESI) *m/z* calculated for $C_{15}H_{17}NO_2.H^+$ 244.1338, found 244.1339.

Preparation of **2G**

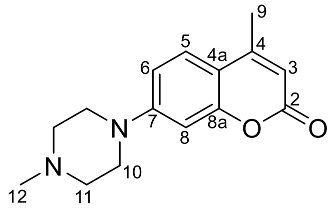

A reactivial was charged with intermediate **Y** (150 mg, 0.485 mmol, 1equiv), RuPhos (11 mg, 0.024 mmol, 0.05 equiv), RuPhos-G3-palladacycle (20 mg, 0.024 mmol, 0.05 equiv), and K_2CO_3 (94 mg, 0.679 mmol, 1.4 equiv). The vial was sealed and flushed with N_2 (3x). Dioxane (4 mL) was mixed with N-methylpiperazine (59 µL, 0.534 mmol, 1.1 equiv) this solution added to the ReactiVial and the mixture degassed with N_2. The vial was sealed with parafilm and stirred at 100°C overnight. TLC (95:5:0.5 = DCM/MeOH/880 NH_3) demonstrated the completion of the reaction, and the mixture was poured into water (15mL). The resulting suspension was extracted with EtOAc (45 mL). The organic layer was washed with brine, dried over $MgSO_4$, filtered, and evaporated. Crude product was purified by column chromatography over silica, eluting with DCM, then 96:4:0.5 (DCM/MeOH/880 NH_3). Relevant fractions were combined and evaporated to an off-white solid product (44 mg, 0.170 mmol, 35 %).

^{1}H NMR ($CDCl_3$, 600 MHz): 2.35 (3H, appears d, J= 0.9 Hz, H9), 2.43 (3H, s, H12), 2.68 (4H, Appears s, H11), 3.42 (4H, Appears s, H10), 6.04 (1H, Appears d, J=0.7 Hz, H3), 6.71 (1H, d, J= 2.4 Hz, H8), 6.80 (1H, dd, J= 2.6, 8.9 Hz, H6), 7.42 (1H, d, J= 8.9 Hz, H5).
^{13}C NMR ($CDCl_3$, 100 MHz): 18.5 (C9), 45.8 (C12), 47.3 (C10), 54.5 (C11), 101.7 (C8), 111.0 (C3), 111.5 (C6), 112.0(C4a), 125.4 (C5), 152.5 (C4), 153.3 (C7), 155.5 (C8a), 161.7 (C2).
HRMS (ESI): $C_{15}H_{18}N_2O_2.H^+$ requires 259.1447, found 259.1449

Preparation of **2H**

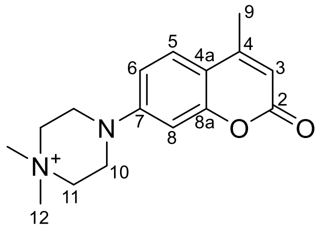

To a solution of **2G** (32 mg, 0.124 mmol) in dimethylformamide (1.7 mL) was added methyl iodide (100 µL, 1.606 mmol). The solution was stirred at room temperature overnight. Diethyl ether (100 mL) was added and the resulting precipitate collected by centrifugation at 4500 rpm/min for 5 minutes and discarding the supernatant. The precipitate was then triturated in EtOAc (20 mL) to obtain product as an off-white solid (19 mg, 0.0702 mmol, 57 %).

^{1}H NMR (DMSO, 600 MHz): 2.38 (3H, s, H9), 3.21 (6H, s, H12), 3.55 (4H, t, H10), 3.73 (4H, t, H11), 6.12 (1H, s, H3), 6.95 (1H, d, H8), 7.06 (1H, dd, J= 2.6, 8.9 Hz, H6), 7.64 (1H, d, J=8.9 Hz, H5).
^{13}C NMR (DMSO, 100 MHz): 18.0 (C9), 40.9 (C11), 50.4 (C12), 59.7 (C10), 100.9 (C8), 110.2 (C4a), 111.4 (C3), 111.4 (C6), 126.1 (C5), 152.0 (C7), 153.3 (C4), 154.8 (C8a), 160.3 (C2).
HRMS (ESI) *m/z* calculated for $C_{16}H_{21}N_2O_2^+$ 273.1603, found 273.1606

Preparation of series 3

3A – **3G** were prepared from intermediate **Z** purchased from Fluorochem using the following general procedure: To a stirred solution of intermediate **Z** (100mg, 0.5 mmol, 1 equiv) in DCM (5 mL) was added amine (4 equiv). After 2 hours solvent was removed in vacuo and the crude product purified by column chromatography over silica.

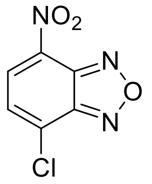

Intermediate **Z**

Preparation of **3A**

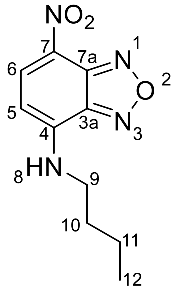

Using 197 µL n-butylamine and 30% ethyl acetate / petroleum ether for chromatography yielded **3A** as an orange solid (47.2 mg, 0.2 mmol, 40%)

^{1}H NMR (DMSO, 600 MHz): 0.92 (3H, t, J=7.4 Hz, H12), 1.39 (2H, m, H11), 1.66 (2H, m, H10), 3.47 (2H, appears bm, H9), 6.41 (1H, d, J= 8.8 Hz, H5), 8.51 (1H, d, J= 8.6 Hz, H6), 9.54 (1H, bs, H8).
^{13}C NMR (DMSO, 100 MHz): 13.6 (C12), 19.6 (C11), 29.7 (C10), 43.1 (C9), 99.1 (C5), 120.5 (C4), 137.9 (C6), 144.2 (C7a), 144.4 (C3a), 145.1 (C7).
HRMS (ESI) *m/z* calculated for $C_{10}H_{12}N_4O_3.H^+$ 273.0988, found 237.0984.

Preparation of **3B**

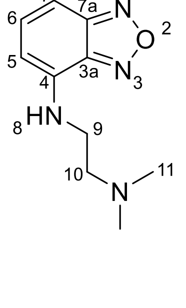

Using 217 µL N,N-dimethylethylene diamine and 98.71.25:0.125 followed by 95:5:0.5 DCM/MeOH/880NH_3 for chromatography yielded **3B** as an orange solid (28.3 mg, 0.113 mmol, 22%)

^{1}H NMR (DMSO, 600 MHz): 2.23 (6H, s, H11), 2.61 (2H, t, J=6.5 Hz, H10), 3.36 (2H, bm, H9), 3.57 (1H, bs, H8), 6.43 (1H, d, J=7.6 Hz, H5), 8.50 (1H, d, J=7.7 Hz, H6)
^{13}C NMR (DMSO, 100 MHz): 41.3 (C9), 45.0 (C11), 56.4 (C10), 99.2 (C5), 120.7 (C4), 137.8.4 (C6), 144.1 (C7a), 144.4 (C3a), 145.1 (C7).
HRMS (ESI) *m/z* calculated for $C_{10}H_{13}N_5O_3.H^+$ 252.1099, found 252.1097

Preparation of **3C**

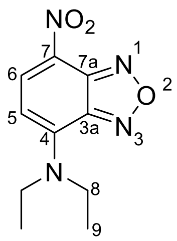

Using 173 µL Et_2NH and 40% ethyl acetate / petroleum ether for chromatography yielded **3C** as an orange solid (66 mg, 0.28 mmol, 56%)

^{1}H NMR (DMSO, 600 MHz): 1.30 (6H, t, J=7.1 Hz, H9), 3.97 (4H, broad singlet, H8), 6.45 (1H, d, J=9.3 Hz, H5), 8.45 (1H, d, J=9.3 Hz, H6)
^{13}C NMR (DMSO, 100 MHz): 12.2 (C9), 47.8 (C8), 102.0 (C5), 119.6 (C4), 136.3 (C6), 144.2 (C3a), 144.96 (C7a), 144.98 (C7).
HRMS (ESI) *m/z* calculated for $C_{10}H_{12}N_4O_3.H^+$ 237.0991, found 237.0988

Preparation of **3D**

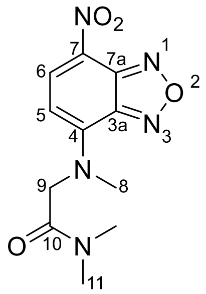

Using 129 µL N^1,N^1,N^2-trimethylglycinamide and 40% ethyl acetate / petroleum ether for chromatography yielded **3C** as an orange solid (113 mg, 0.46 mmol, 91%)

HRMS (ESI) $C_{11}H_{13}N_5O_4$ $[M + H]^+$ requires 280.1042, found 280.1046

Preparation of **3E**

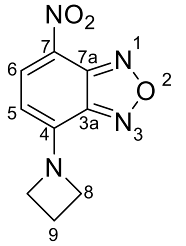

Using 135 µL azetidine and 30% ethyl acetate / petroleum ether for chromatography yielded **3C** as an orange solid (17 mg, 0.075 mmol, 15%)

^{1}H NMR (DMSO, 600 MHz): 2.57 (2H, m, H10), 4.42 (2H, s, H8a), 4.77 (2H, s, H8b), 6.05 (1H, d, J=9.1 Hz, H5), 8.46 (1H, d, J=8.9 Hz, H6).
^{13}C NMR (DMSO, 100 MHz): 16.5 (C10), 53.0 (C9), 56.4 (C8), 99.4 (C5), 119.1 (C4), 136.4 (C6), 143.7 (3a), 144.6 (7a), 145.2 (C7).
HRMS (ESI) $C_9H_8N_5O_3$ [M + H]$^+$ requires 221.0675, found 221.0675

Preparation of **3F**

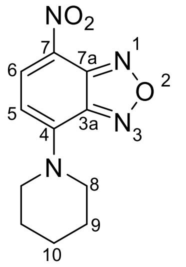

Using 186 µL piperidine and 40% ethyl acetate / petroleum ether for chromatography yielded **3C** as an orange solid (57.1 mg, 0.23 mmol, 46%)
^{1}H NMR (DMSO, 600 MHz): 1.75 (6H, s, H9, H10), 4.15 (4H, s, H8), 6.65 (1H, d, J=9.3 Hz, H5), 8.45 (1H, d, J=9.3 Hz, H6).
^{13}C NMR (DMSO, 100 MHz): 23.4 (C10), 25.8 (C9), 51.0 (C8), 103.1 (C5), 120.1 (C4), 136.4 (C6), 144.8 (C3a), 145.0 (C7a), 145.2 (C7)
HRMS (ESI) *m/z* calculated for $C_{11}H_{12}N_4O_3.H^+$ 249.0988, found 249.0988

Preparation of **3G**

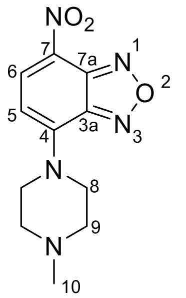

Using 222 µL N-methyl piperazine and 98.7:1.25:0.125 DCM/MeOH/880NH_3 for chromatography yielded **3C** as an orange solid (43.6 mg, 0.166 mmol, 33%)

^1H NMR (DMSO, 600 MHz): 2.25 (3H, appears s, H10), 2.56 (4H, t, J=5.0 Hz, H9), 4.13 (4H, appears s, H8), 6.69 (1H, d, J=9.1 Hz, H5), 8.48 (1H, d, J=9.1 Hz, H6)
^{13}C NMR (DMSO, 100 MHz): 45.2 (C10), 49.2 (C8), 54.2 (C9), 103.6 (C5), 121.2 (C4), 136.3 (C6), 144.8 (C3a), 144.9 (C7a), 145.4 (C7)
HRMS (ESI) $C_{11}H_{13}N_5O_3.H^+$ 264.1097, found 264.1098

Preparation of **3H**

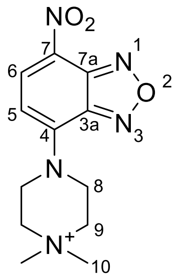

To a stirred solution of 3G (35.7 mg, 0.13 mmol, 1 equiv) in DMF (1.5 mL) was added methyl iodide (98 μL, 1.6 mmol, 12 equiv) and the whole stirred overnight. Diethyl ether (20 mL) was added and the resulting precipitate collected by centrifugation at 4500 rpm/min for 5 minutes and discarding the supernatant. The precipitate was then triturated in EtOAc (20 mL) to obtain product as an orange solid (39 mg, 0.13 mmol, quantitiative).

^1H NMR (DMSO, 600 MHz): 3.26 (6H, s, H10), 3.69 (4H, t, J=5.3 Hz, H9), 4.44 (4H, t, J=4.8 Hz, H8), 6.81 (1H, d, J=9.1 Hz, H5), 8.62 (1H, d, J=8.9 Hz, H6).
^{13}C NMR (DMSO, 100 MHz): 43.1 (C8), 50.9 (C10), 59.7 (C9), 105.1 (C5), 123.4 (C4), 136.1 (C6), 144.5 (C3a), 144.6 (C7a), 144.8 (C7).
HRMS (ESI) *m/z* calculated for $C_{12}H_{16}N_5O_3^+$ 278.1253, found 278.1256.

Photophysical experiments

Photophysical experiments were conducted at 20 °C (or the indicated temperature) in a quartz cuvette using an Edinburgh Instruments FS5 fluorimeter fitted with an SC-25 temperature contolled sample holder and operated with Fluoracle software. Step size was 1 nm and dwell time was 0.2 s; solvents were standard grade and stored over activated 4 Å sieves. **1A** – **3H** were stored as 1 mM solutions in requisite solvents at 4 °C and diluted as necessary. For fluorescence experiments the concentrations and slit widths are shown below. Quantum yields were calculated by reference to quinine sulfate in 0.05M H_2SO_4 (λ_{ex} = 350nm, φ = 0.59).[4]

Series	Concentration	Excitation slit width	Emission slit width
**1**	5 μM	1 nm	1 nm
**2**	1 μM	1 nm	1 nm
**3**	1 μM	2 nm	2 nm

Table S1 Concentrations and slit widths used for fluorescence experiments

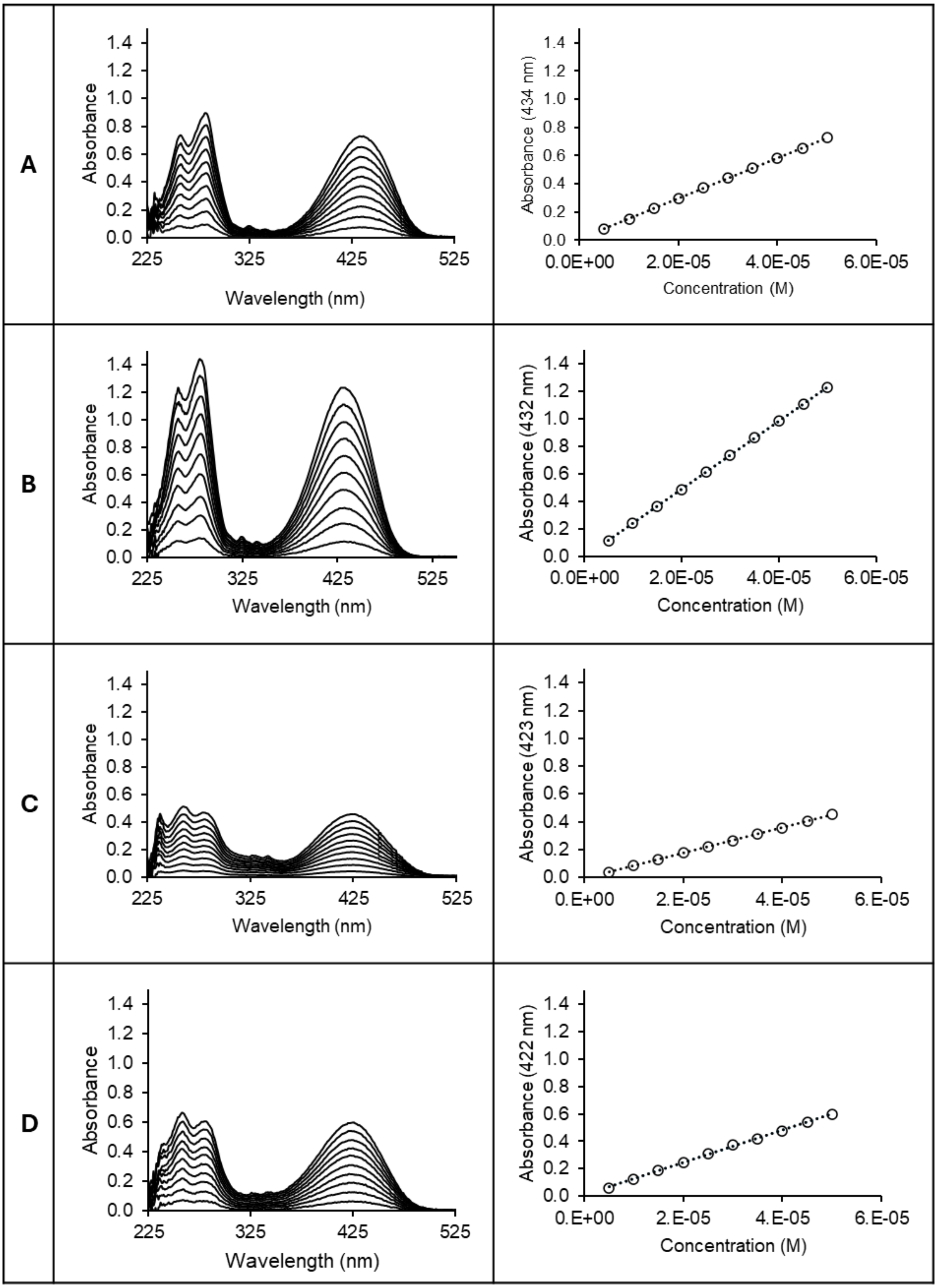
A
Absorbance
Wavelength (nm)
Absorbance (434 nm)
Concentration (M)
B
Absorbance
Wavelength (nm)
Absorbance (432 nm)
Concentration (M)
C
Absorbance
Wavelength (nm)
Absorbance (423 nm)
Concentration (M)
D
Absorbance
Wavelength (nm)
Absorbance (422 nm)
Concentration (M)

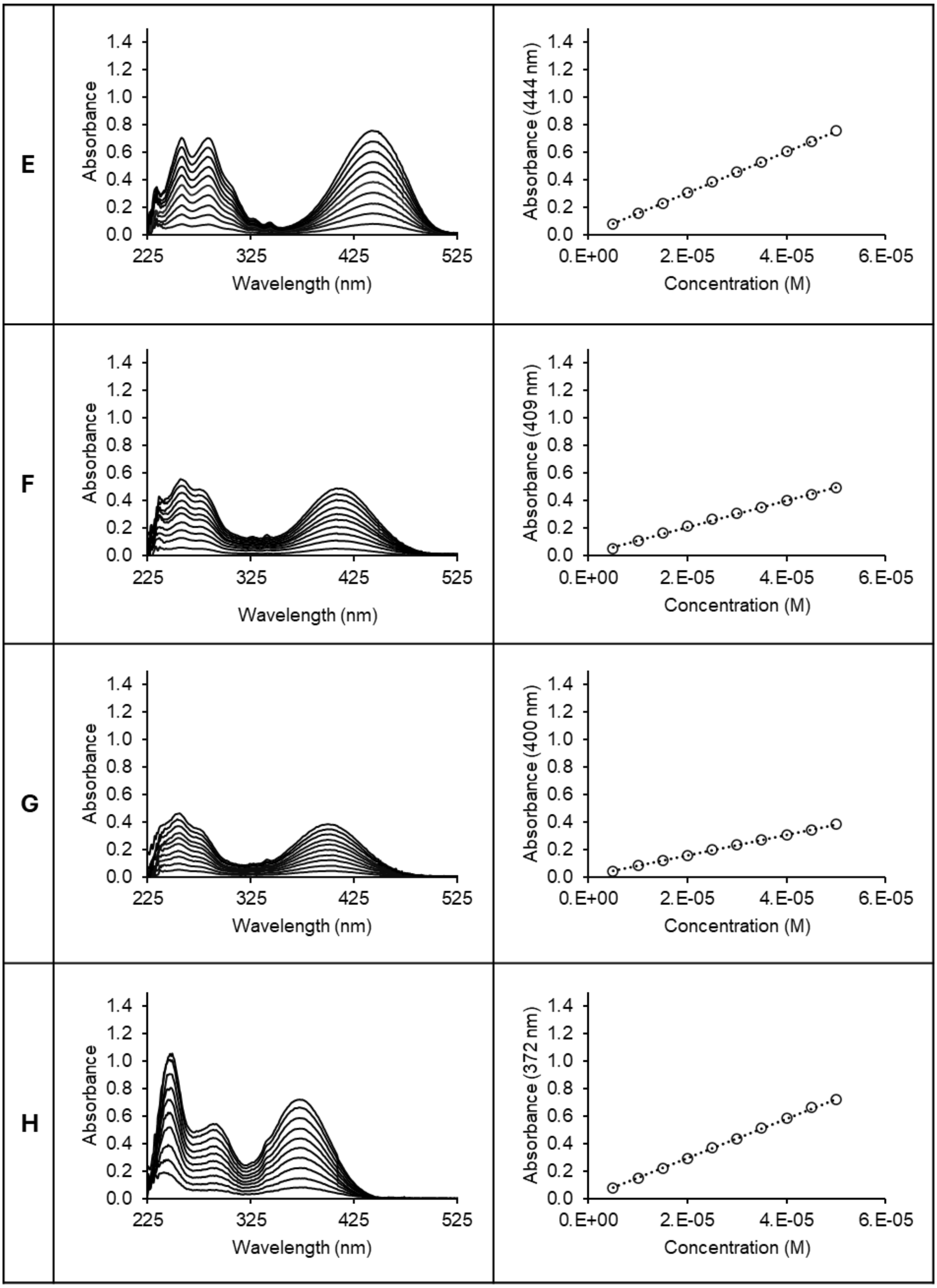

Figure S1 Absorbance and extinction co-efficient data for series **1** in MeCN (5 – 50 µM, this and preceeding pages)

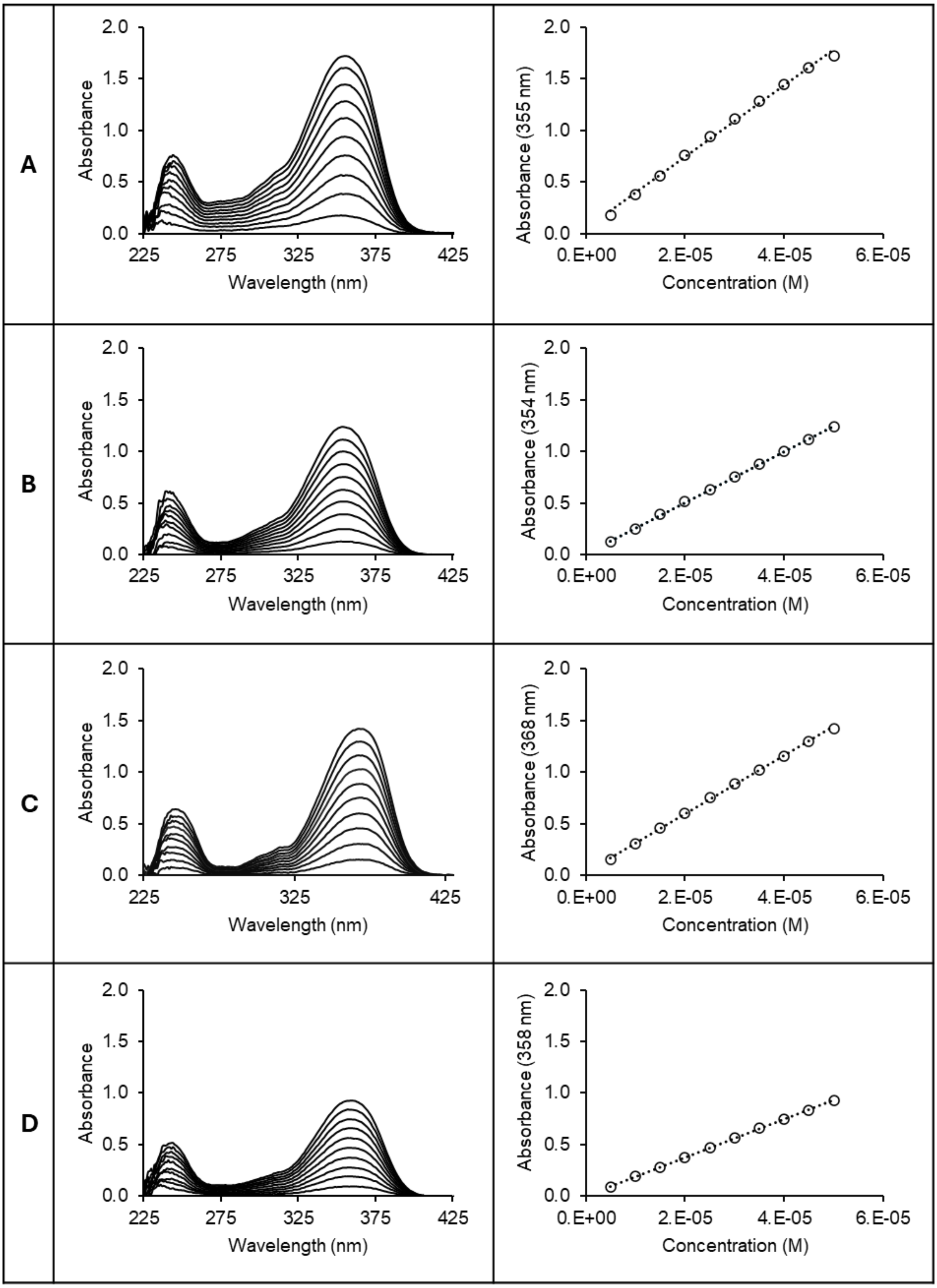
A
B
C
D
Absorbance
Wavelength (nm)
Absorbance (355 nm)
Absorbance (354 nm)
Absorbance (368 nm)
Absorbance (358 nm)
Concentration (M)
0.0
0.5
1.0
1.5
2.0
225
275
325
375
425
0.E+00
2.E-05
4.E-05
6.E-05

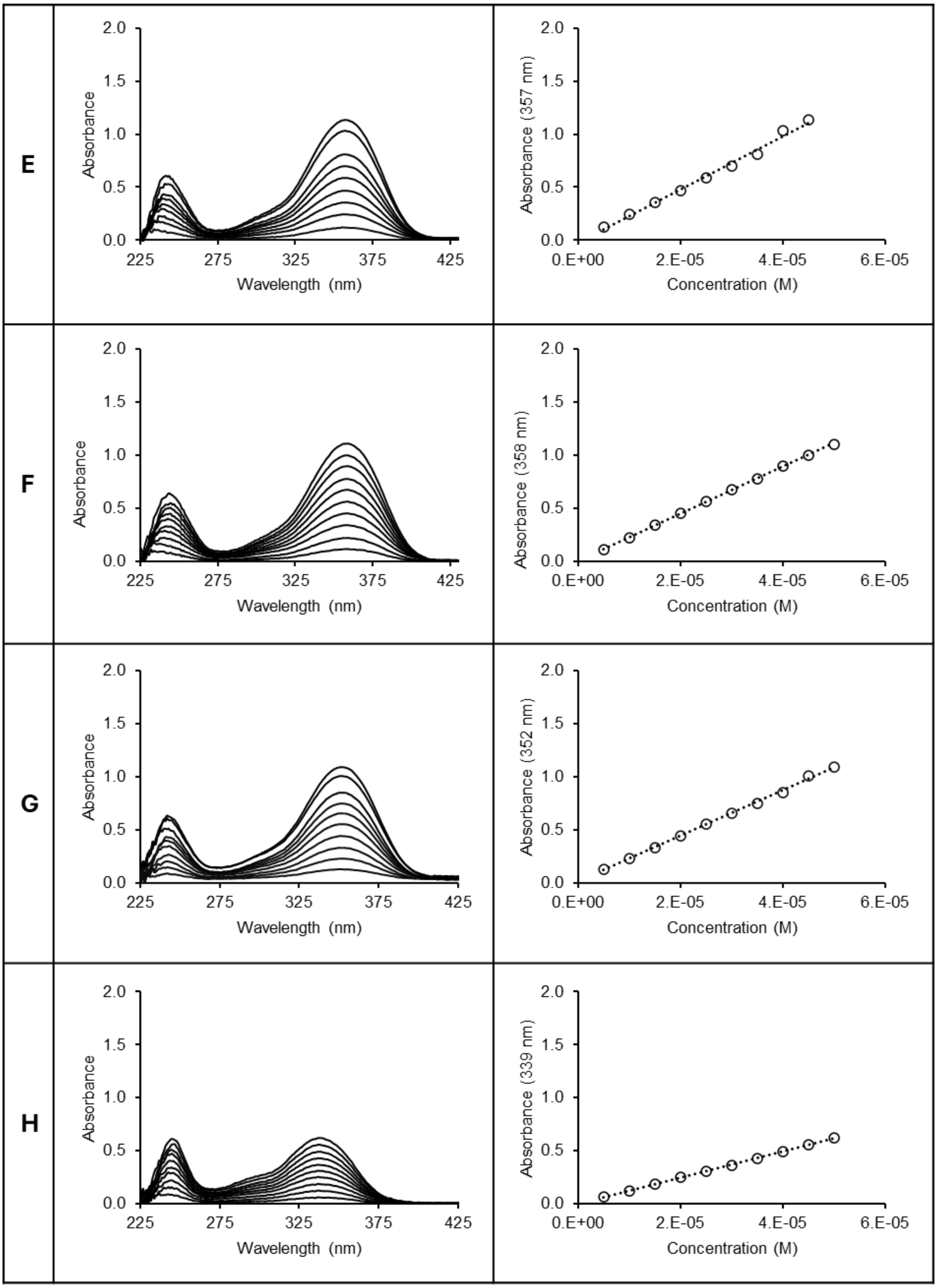

Figure S2 Absorbance and extinction co-efficient data for series **2** in MeCN (5 – 50 μM, this and preceeding pages)

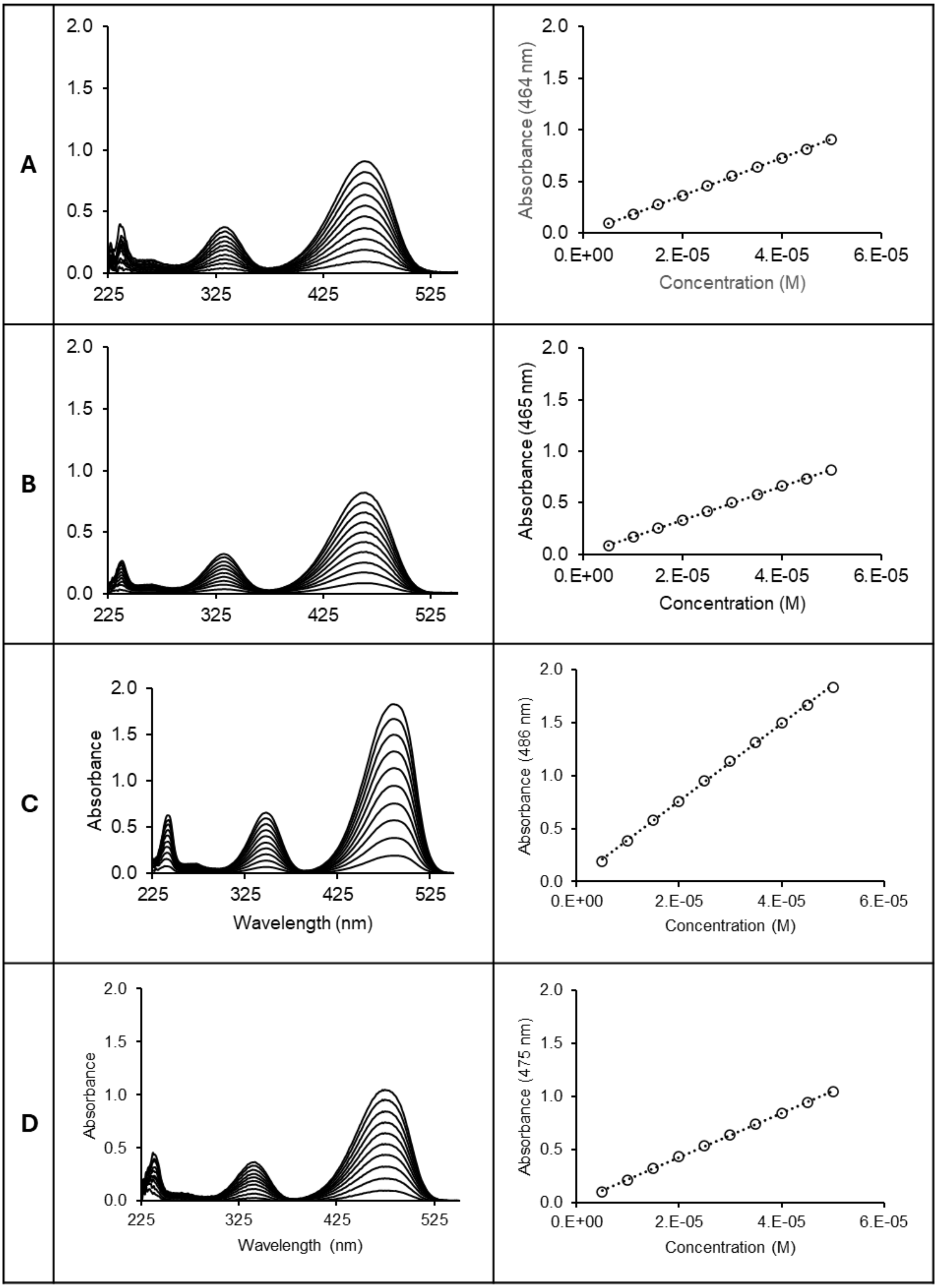
A
B
C
D
Absorbance
Wavelength (nm)
Absorbance (464 nm)
Absorbance (465 nm)
Absorbance (486 nm)
Absorbance (475 nm)
Concentration (M)
2.0
1.5
1.0
0.5
0.0
225
325
425
525
0.E+00
2.E-05
4.E-05
6.E-05

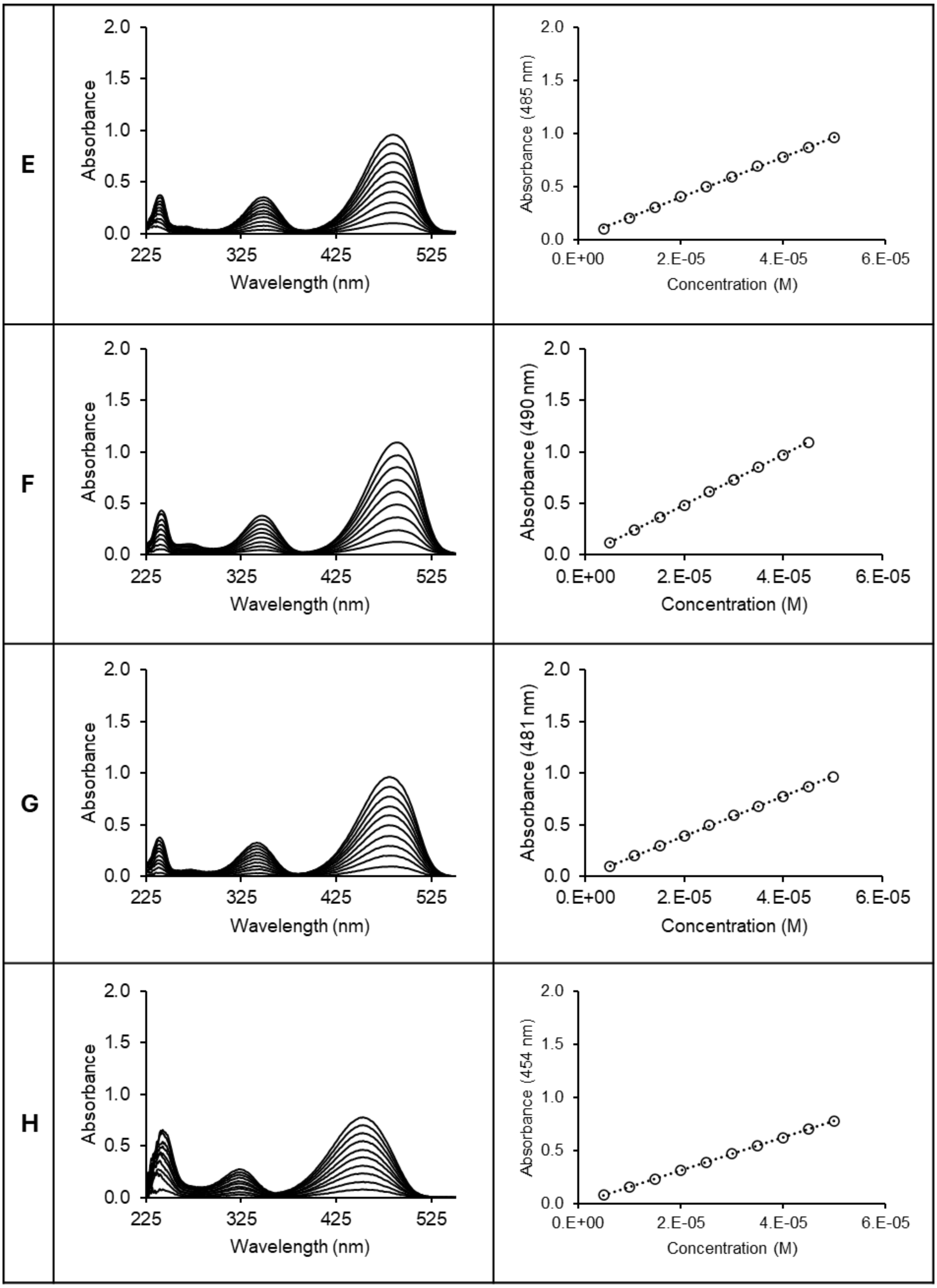

Figure S3 Absorbance and extinction co-efficient data for series **3** in MeCN (5 – 50 μM, this and preceeding pages)

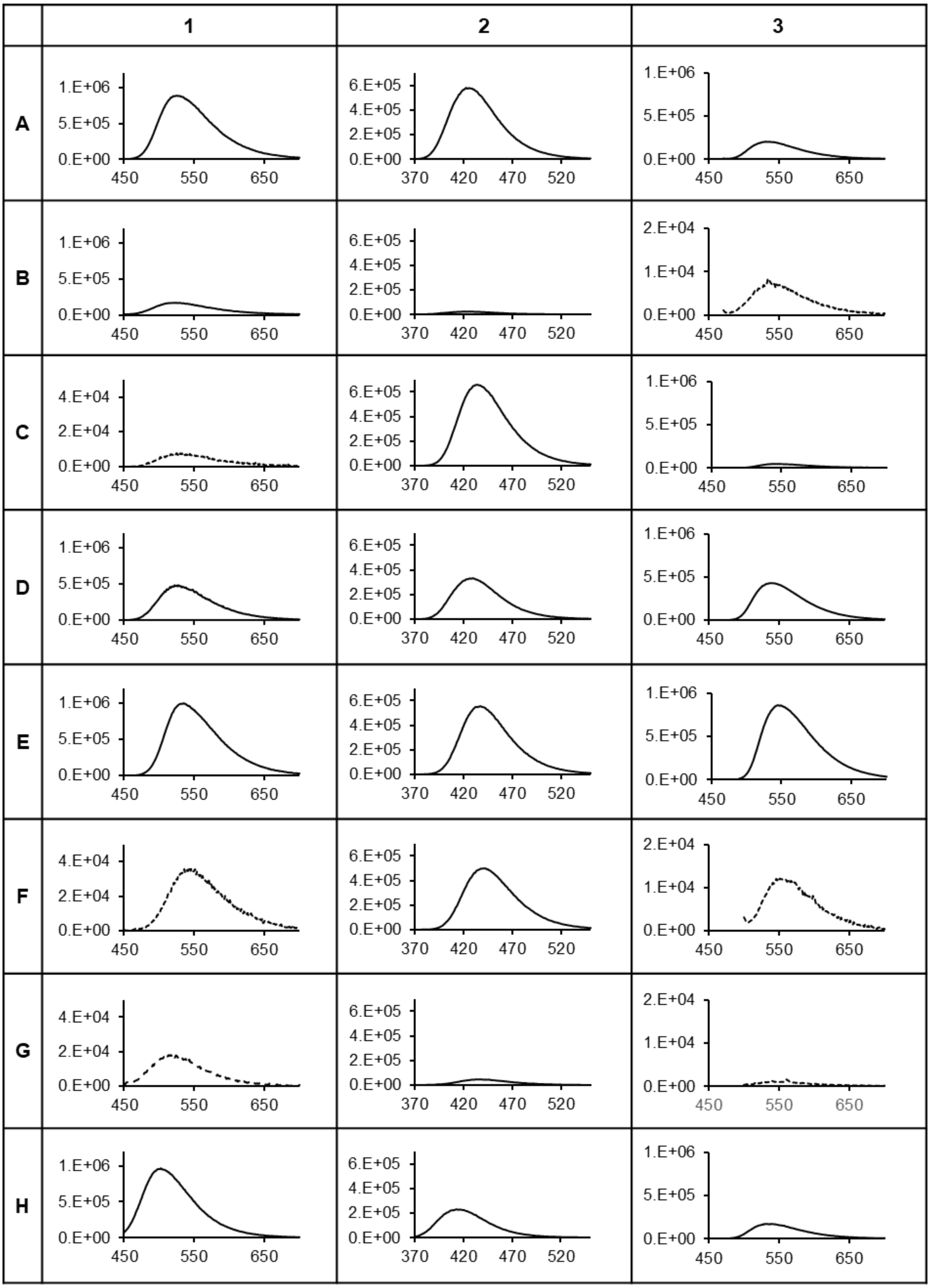

Figure S4 Emission spectra in MeCN recorded under conditions shown in Table S1. Note change in vertical scale for spectra shown with a dotted line.

Fluorescence variation with temperature

Fluorescence spectra of MeCN solutions of compounds **1A** – **3H** were obtained using the parameters indicated in Table S1 irradiating at λ_{abs} (Table 1) at temperatures between 5 and 60°C in 5°C increments ensuring the temperature was stable for at least 5s before recording a spectrum. Normalised emission at λ_{em} (Table 1) was plotted against temperature.

Fluorescence variation with pH

Solutions of compounds **1A** – **3H** at the concentrations indicated in Table S1 were prepared in 1:1 MeCN/TRIS (10 mM) where the TRIS had been adjusted to pH ~ 11. A fluorescence spectrum was recorded using the parameters indicated in Table S1 and using λ_{ex} for MeCN. Sulfuric acid 10 mM then 100 mM was added in aliquots of 0.5 – 10 µL; the pH and an emission spectrum were recorded after each addition. Total volume added was < 5% of the initial volume meaning that dilution effects could be neglected. Normalised emission at λ_{em} (Table 1) was plotted against pH.

<table>
<tr><th></th><th>B</th><th>G</th></tr>
<tr><td>1</td><td>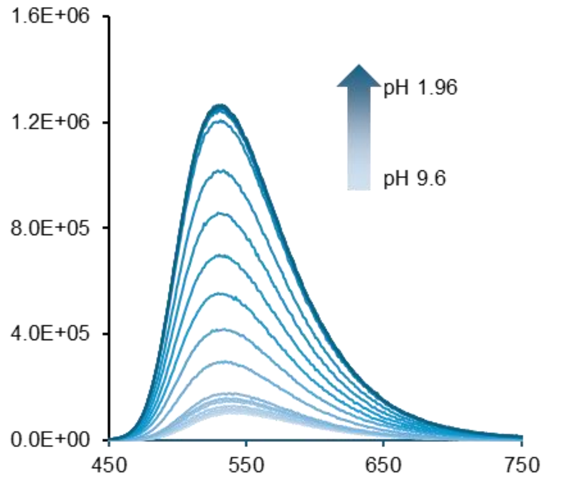
</td><td>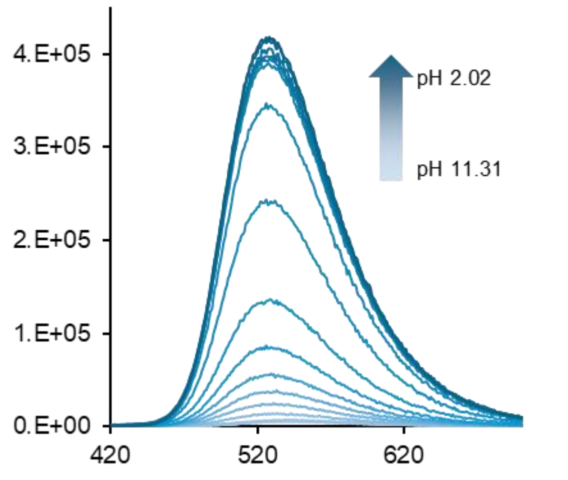
</td></tr>
<tr><td>2</td><td>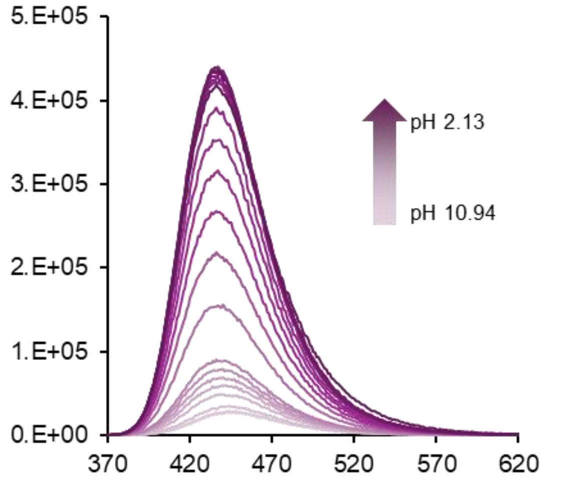
</td><td>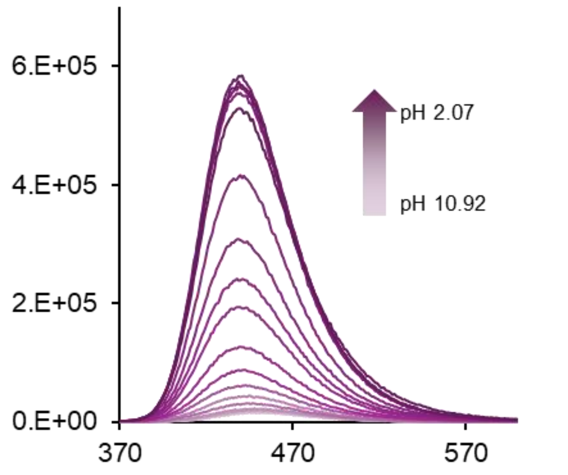
</td></tr>
<tr><td>3</td><td>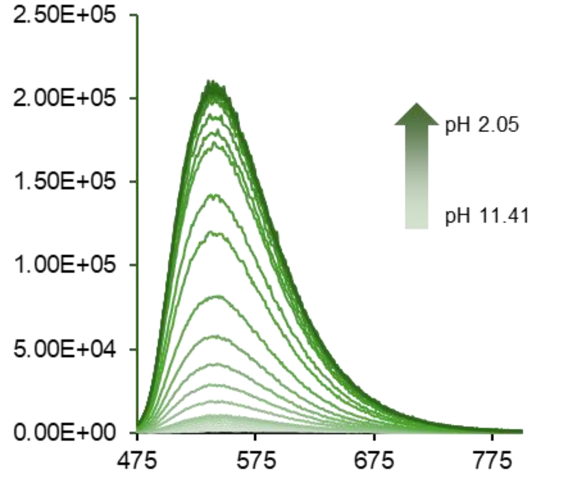
</td><td>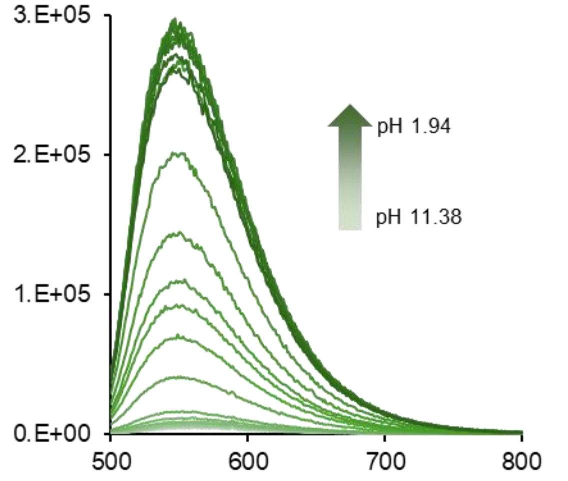
</td></tr>
</table>

<table>
<tr><th></th><th>A</th><th>F</th></tr>
<tr><td>1</td><td>Not shown</td><td>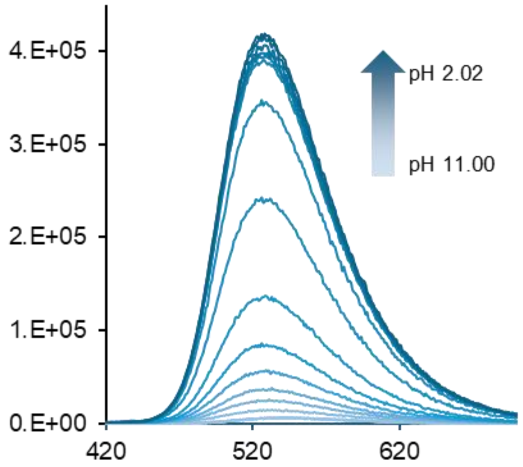
</td></tr>
<tr><td>2</td><td>Not shown</td><td>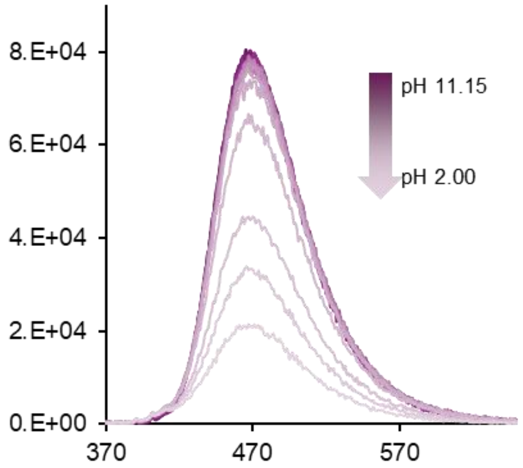
</td></tr>
<tr><td>3</td><td>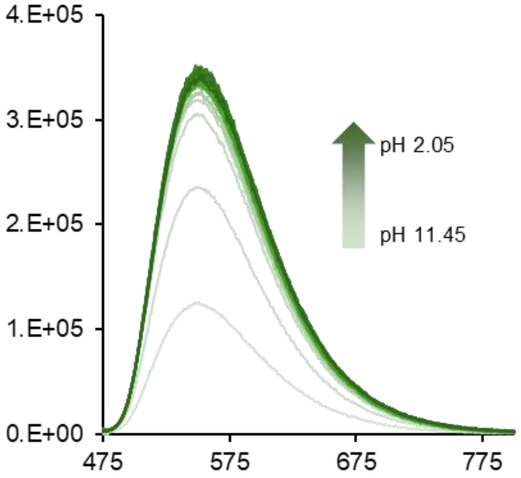
</td><td>Not shown</td></tr>
</table>

Figure S5 Fluorescence spectra from pH titration experiments conducted under conditions given in Table S1. Wavelength is shown on the x-axes and fluorescence on the y-axes (this and preceding pages).

Series 1

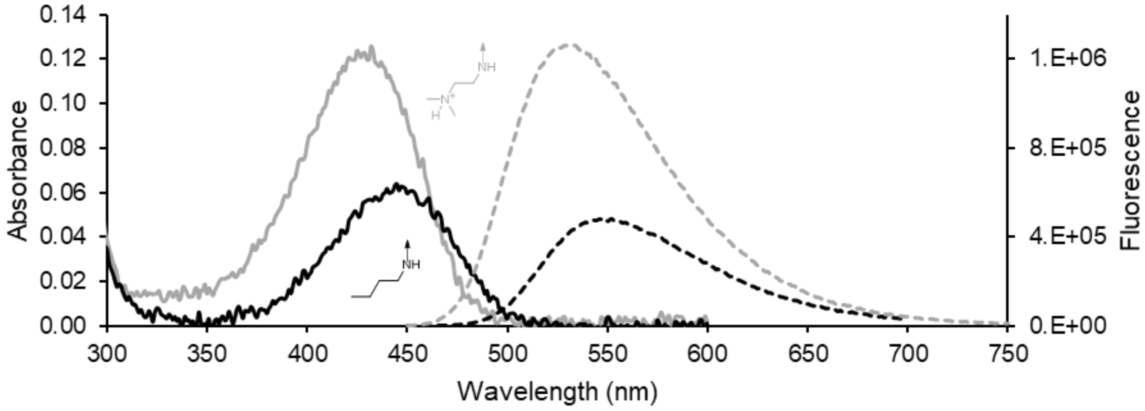

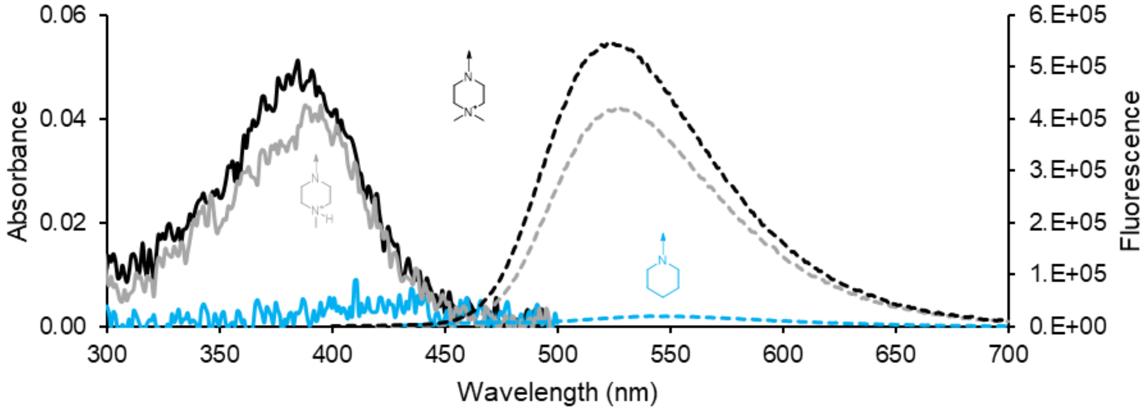

Series 2

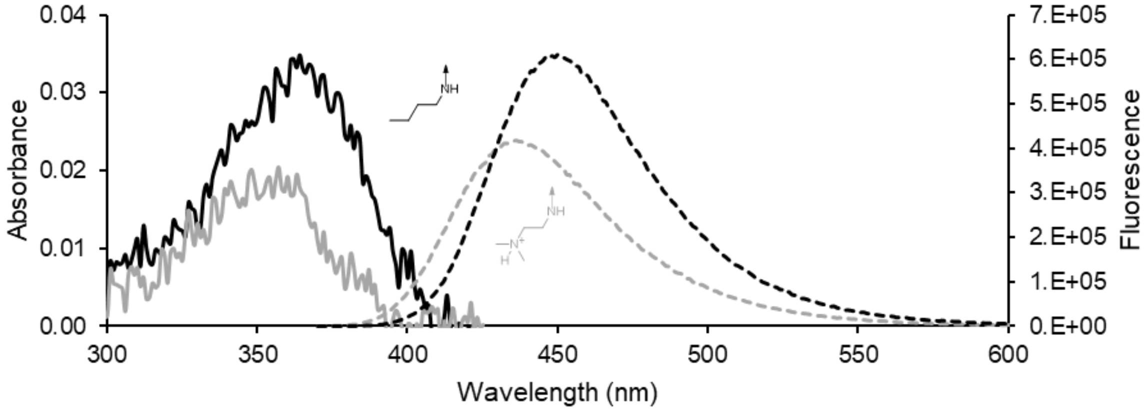

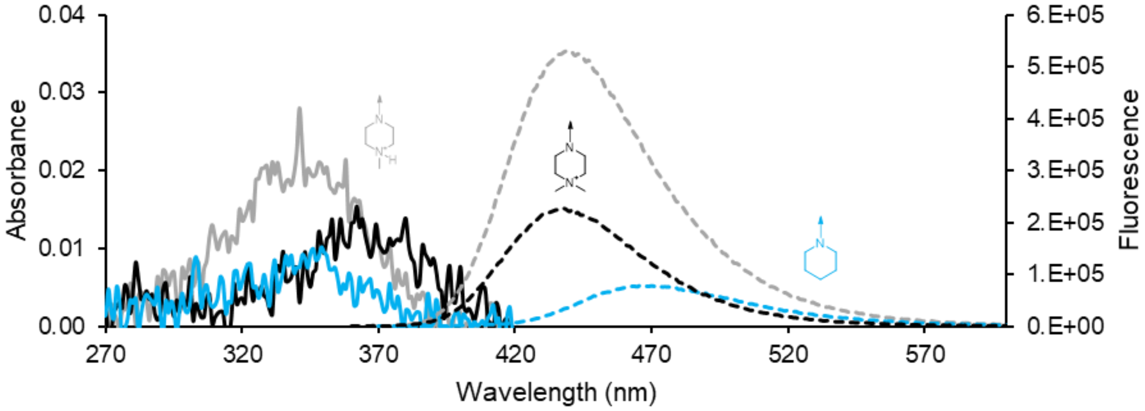

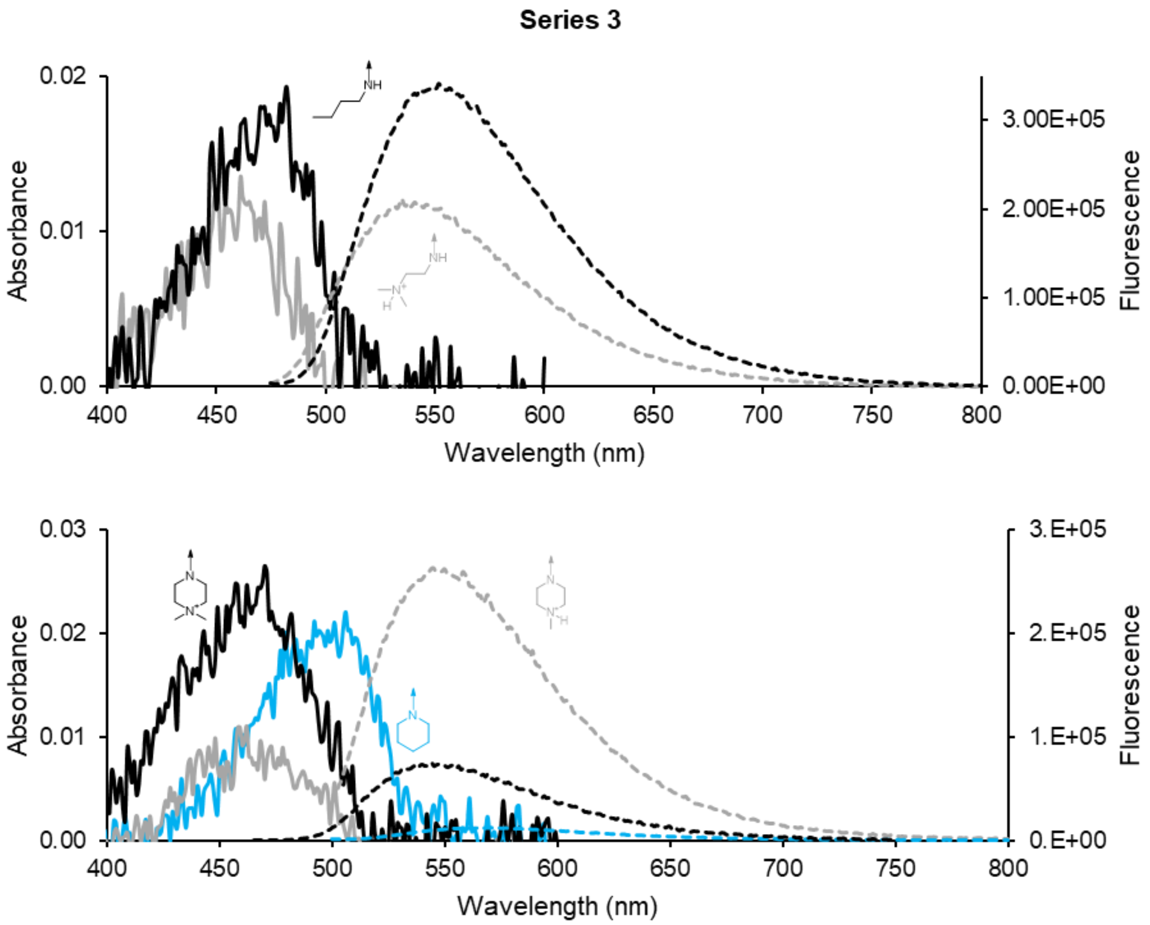

Figure S6 Effect of pH on fluorescence quenching pathways (this and preceding pages) Absorbances (solid lines) and fluorescences (dashed lines) in 1:1 MeCN/TRIS (10 mM) adjusted to the stated pH values. Series 1 top: 1B at pH 2 (grey) and 1A at pH 7(black); series 1 bottom: 1G at pH 2 (grey), 1F at pH 7 (black) and 1H at pH 7 (blue); all series 1 measurements made at 5 μM. Series 2 top: 2B at pH 2 (grey) and 2A at pH 7 (black); series 2 bottom: 2G at pH 2 (grey), 2F at pH 7 (black) and 2H at pH 7 (blue); all series 2 measurements made at 1 μM. Series 3 top: 3B at pH 2 (grey) and 3A at pH 7 (black); series 3 bottom: 3G at pH 2 (grey), 3F at pH 7 (blue) and 3H at pH 7 (black); all series 3 measurements made at 1 μM

Fluorescence variation with viscosity

Methanol/glycerol mixtures were prepared (10, 20, 30, 40, 50, 60, 70% w/w glycerol) and used to prepare solutions of **1A** – **3H** at the concentrations indicated in Table S1. The wavelengths giving maximum emission were determined using excitation scans (Table S2) and the compound solutions excited at these wavelengths. Normalised maximal emission (see Table S2 for wavelengths) was plotted against known viscosity of the the methanol/glycerol mixtures.[5]

	10	20	30	40	50	60	70
**1A**	446 (545)	449 (545)	449 (545)	449 (545)	449 (545)	449 (545)	449 (545)
**1B**	434 (540)	435 (540)	435 (540)	437 (540)	441 (540)	441 (540)	441 (540)
**1C**	444 (543)	444 (543)	444 (543)	444 (543)	444 (543)	444 (543)	444 (543)
**1D**	434 (542)	434 (542)	434 (542)	434 (542)	434 (542)	435 (542)	434 (542)
**1E**	450 (550)	450 (550)	450 (550)	453 (550)	453 (550)	456 (550)	456 (550)
**1F**	430 (549)	430 (549)	430 (549)	430 (549)	430 (549)	430 (549)	421 (549)
**1G**	425 (536)	420 (536)	420 (536)	430 (536)	420 (536)	417 (536)	417 (536)
**1H**	380 (520)	383 (520)	383 (520)	383 (520)	384 (520)	383 (520)	377 (520)
**2A**	368 (450)	367 (450)	368 (450)	369 (450)	370 (450)	370 (450)	370 (450)
**2B**	362 (440)	362 (440)	362 (440)	362 (440)	360 (440)	362 (440)	360 (440)
**2C**	375 (460)	377 (460)	377 (460)	377 (460)	379 (460)	380 (460)	379 (460)
**2D**	366 (451)	366 (451)	365 (451)	367 (451)	368 (451)	368 (451)	369 (451)
**2E**	365 (463)	366 (463)	364 (463)	368 (463)	365 (463)	368 (463)	369 (463)
**2F**	367 (469)	366 (469)	369 (469)	369 (469)	367 (469)	372 (469)	369 (469)
**2G**	361 (451)	353 (451)	354 (451)	351 (451)	353 (451)	351 (451)	361 (451)
**2H**	344 (437)	343 (437)	347 (437)	345 (437)	345 (437)	347 (437)	346 (437)
**3A**	467 (543)	467 (544)	467 (542)	473 (548)	473 (547)	473 (549)	473 (551)
**3B**	462 (536)	462 (536)	463 (536)	462 (535)	468 (542)	462 (533)	463 (537)
**3C**	484 (552)	487 (552)	490 (552)	490 (552)	490 (552)	490 (552)	490 (552)
**3D**	472 (544)	473 (544)	480 (544)	480 (544)	480 (544)	480 (544)	480 (544)
**3E**	484 (557)	491 (557)	491 (557)	491 (557)	492 (557)	492 (557)	492 (557)
**3F**	467 (548)	479 (556)	481 (558)	486 (562)	487 (569)	-	486 (557)
**3G**	473 (551)	473 (550)	475 (551)	476 (552)	473 (550)	-	473 (548)
**3H**	450 (543)	450 (543)	456 (543)	456 (543)	457 (543)	461 (543)	461 (543)

Table S2 Excitation and (emission) wavelengths (nm) for all compounds in mixtures of varying percentage glycerol.

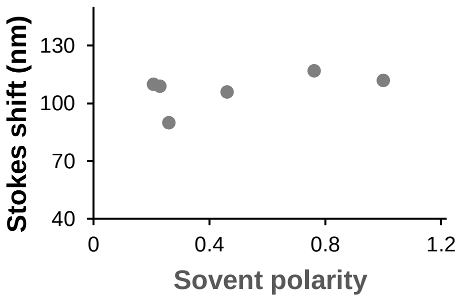

Figure S7 Stokes shift of compound 1C does not vary with solvent polarity (measured using the $E_T(30)$ scale).

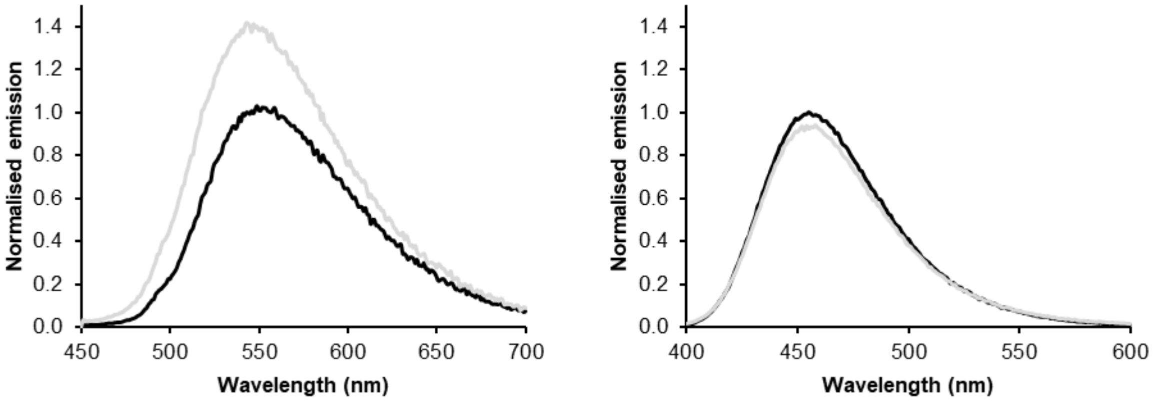

Figure S8 Effect of 0.2% w/v Carbopol on fluorescence Normalised emission of aqueous solution (black line) and 0.2% w/v Carbopol solution (grey line) of **1D** (5 μM, λ_{ex} = 422 nm, left) and **2D** (1 μM, λ_{ex} = 370 nm, right).

NMR data for **1A**

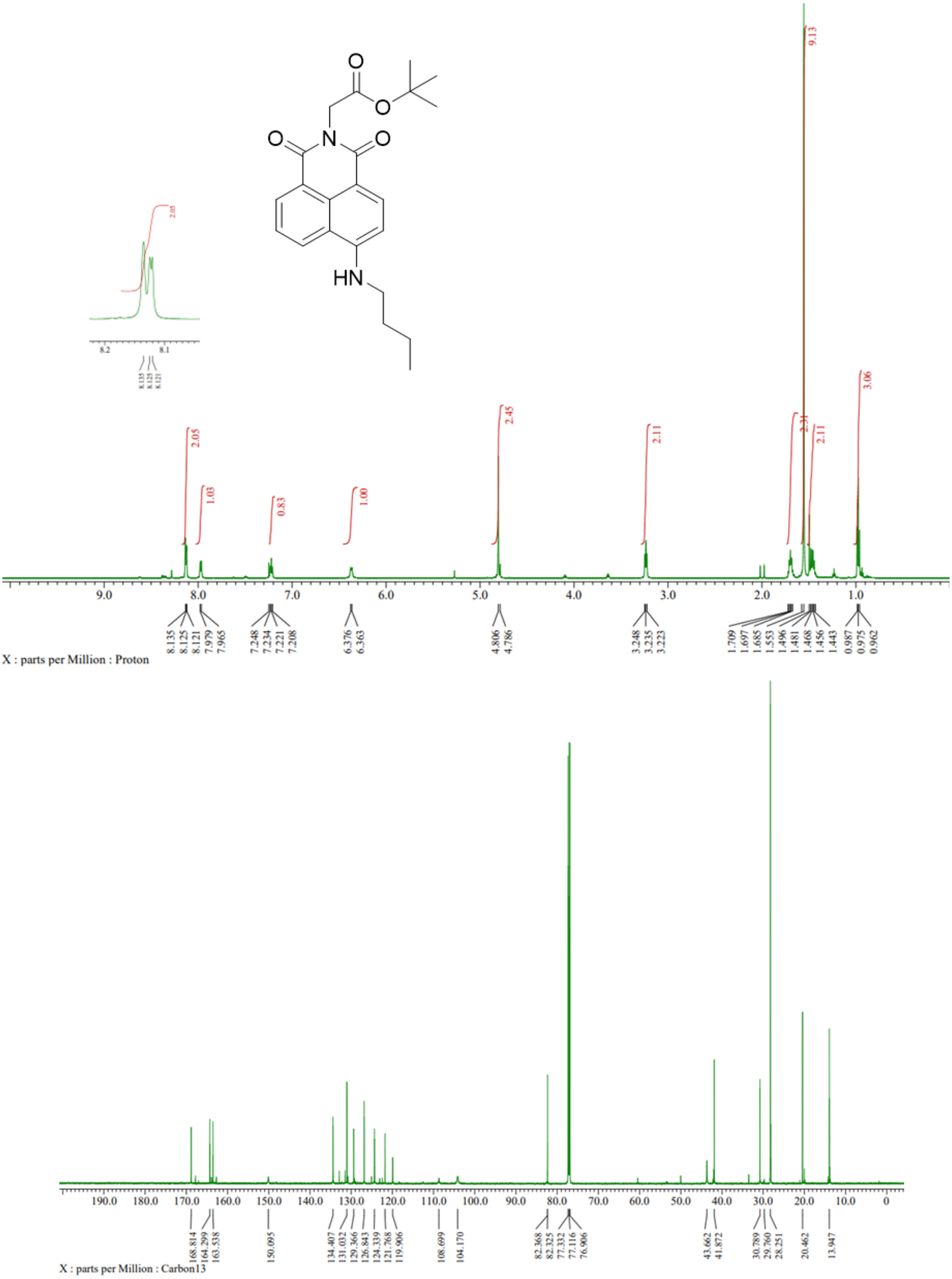

NMR data for **1B**

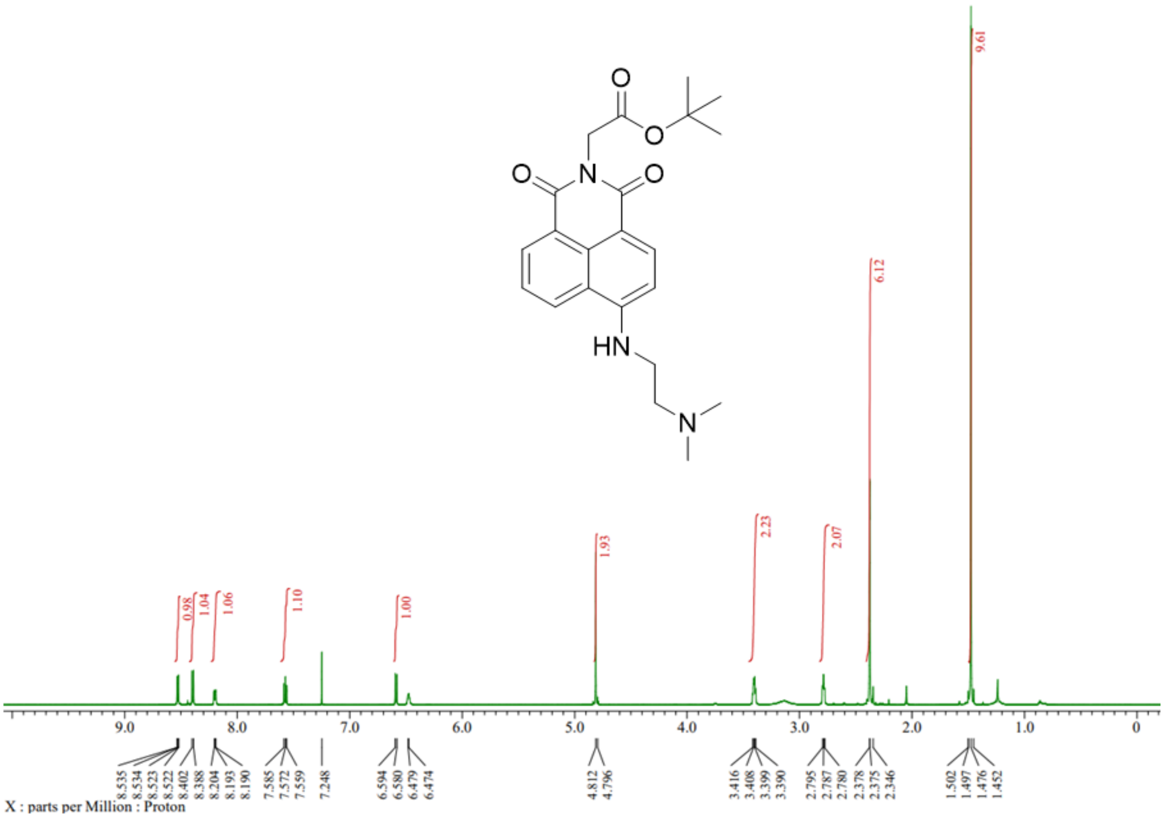

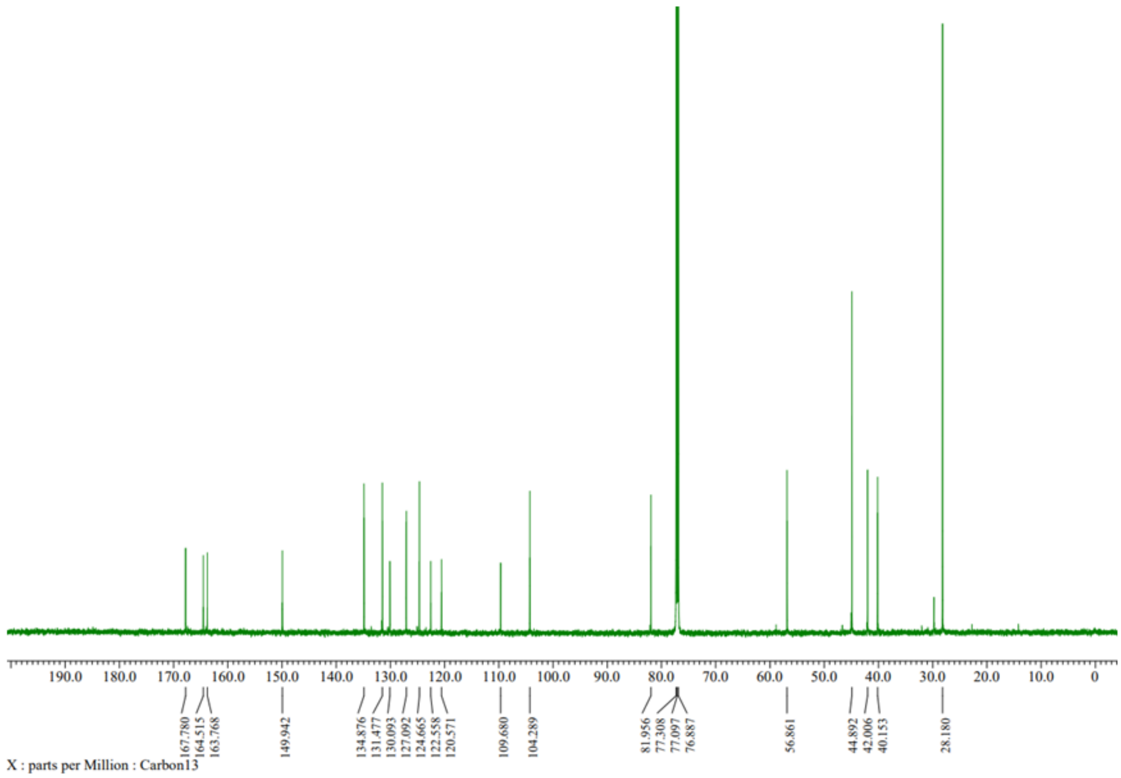

NMR data for **1C**

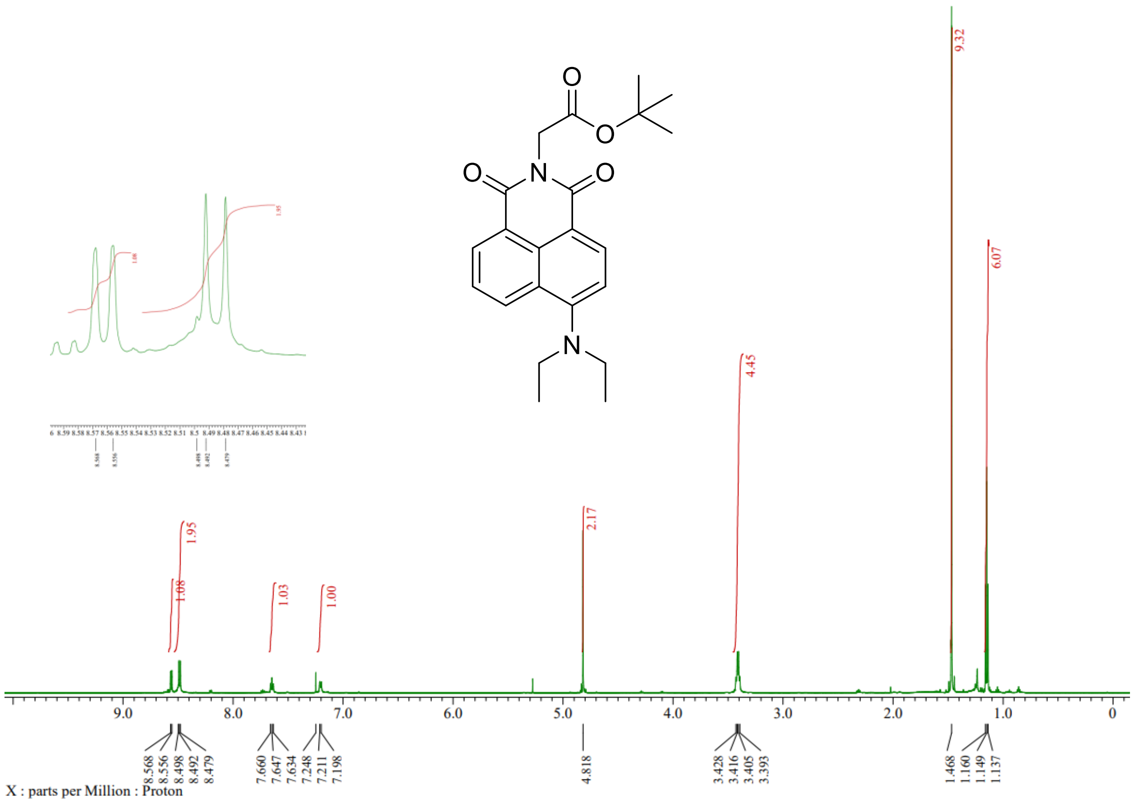

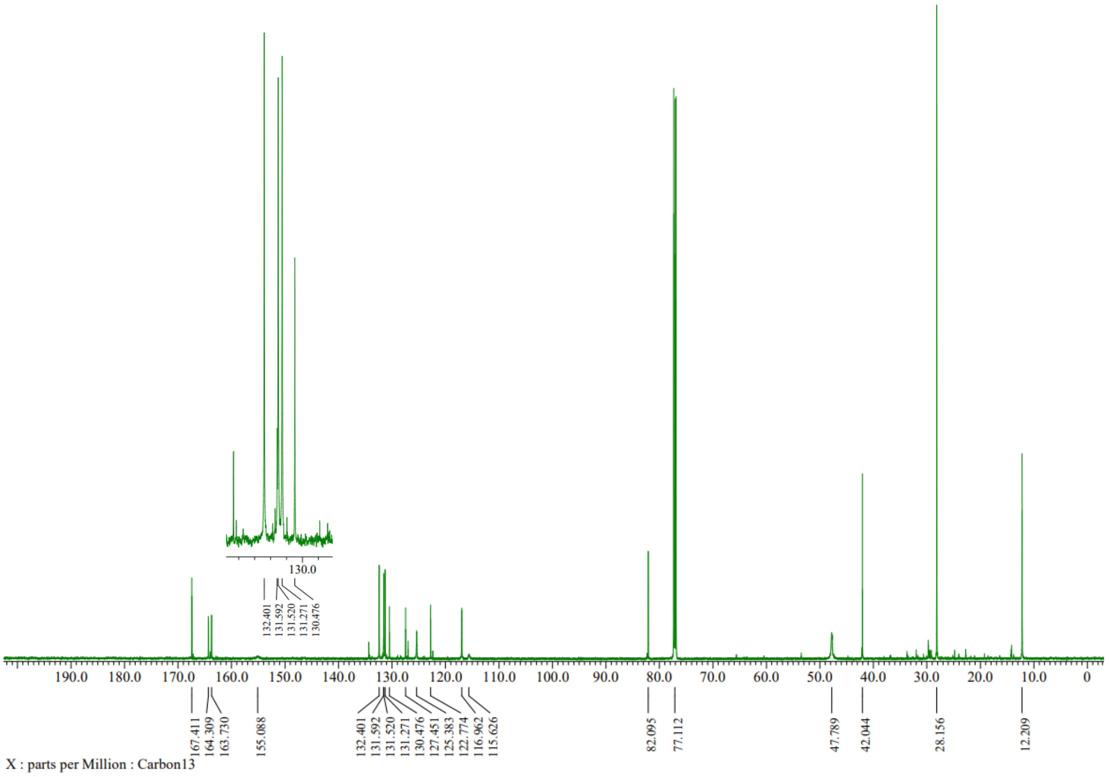

NMR data for **1D**

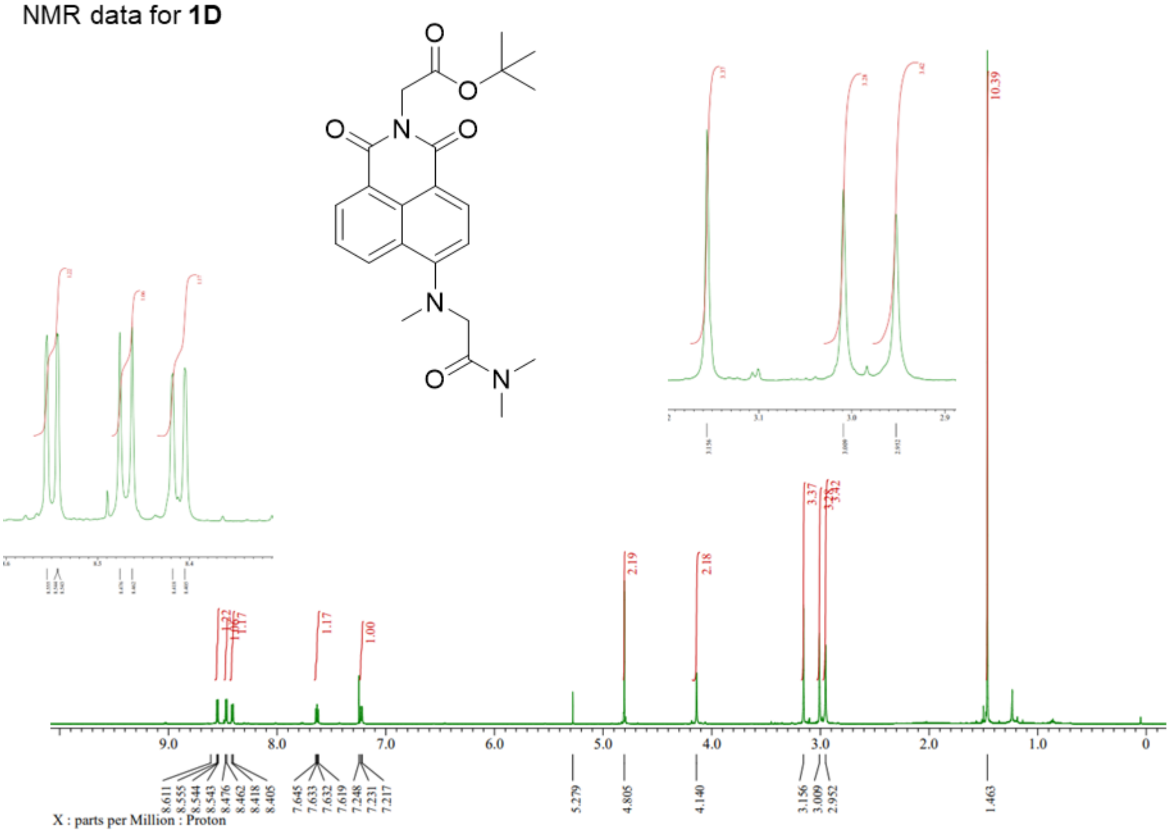

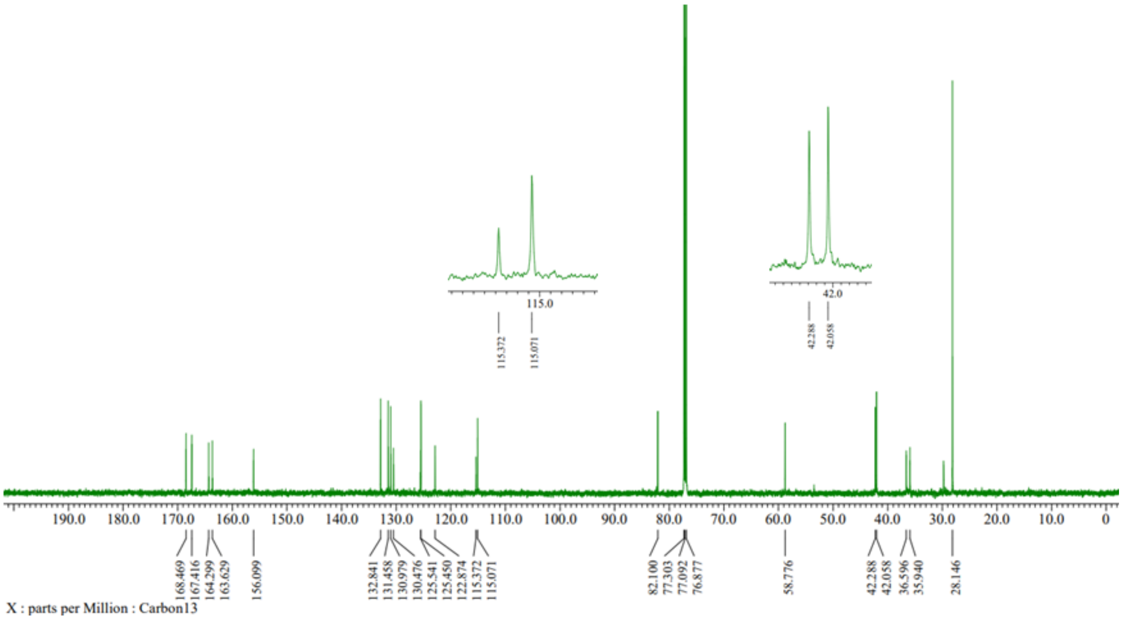

NMR data for **1E**

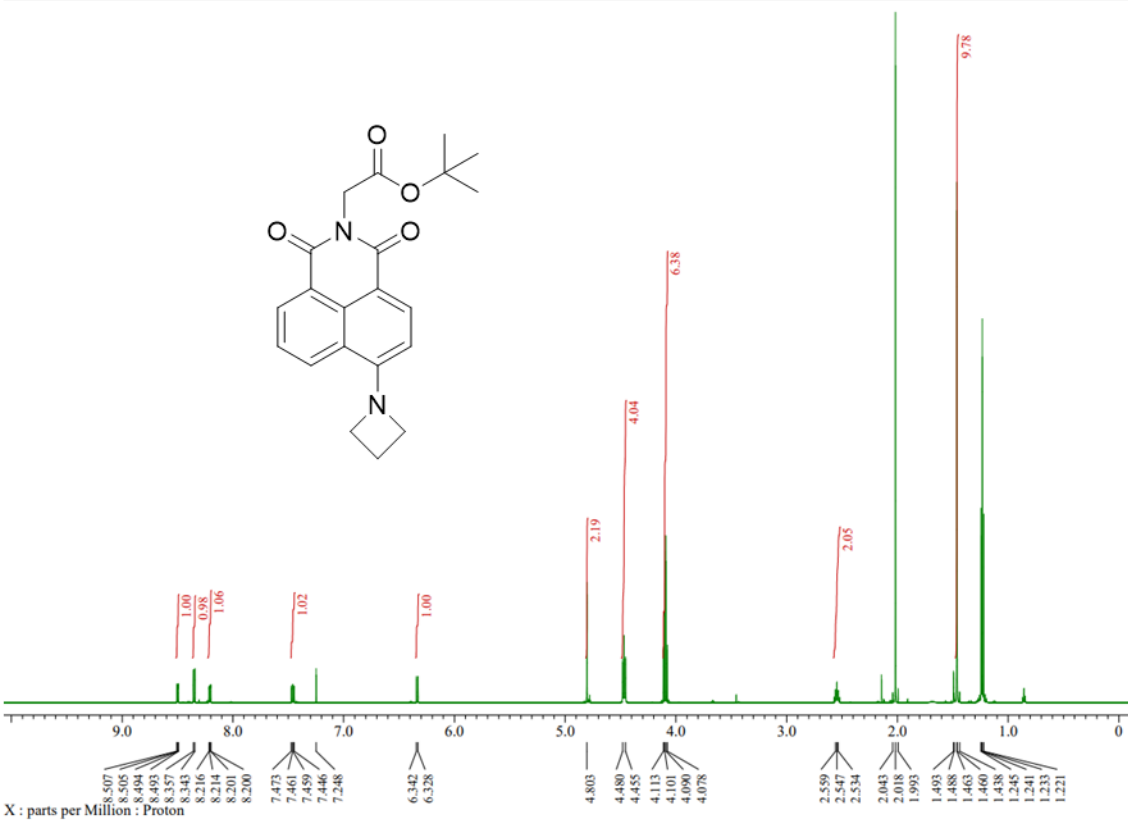

^{13}C not shown

NMR data for **1F**

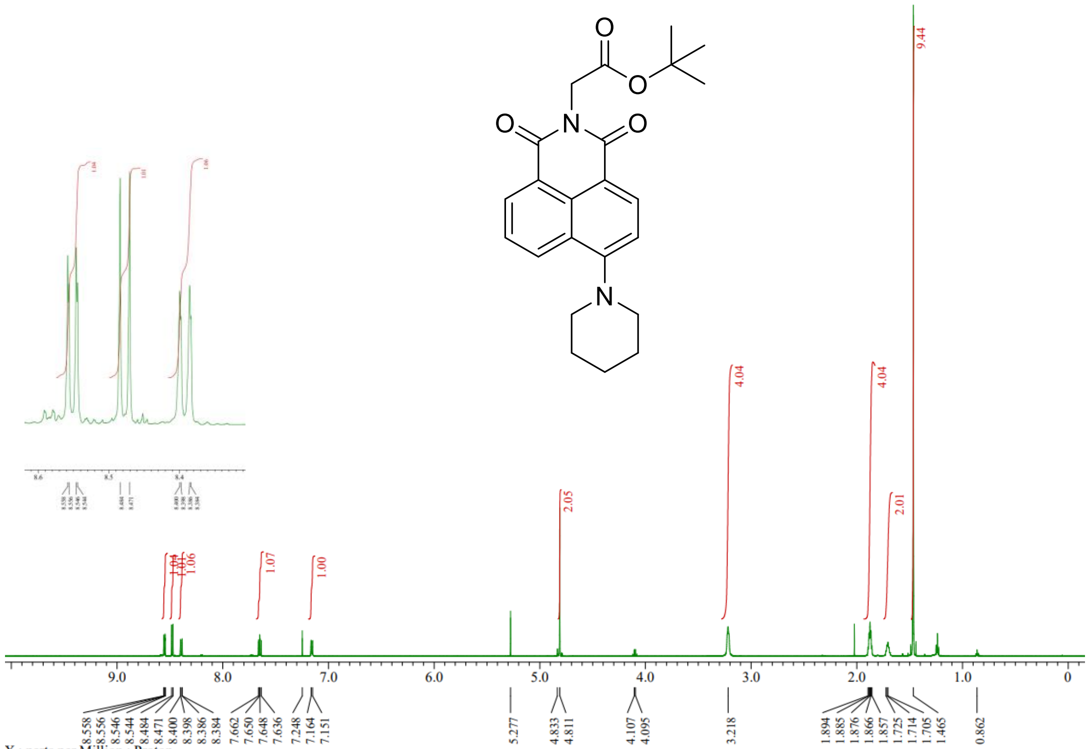

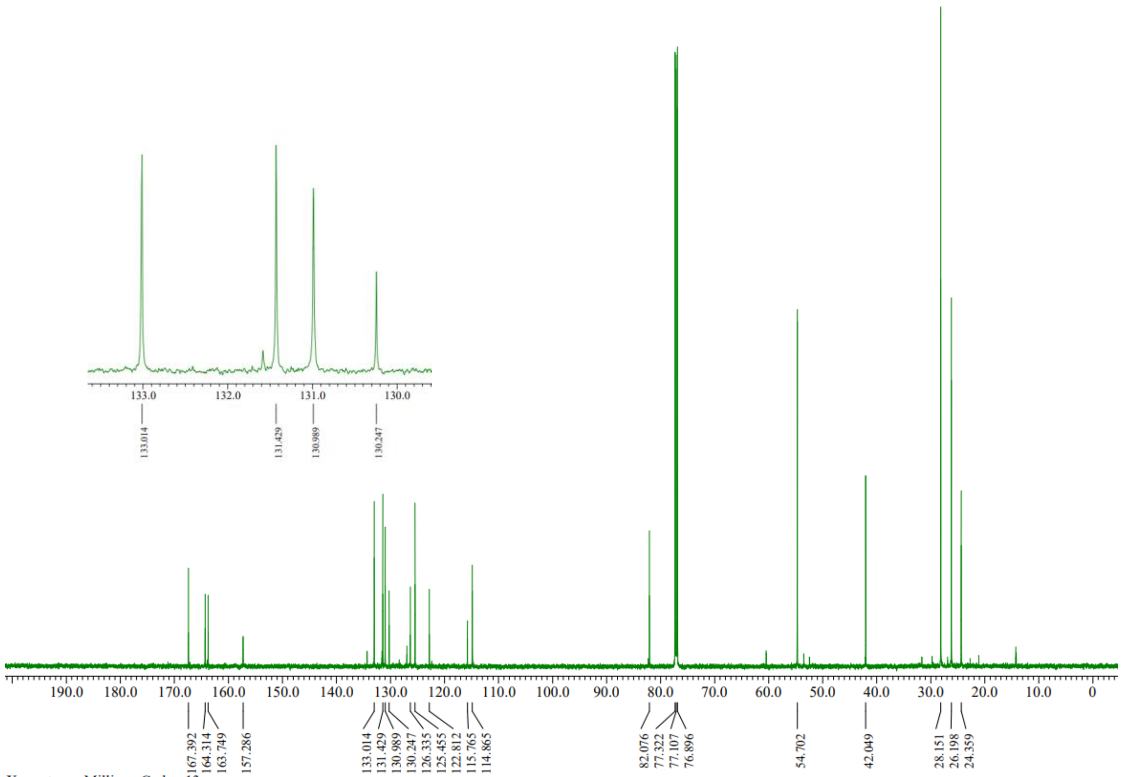

NMR data for **1G**

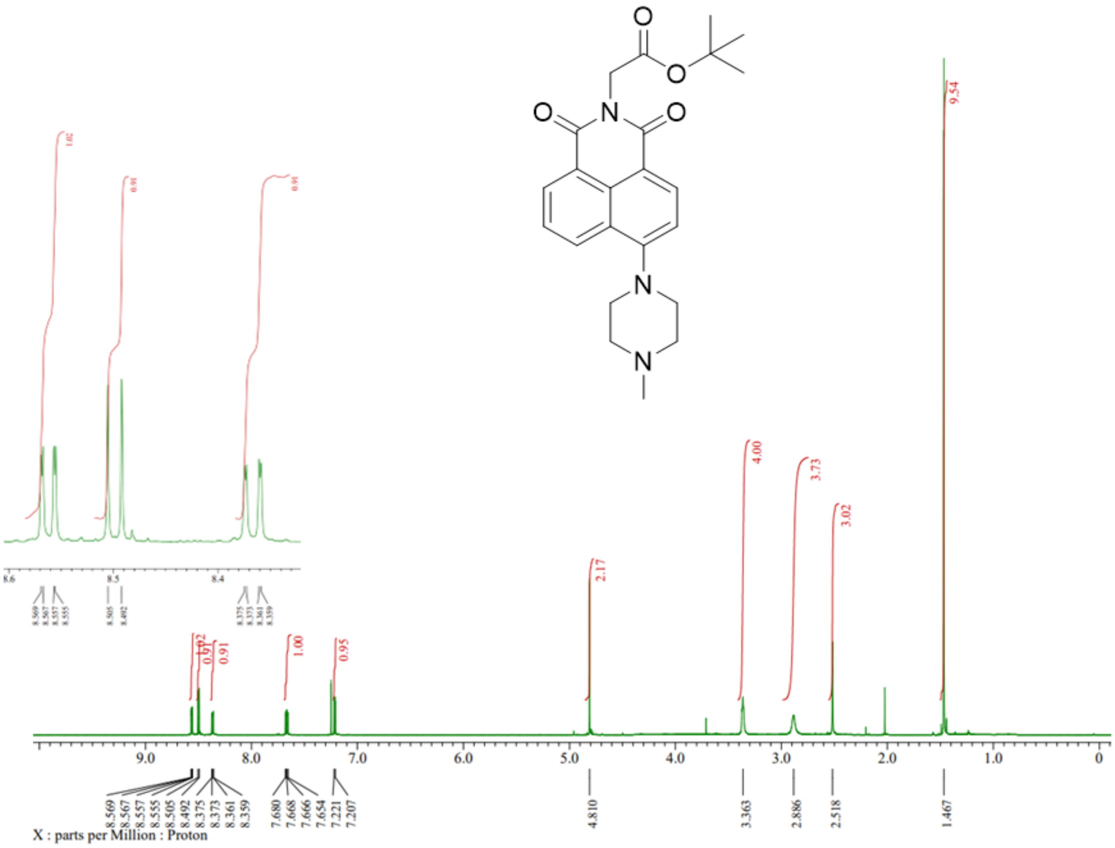

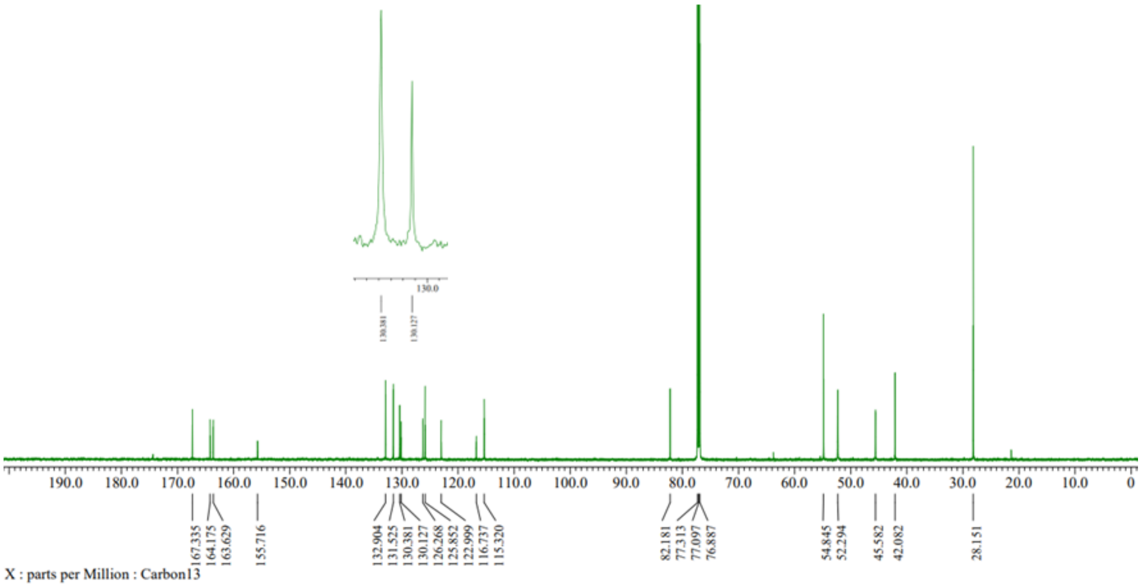

NMR data for **1H**

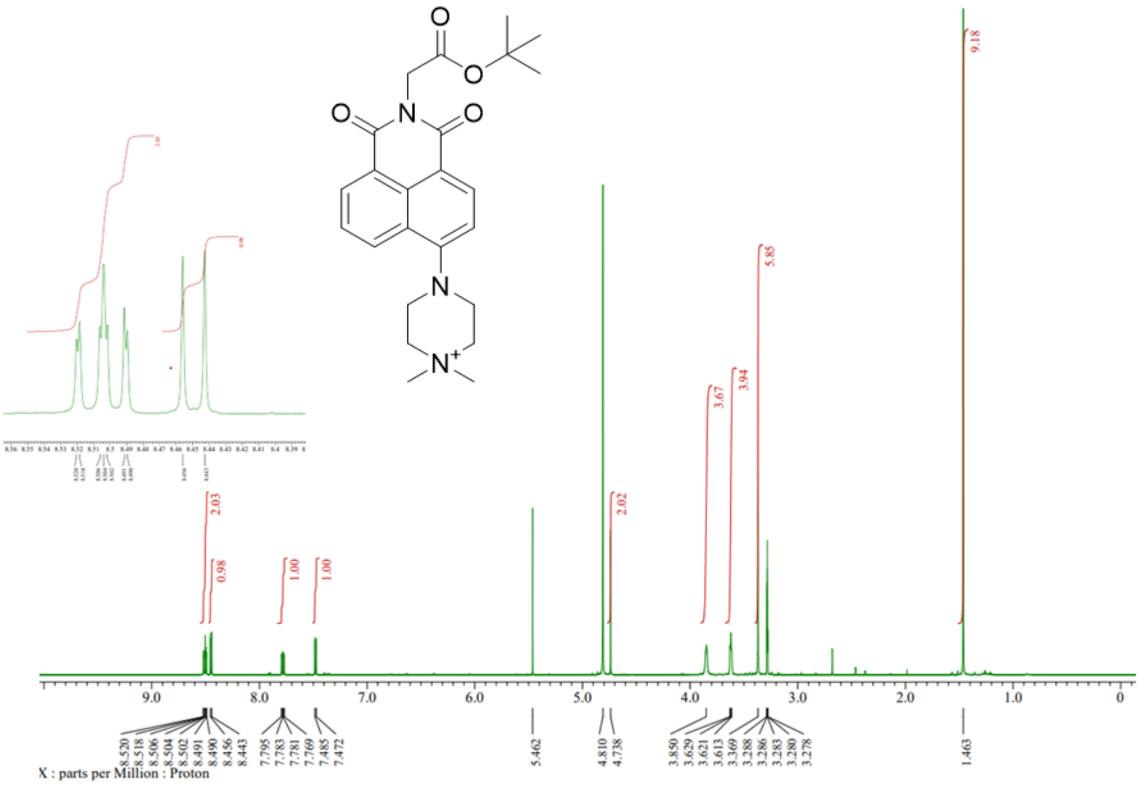

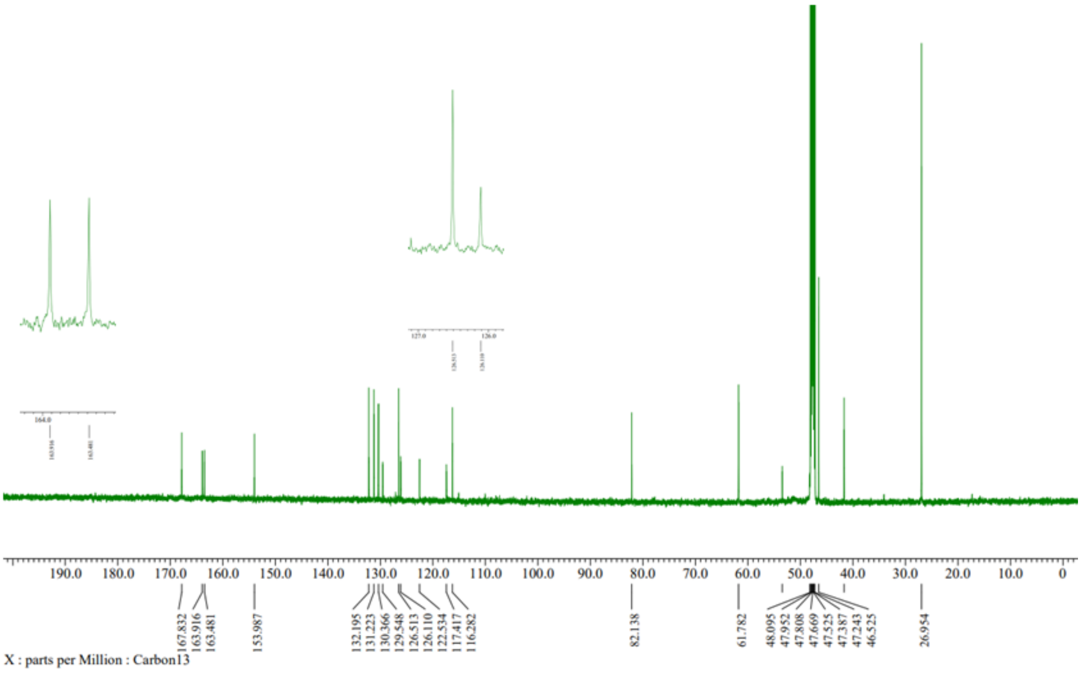

NMR data for **2A**

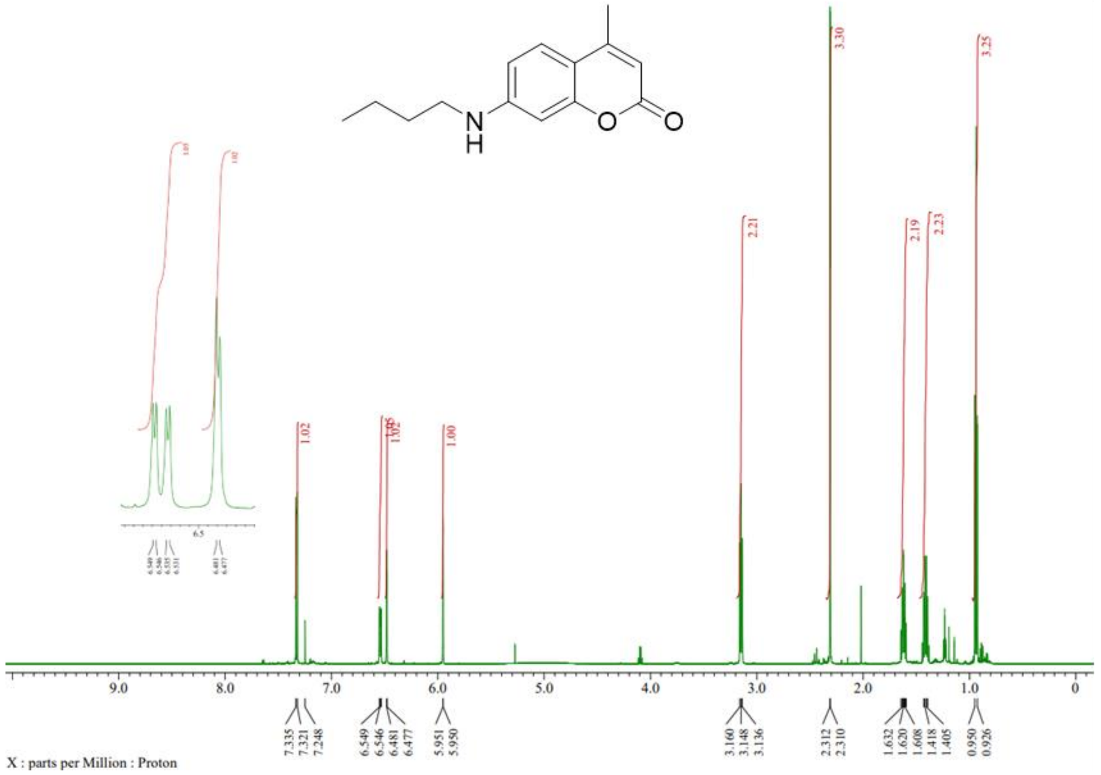

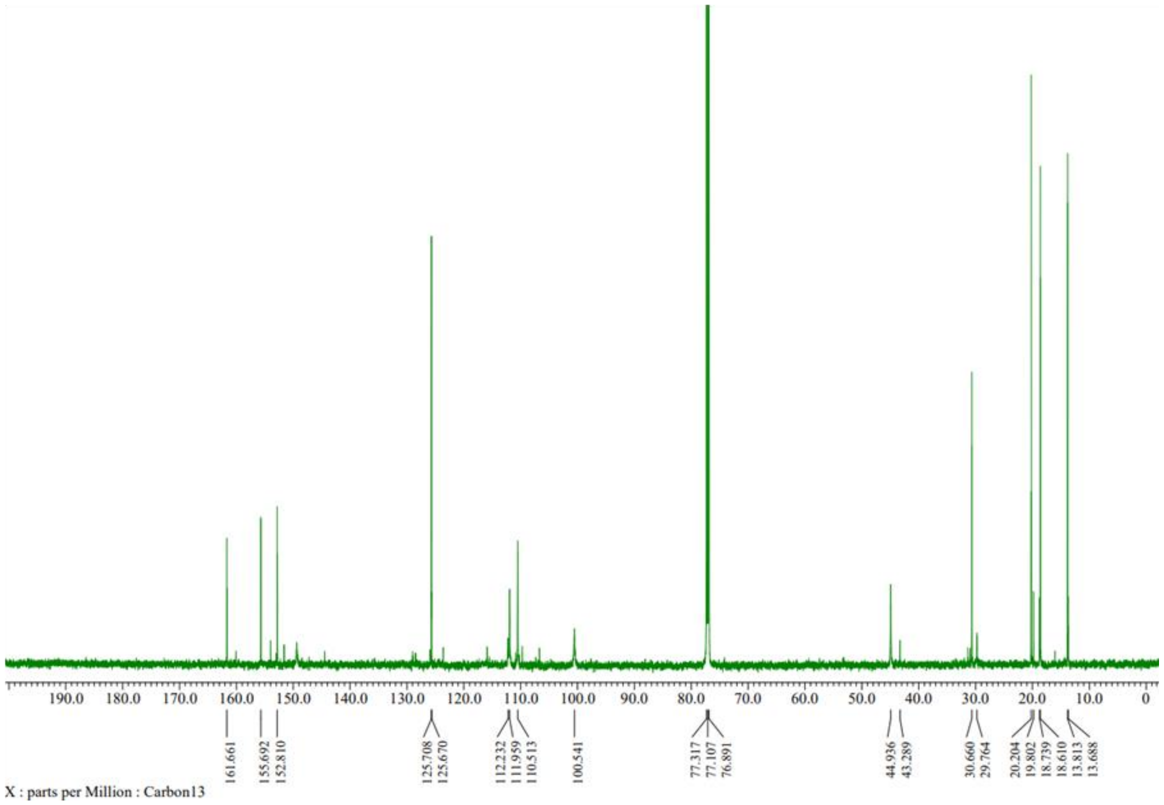

NMR data for **2B**

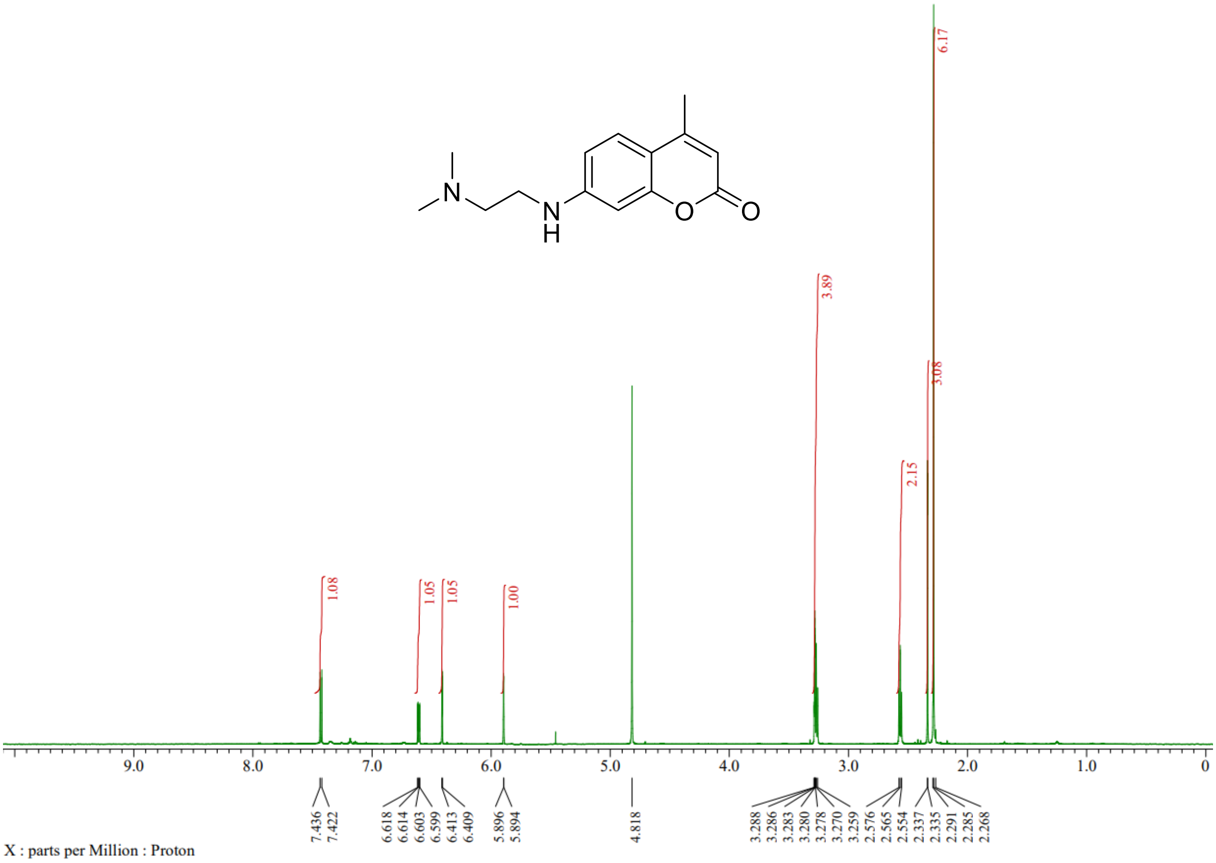

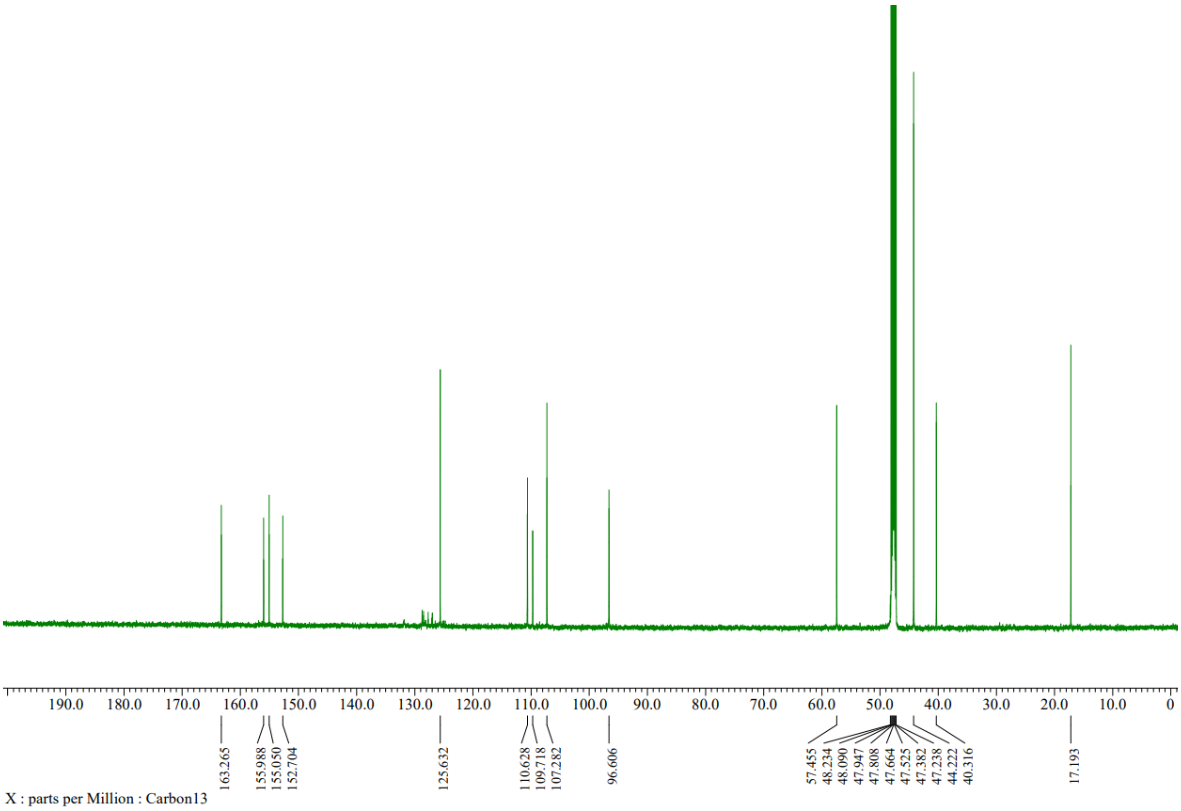

NMR data for **2C**

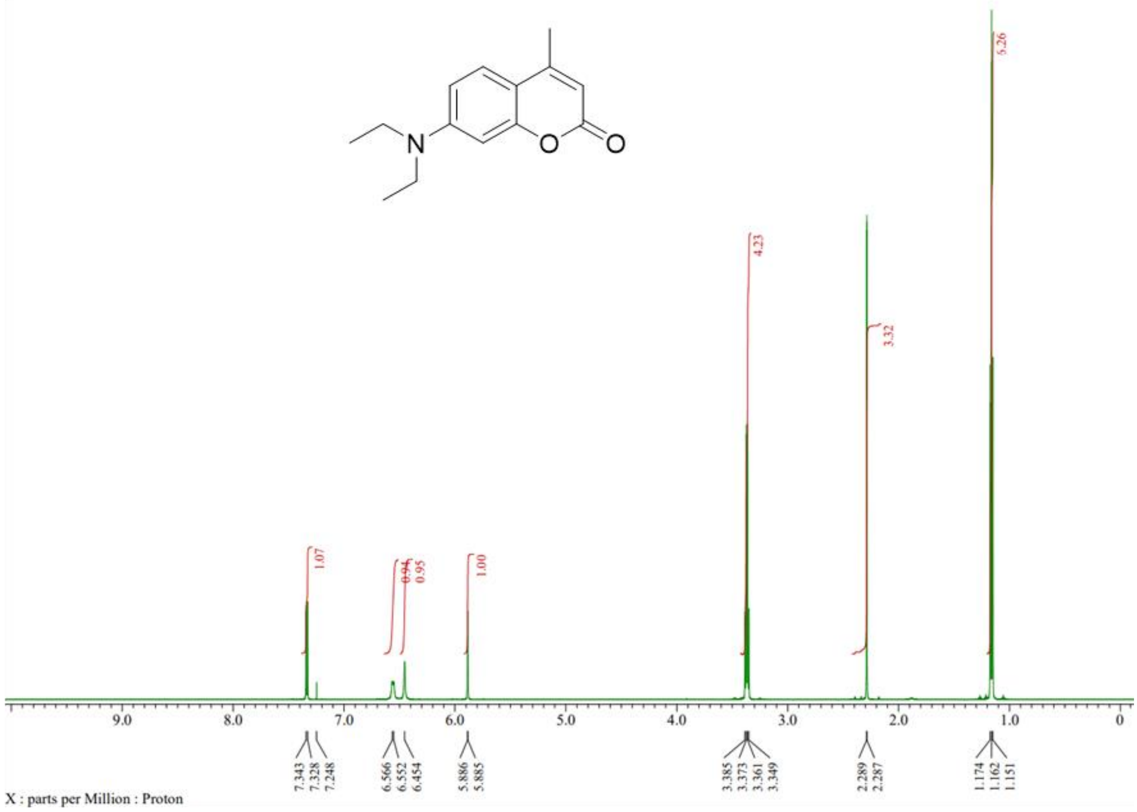

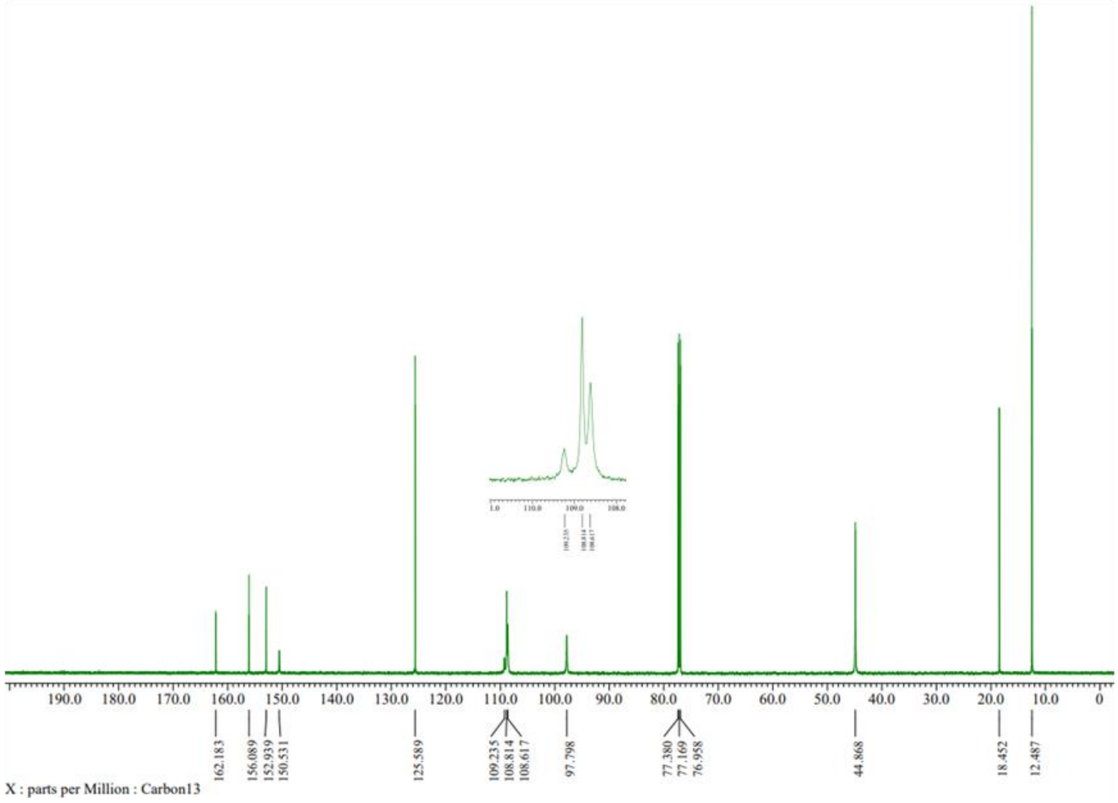

NMR data for **2D**

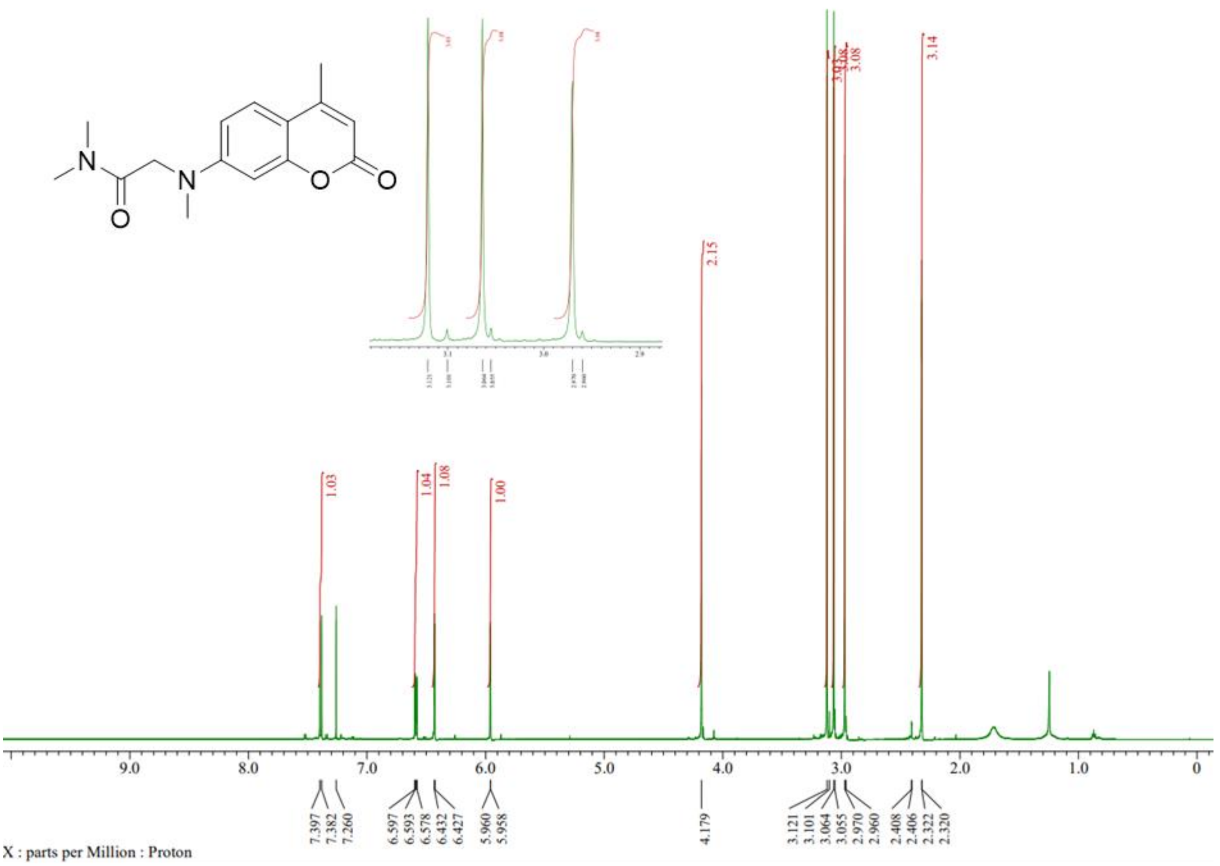

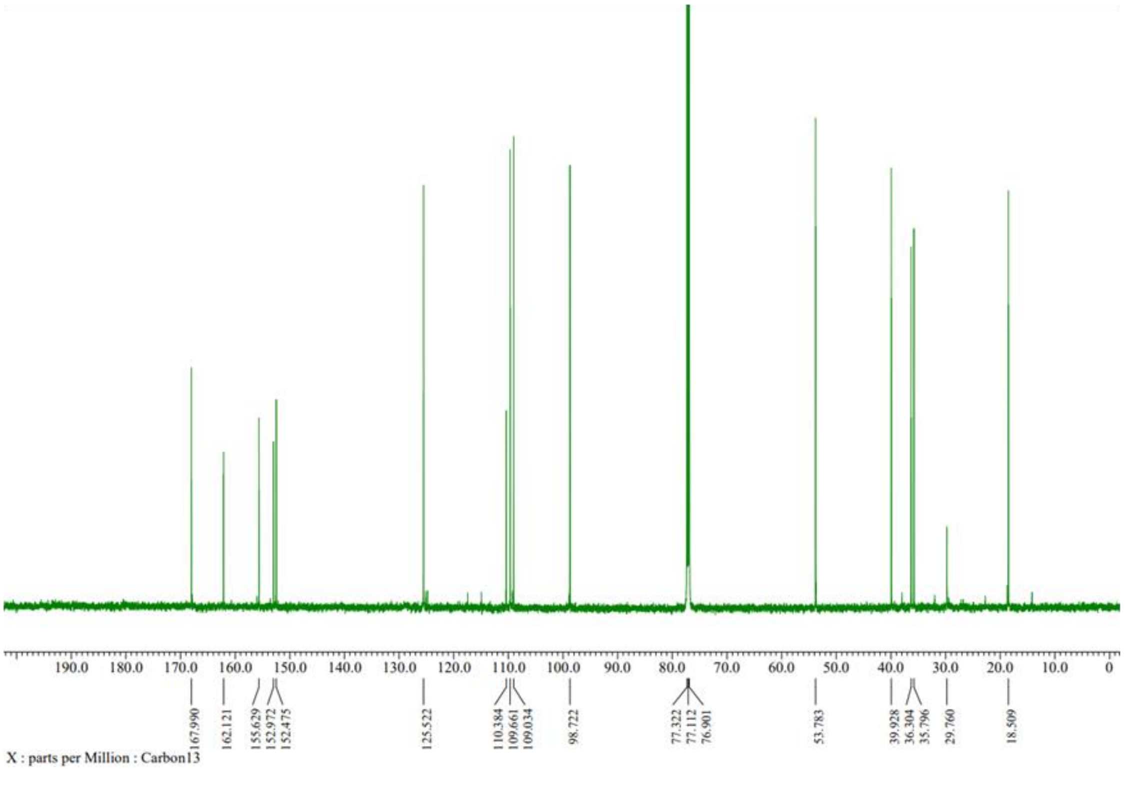

NMR data for **2E**

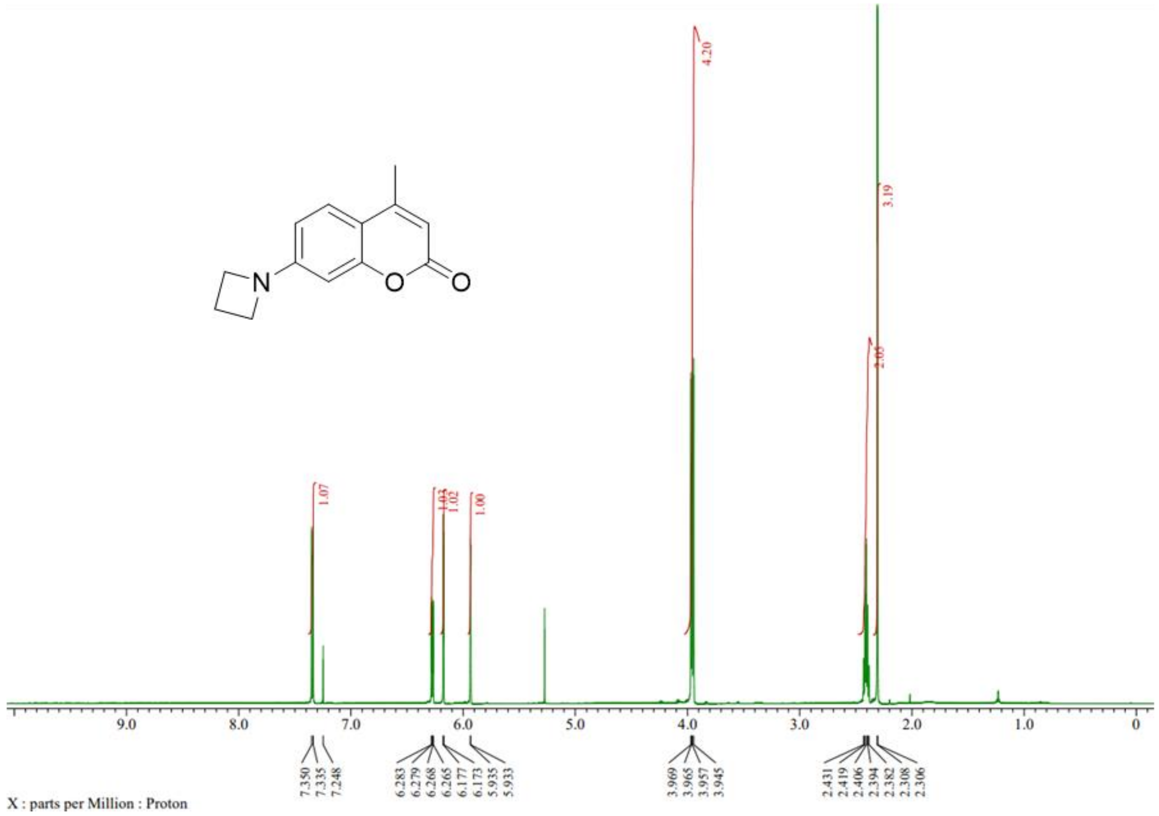

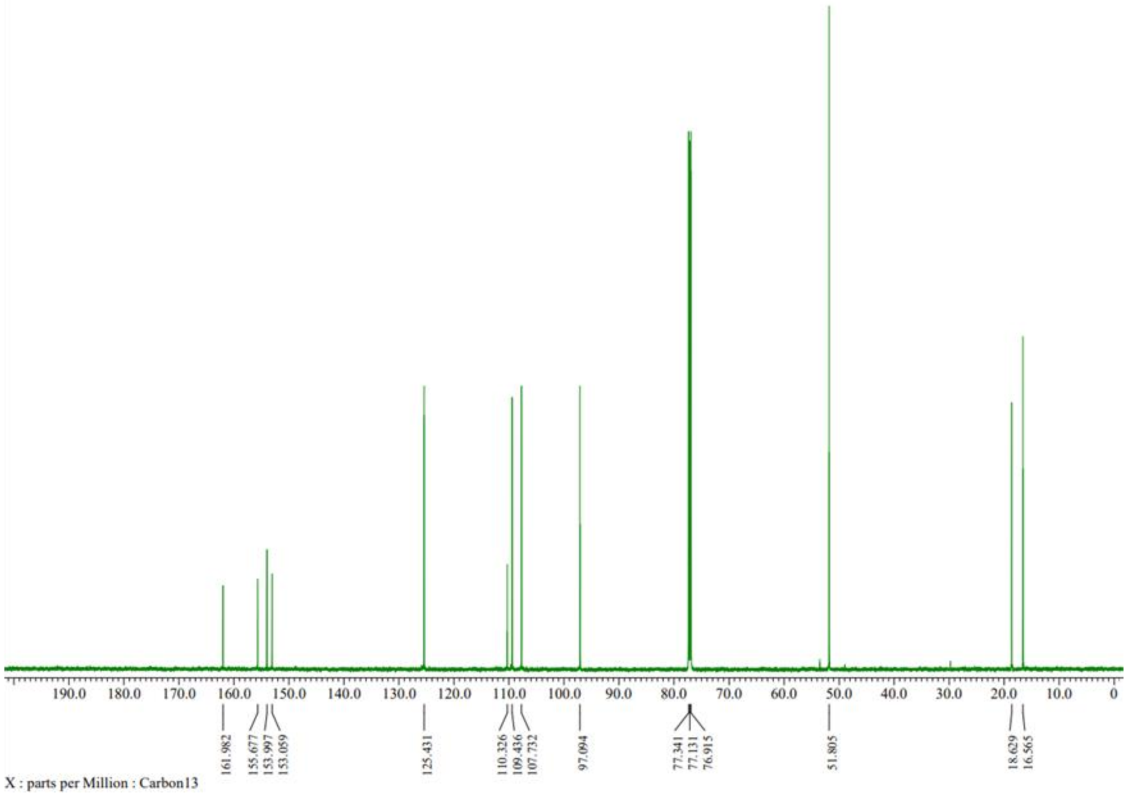

NMR data for **2F**

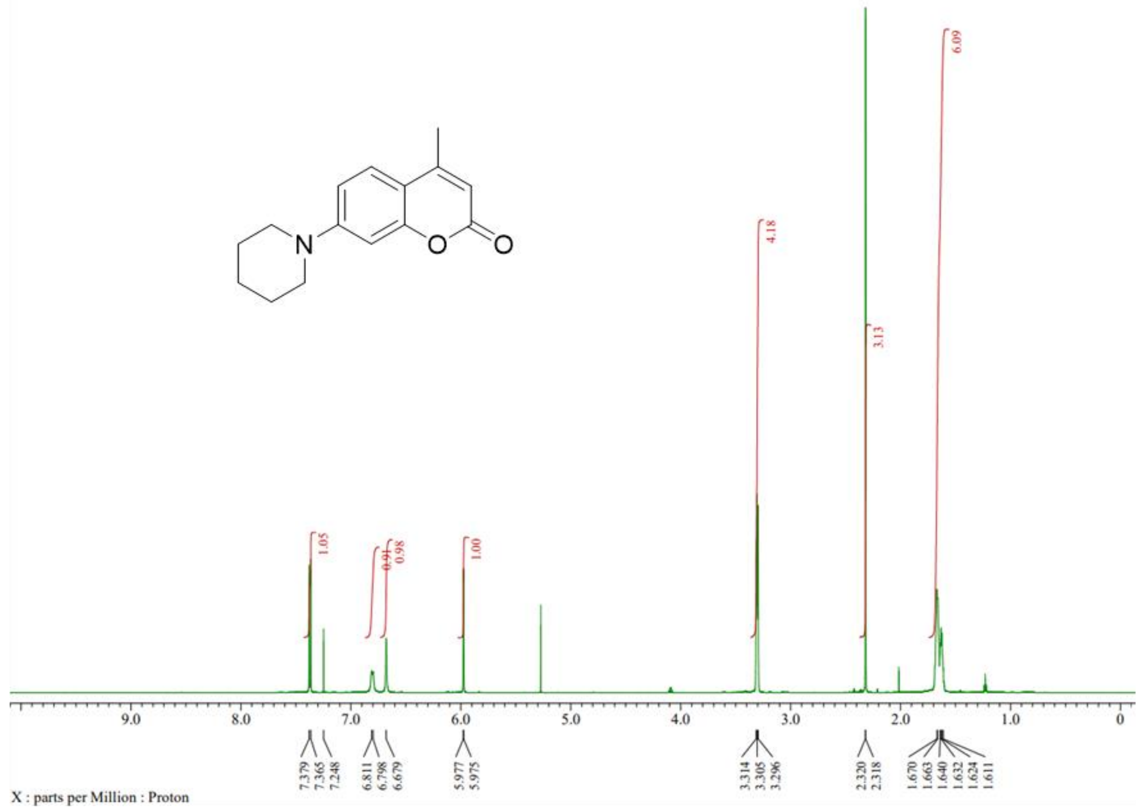

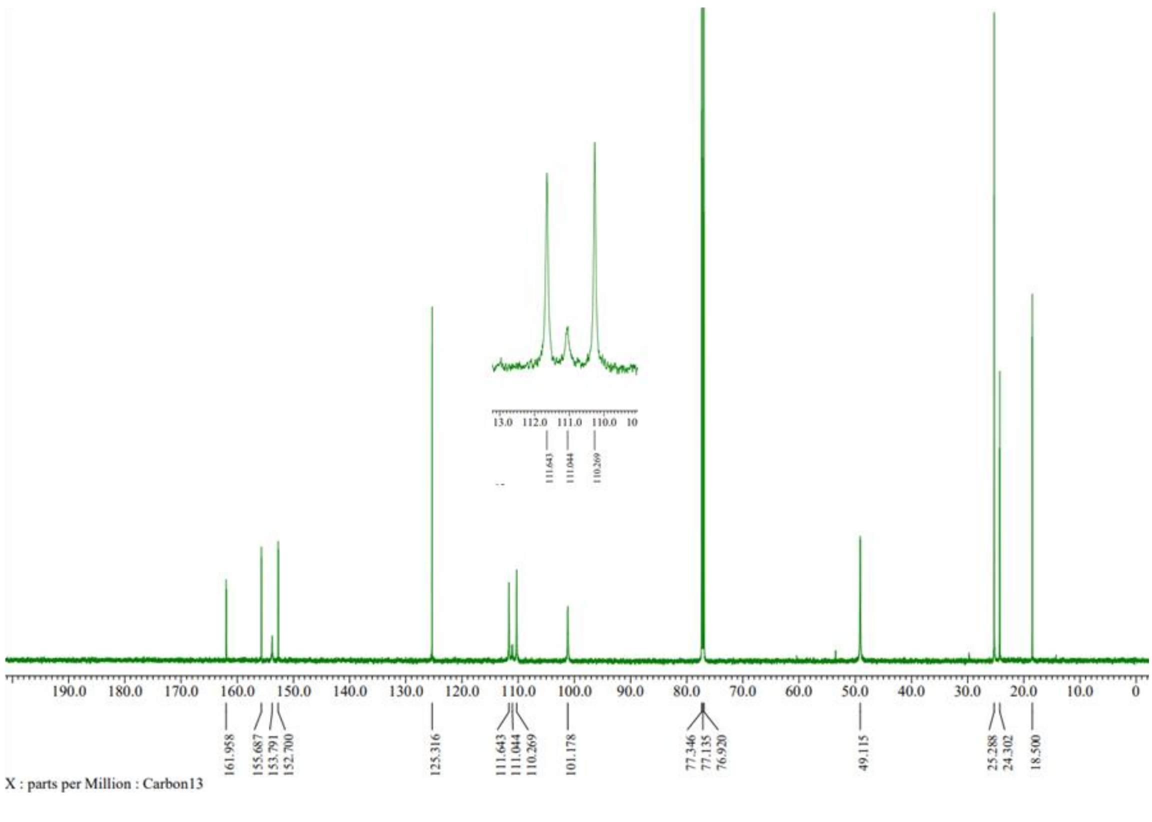

NMR data for **2G**

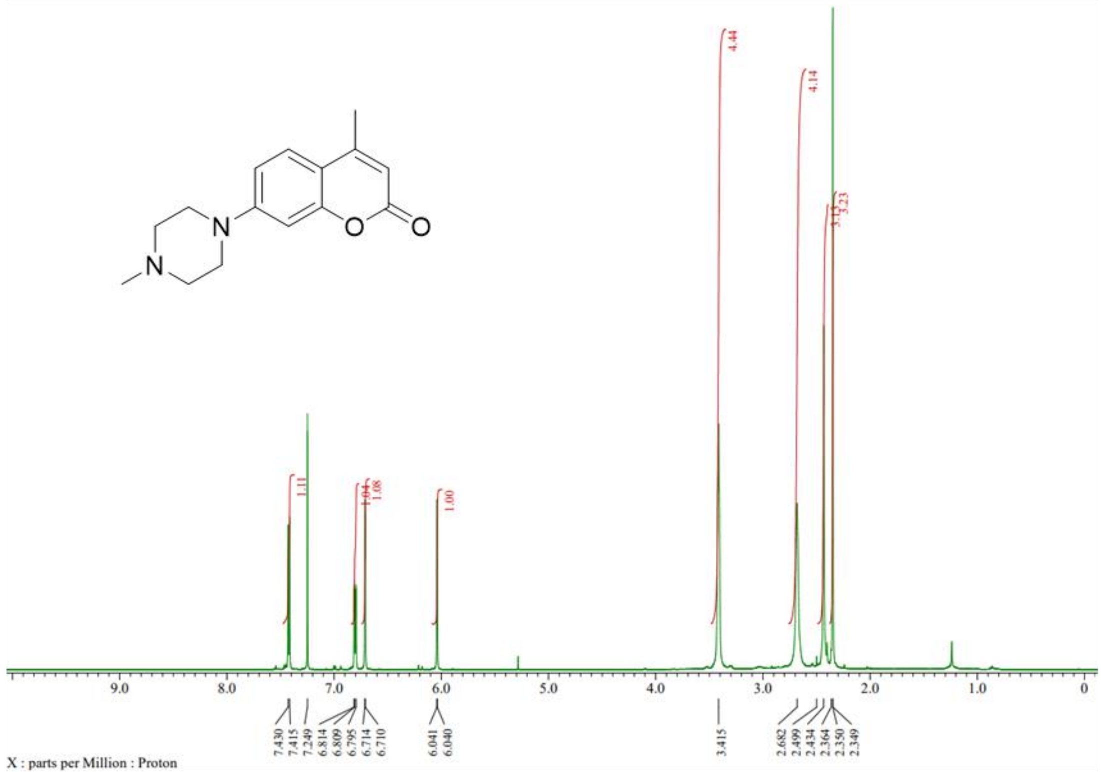

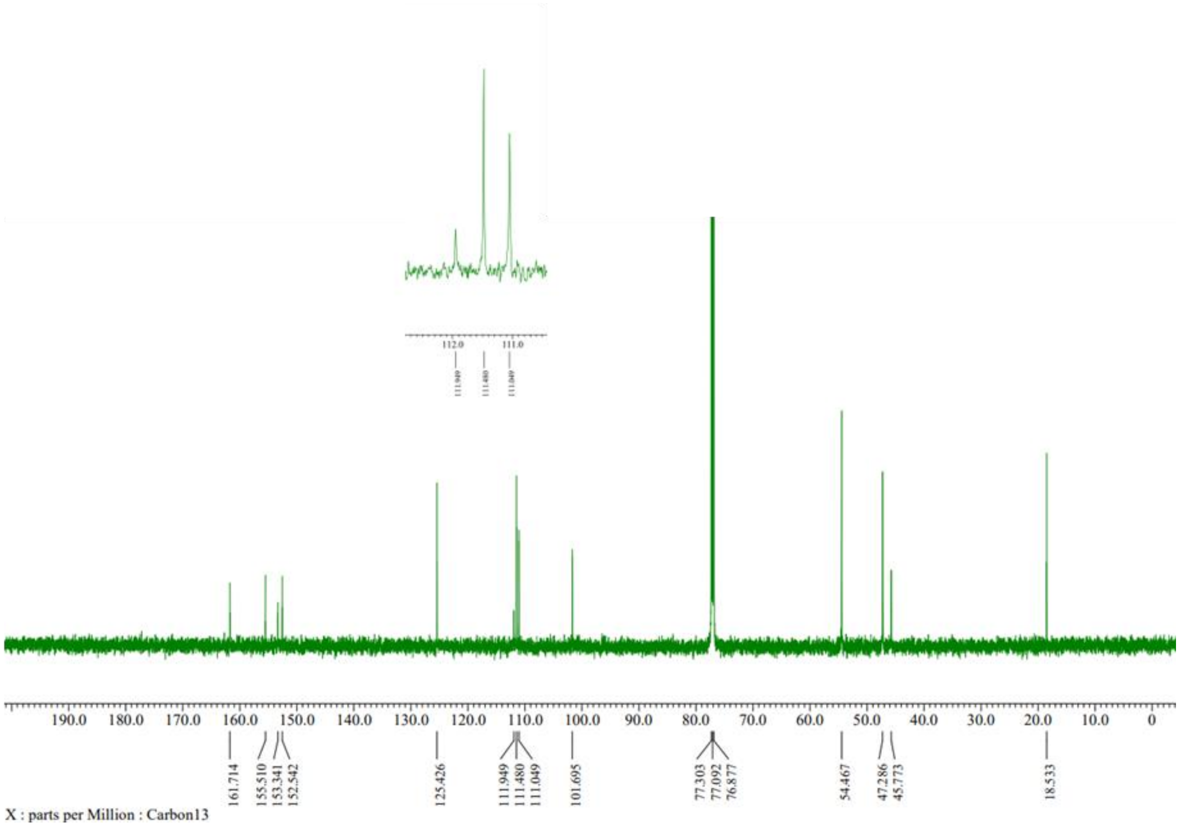

NMR data for **2H**

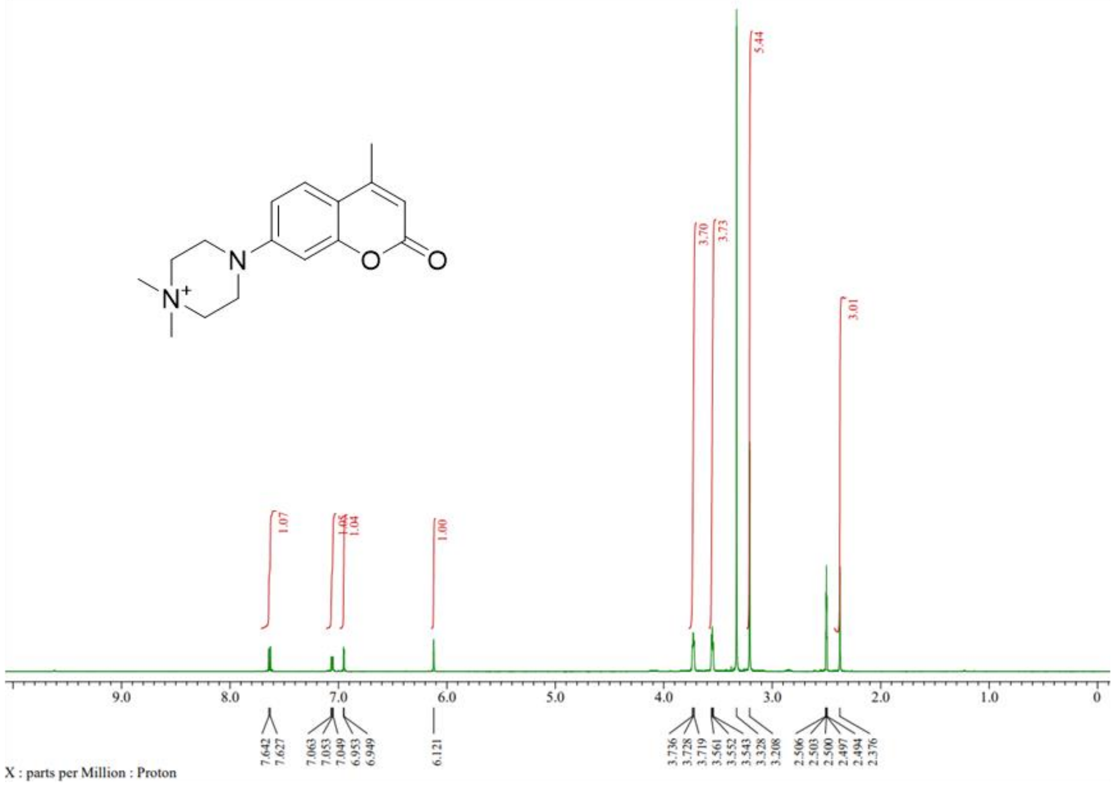

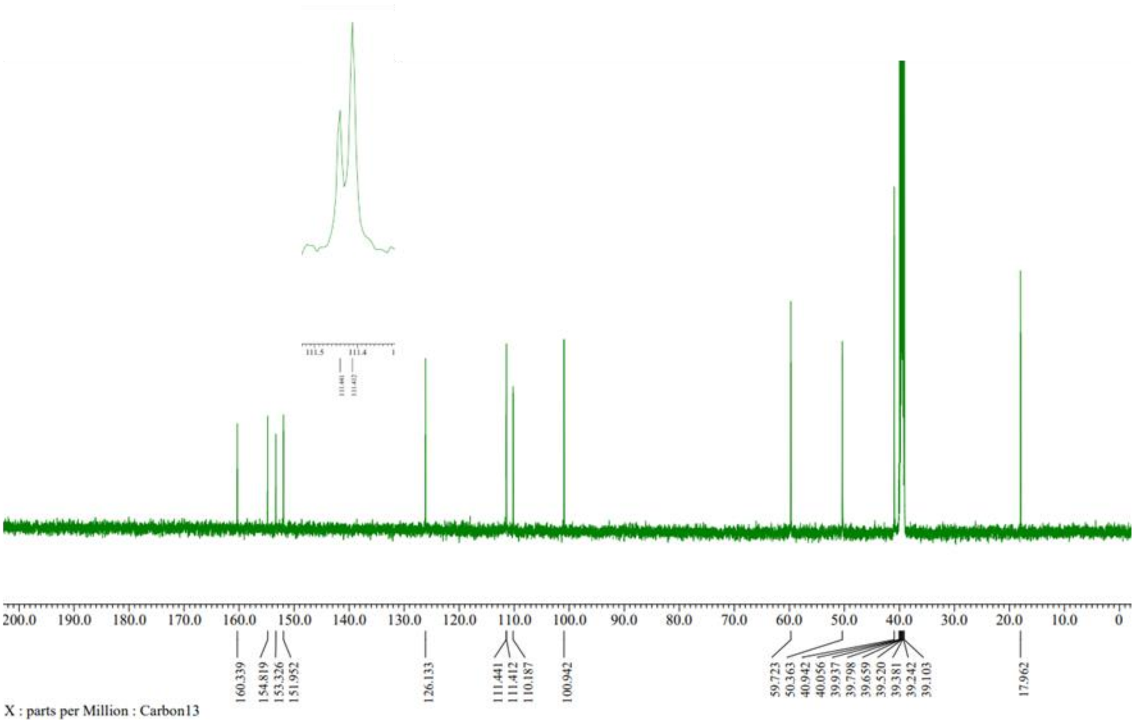

NMR data for **3A**

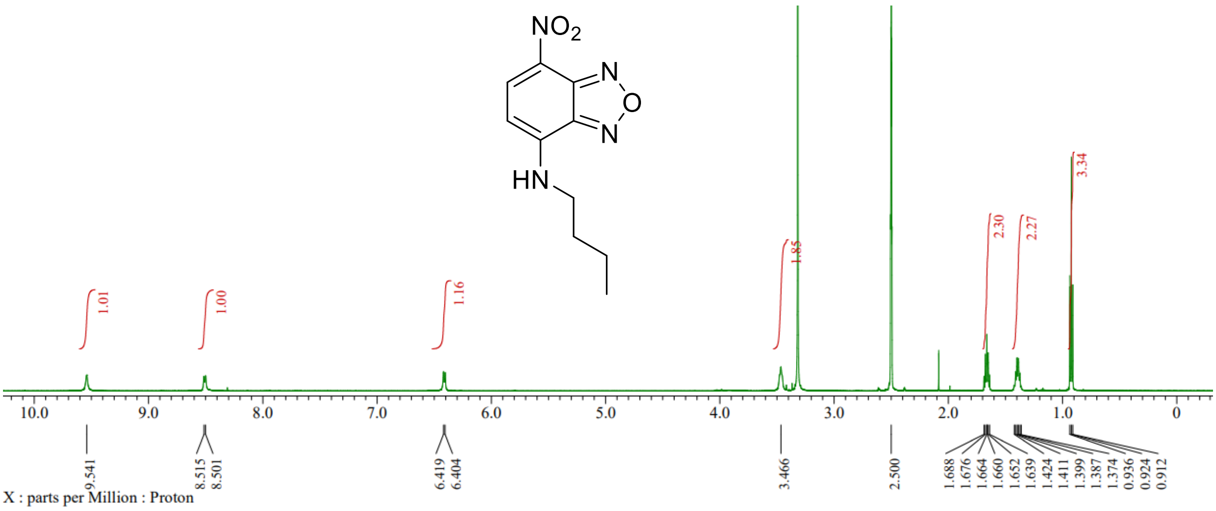

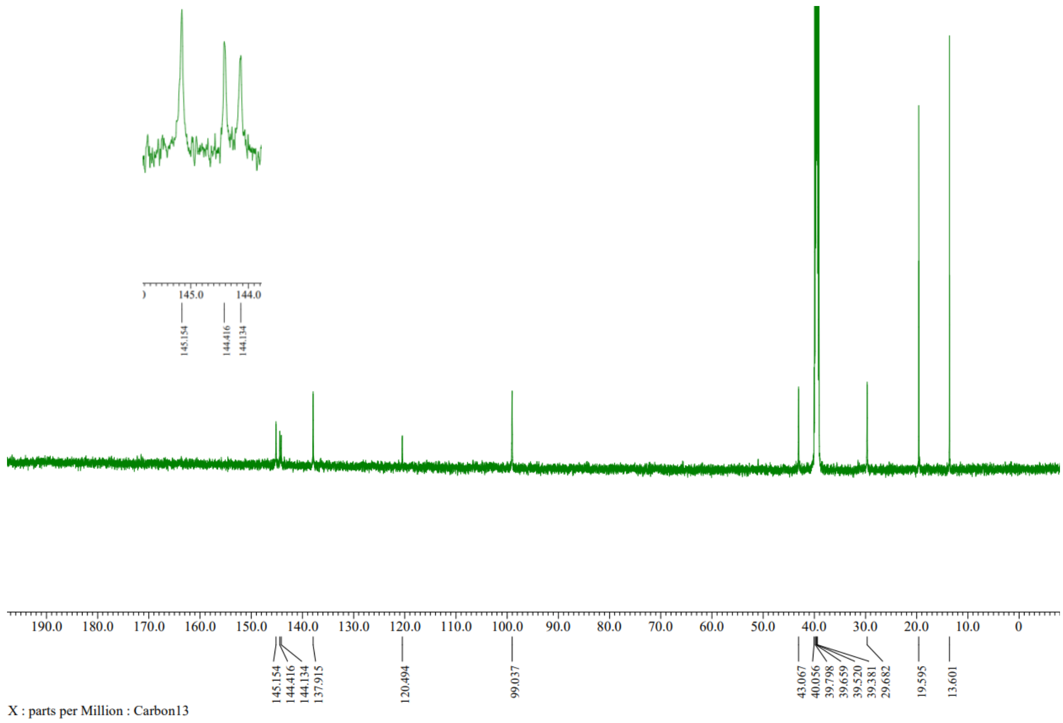

NMR data for **3B**

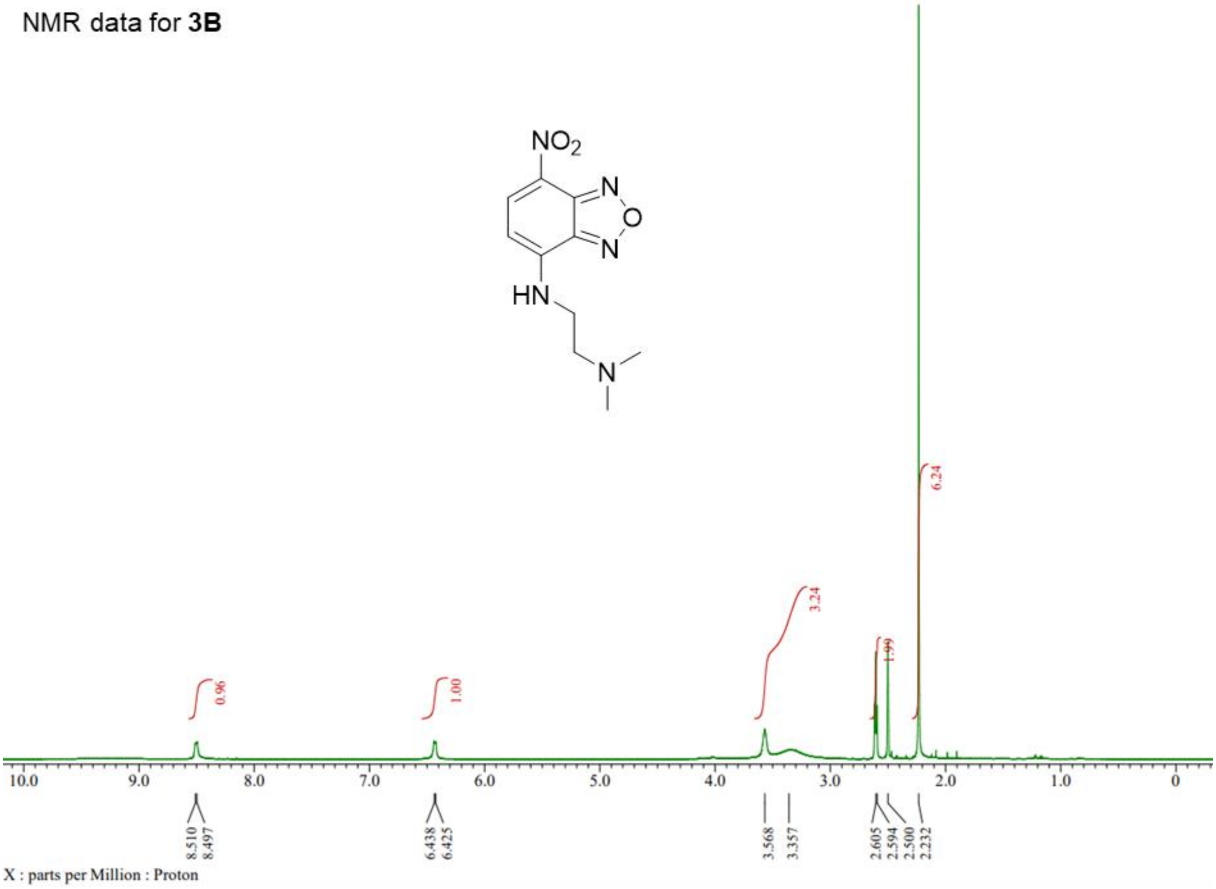

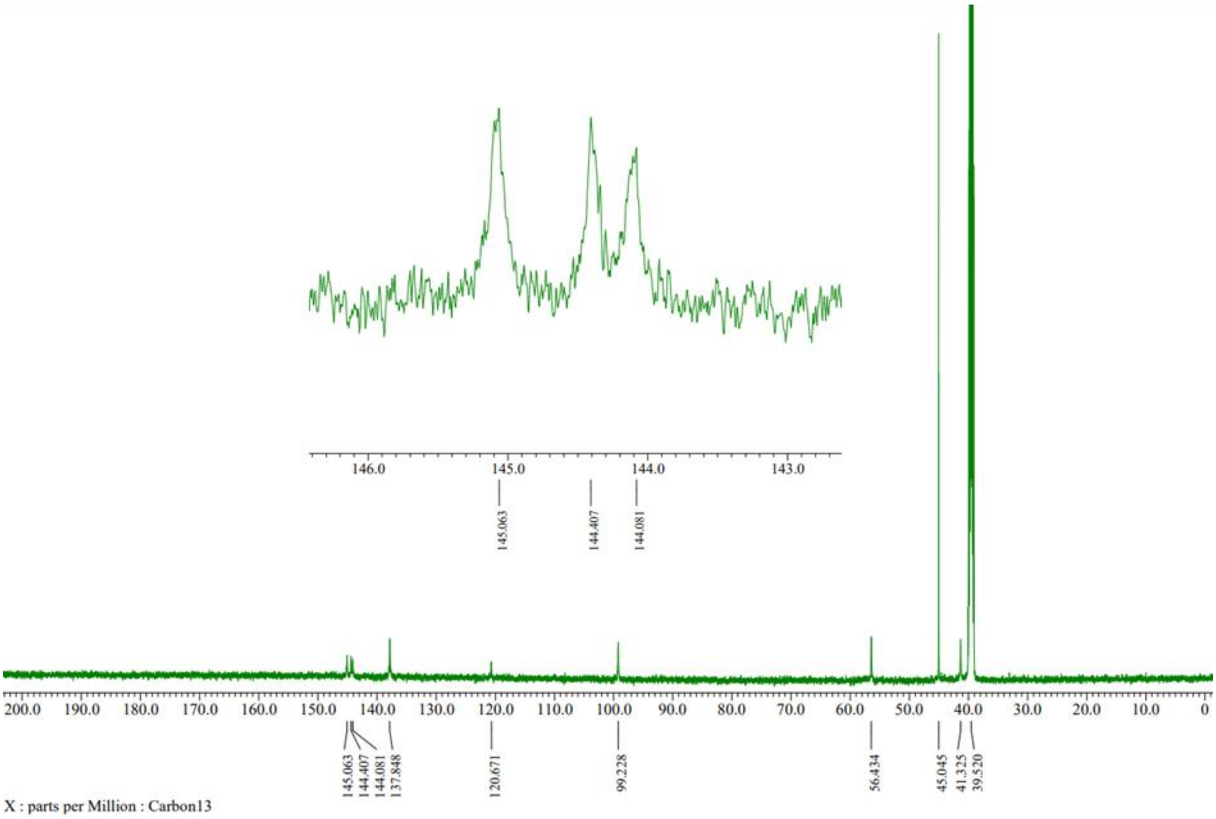

NMR data for **3C**

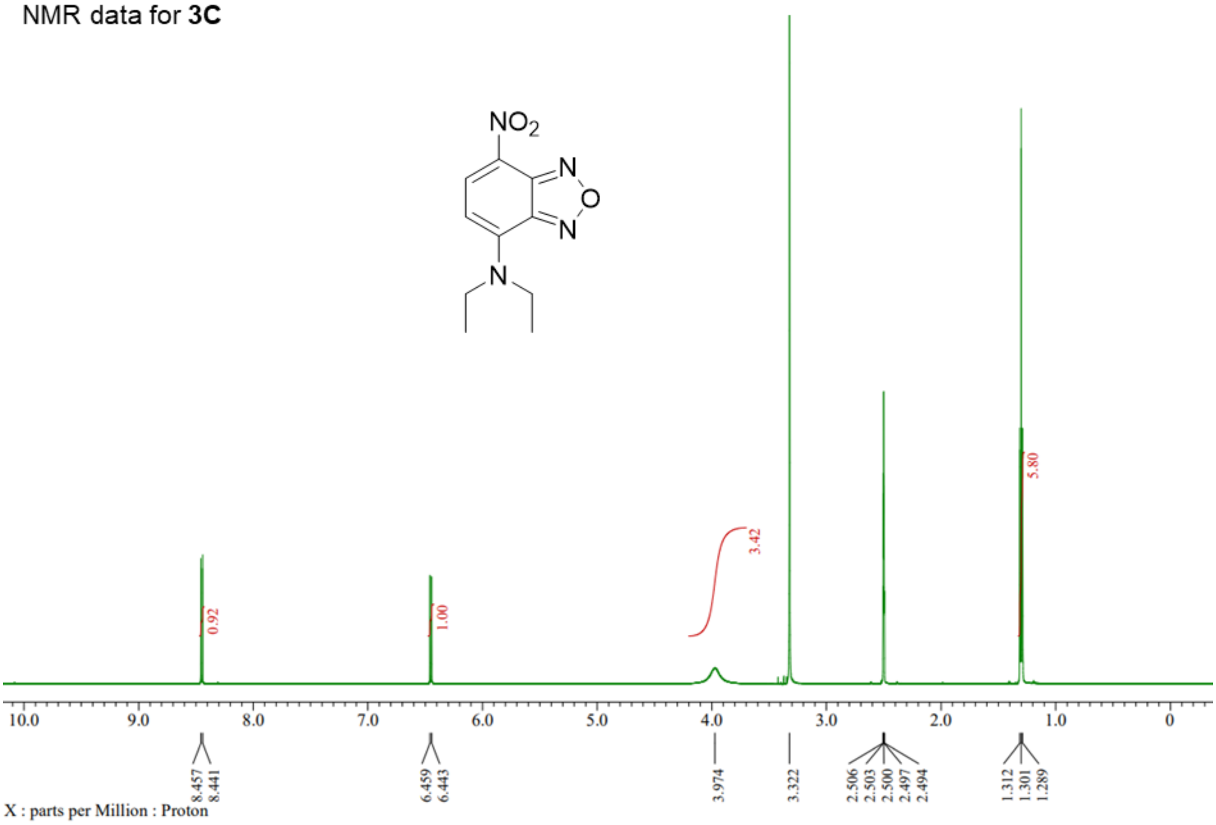

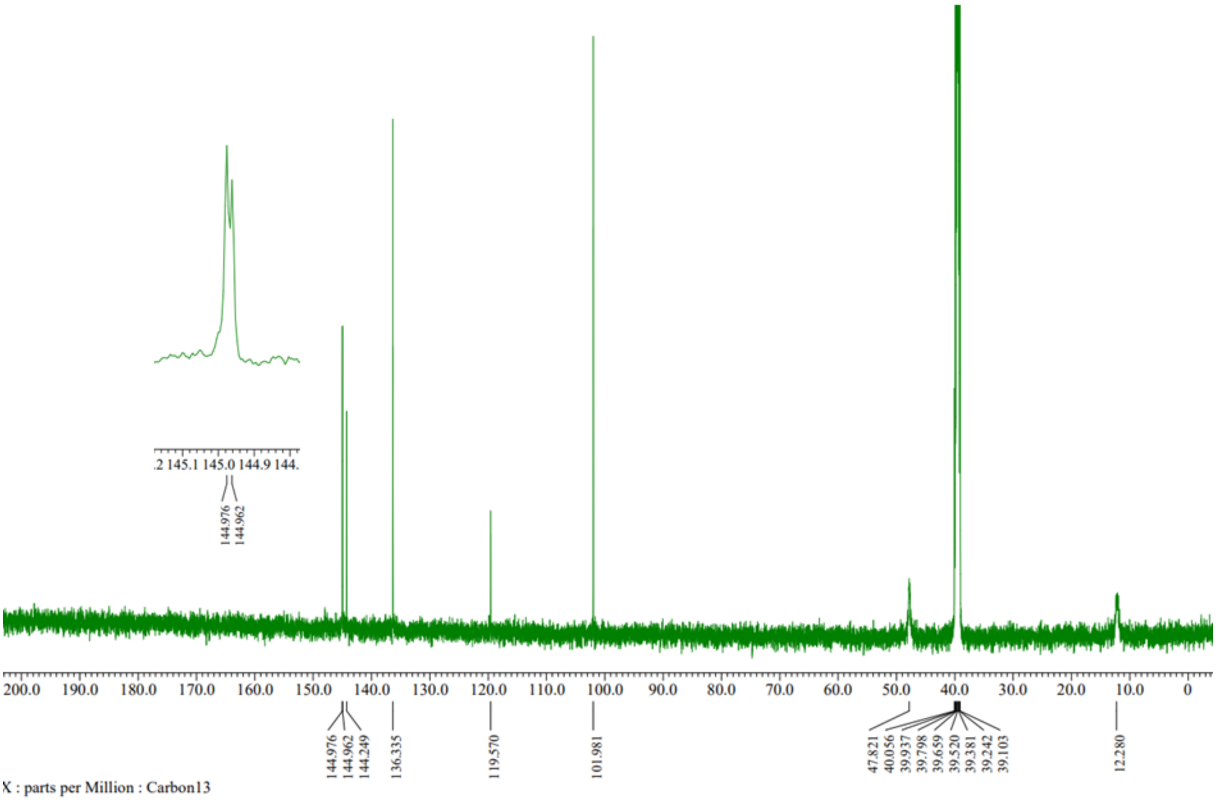

NMR data for **3D**

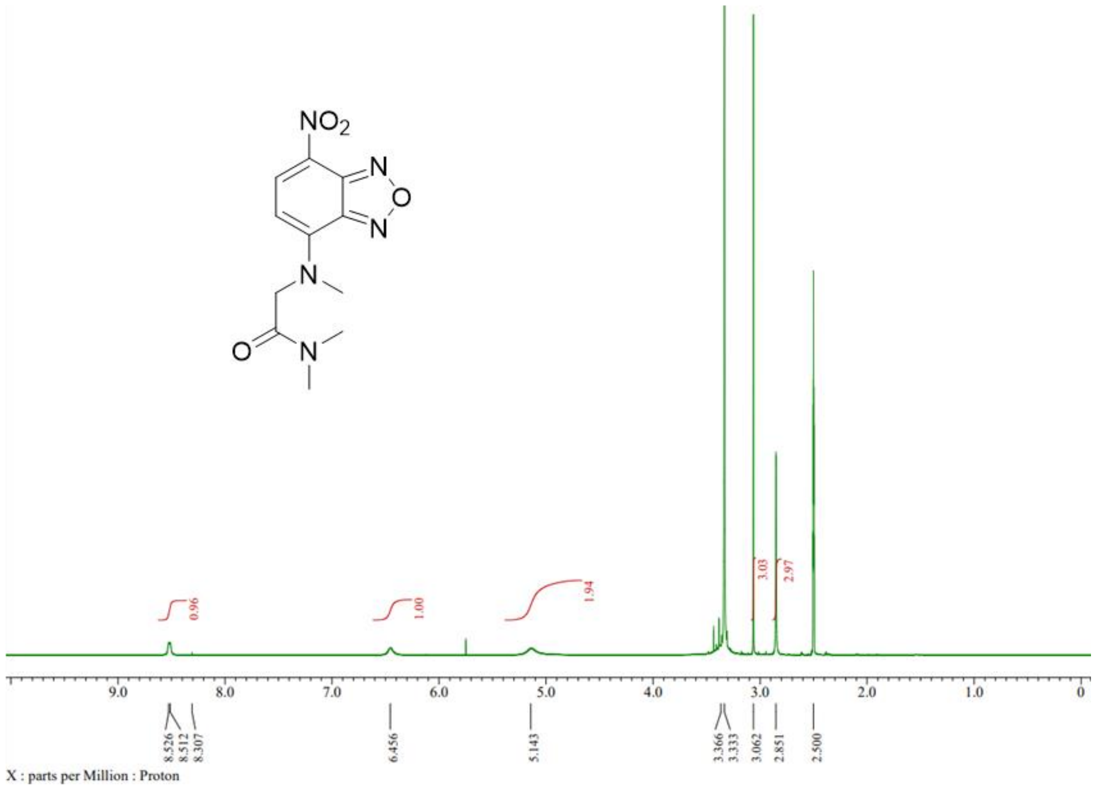

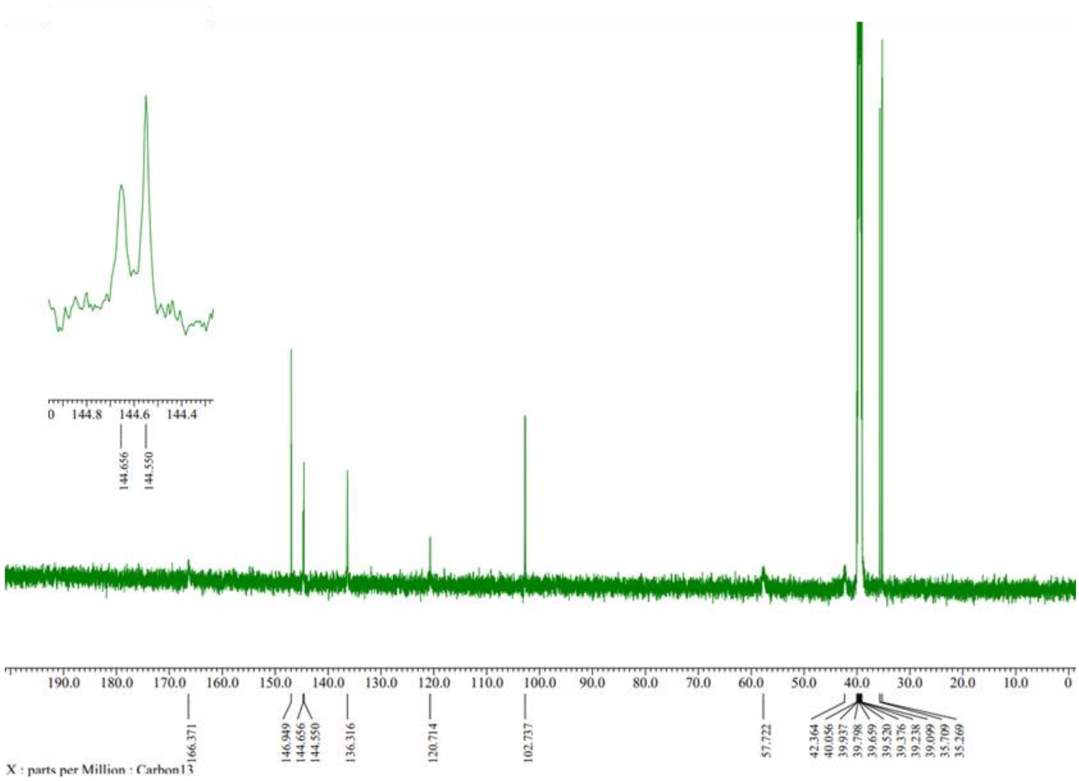

NMR data for **3E**

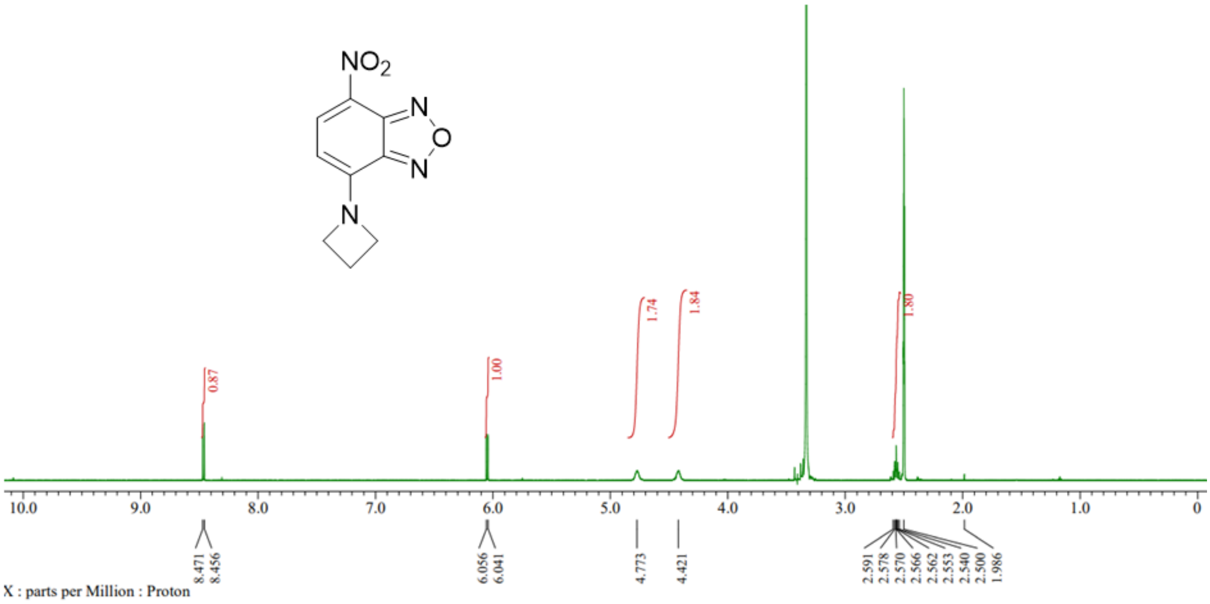

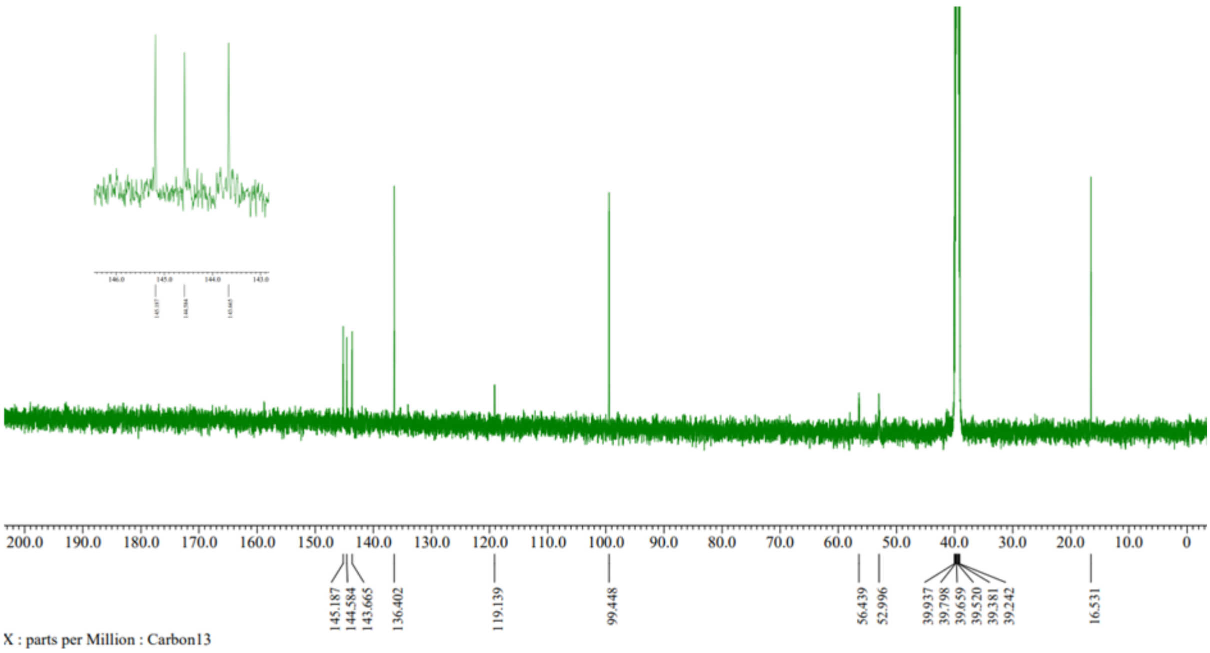

NMR data for **3F**

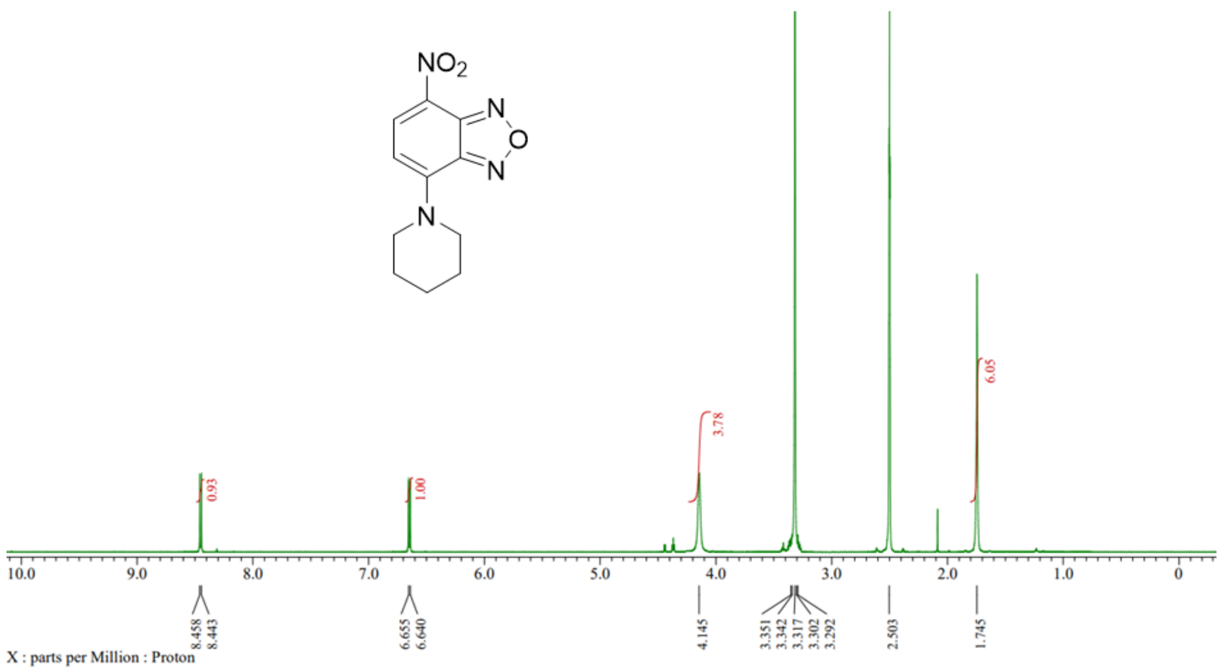

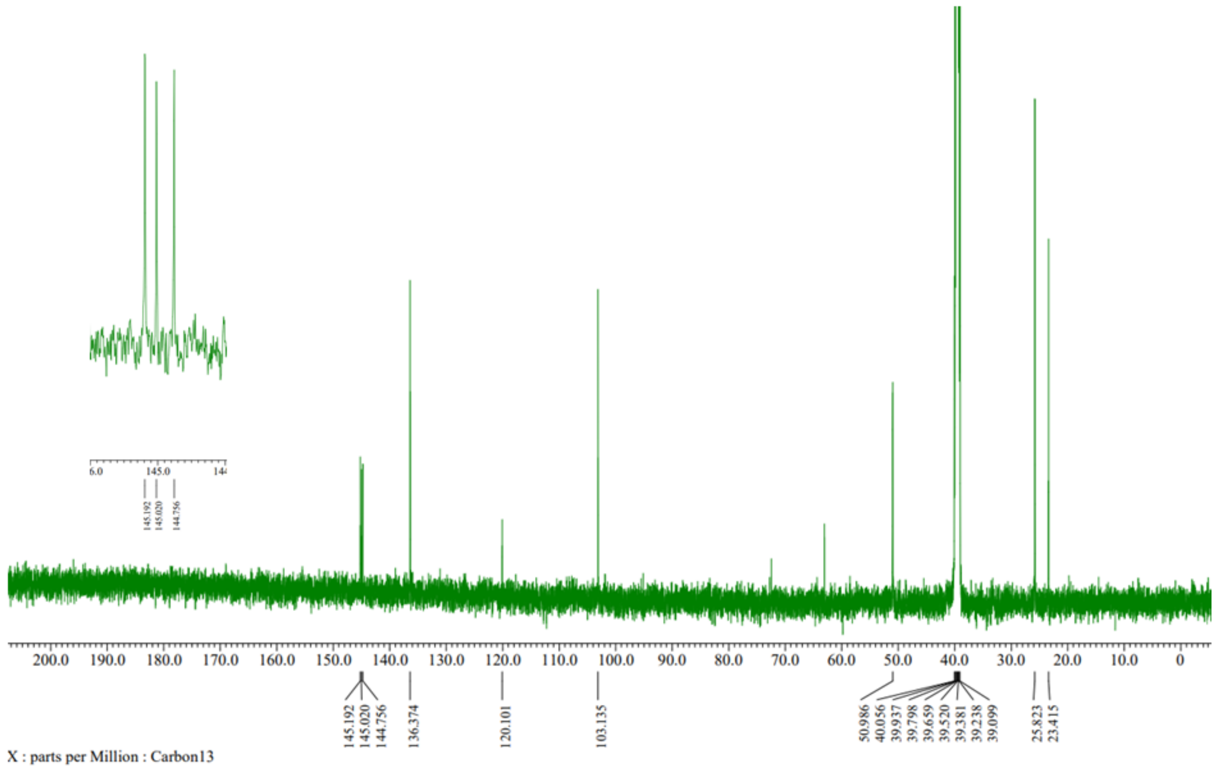

NMR data for **3G**

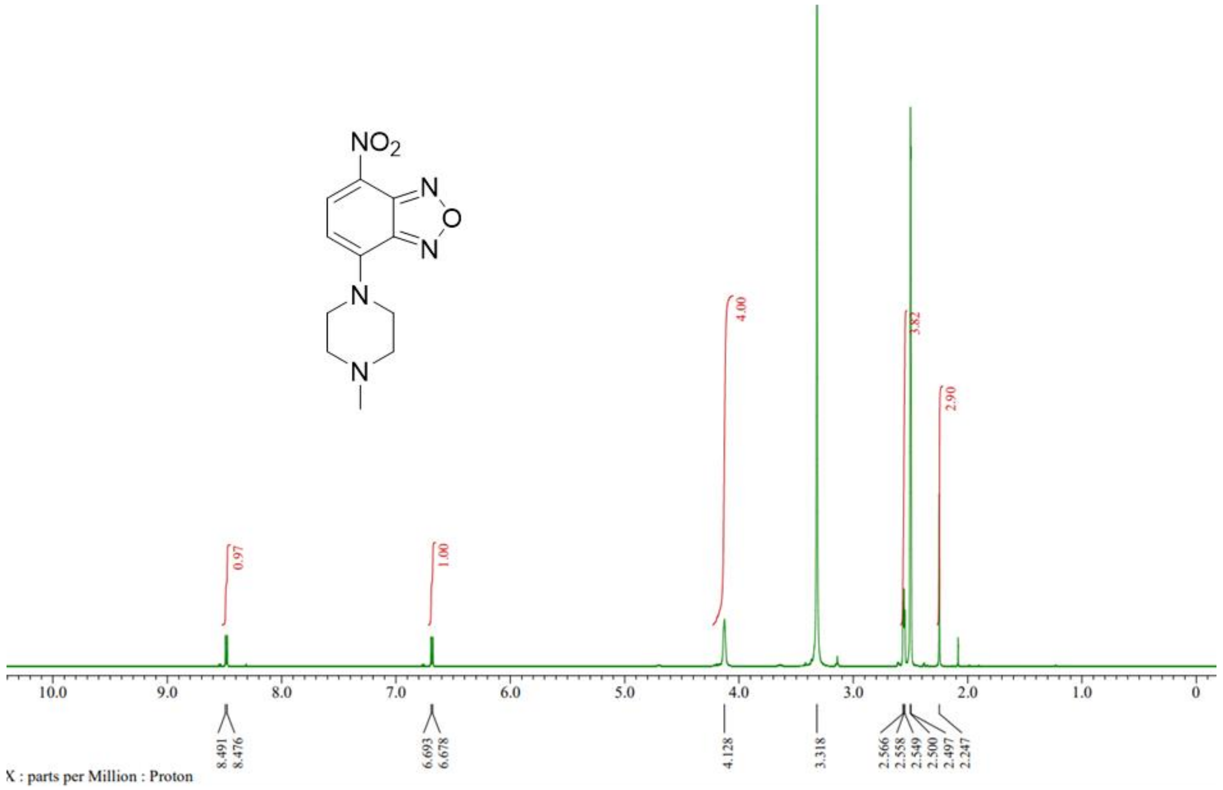

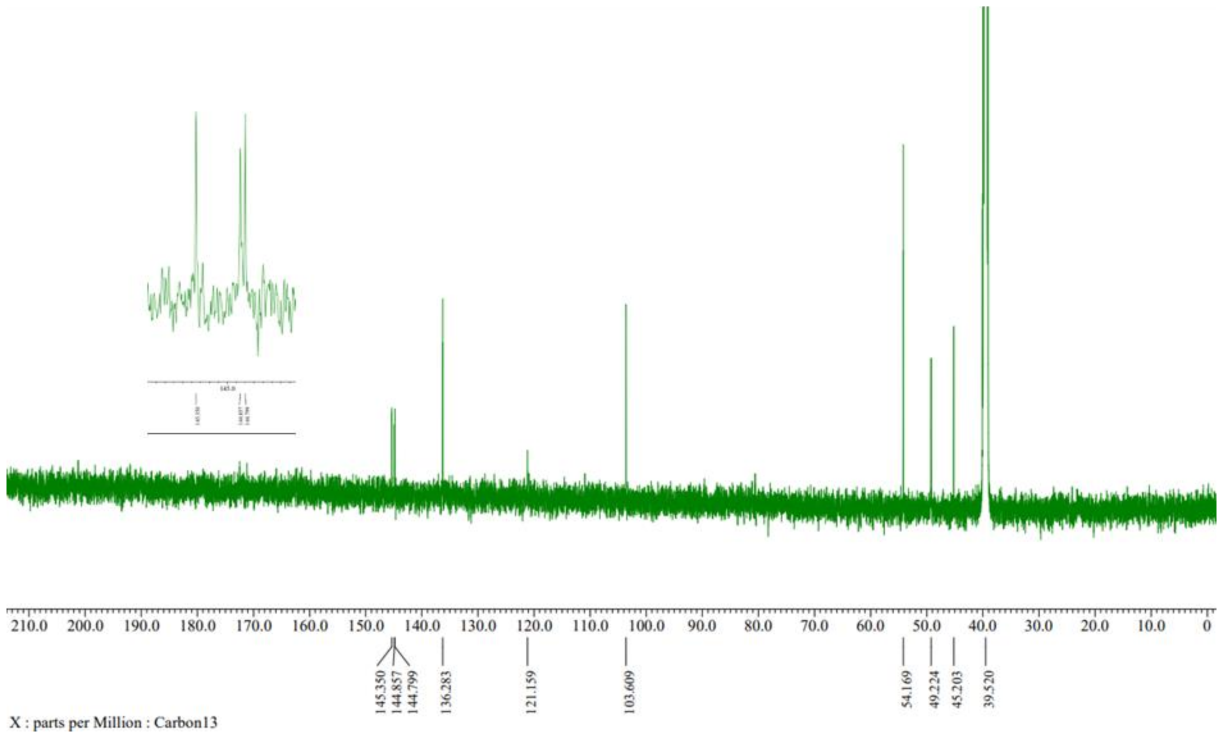

NMR data for **3H**

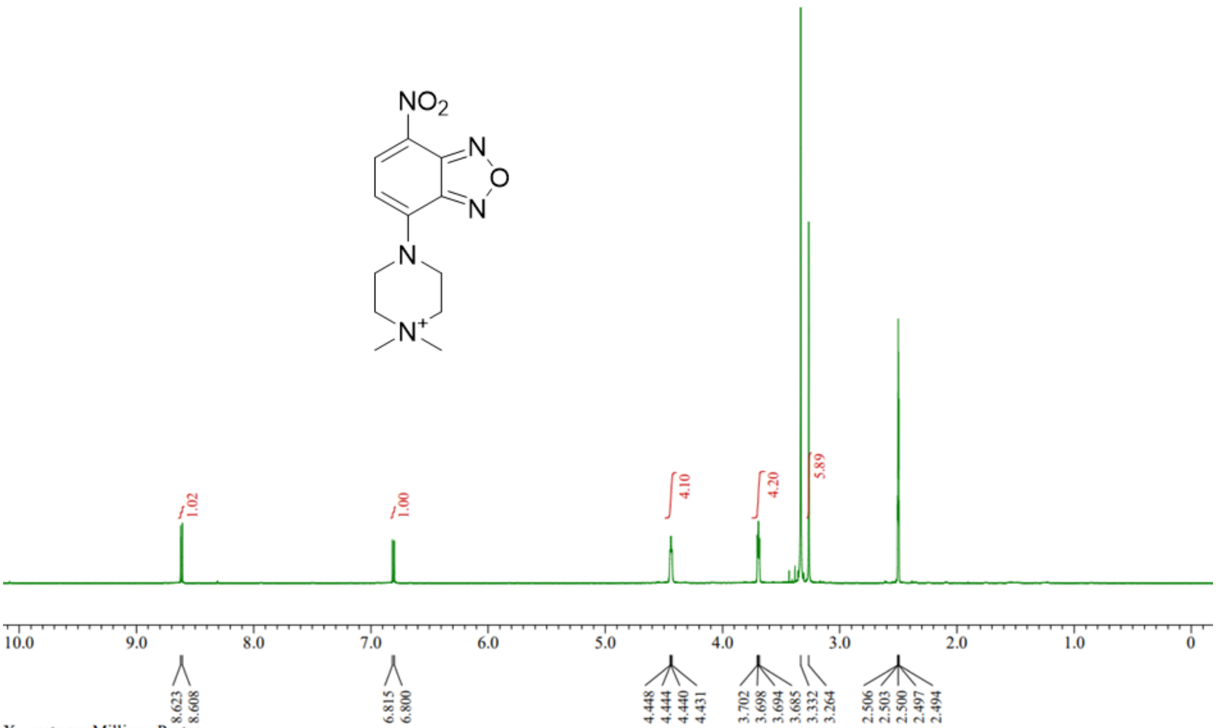

X : parts per Million : Proton

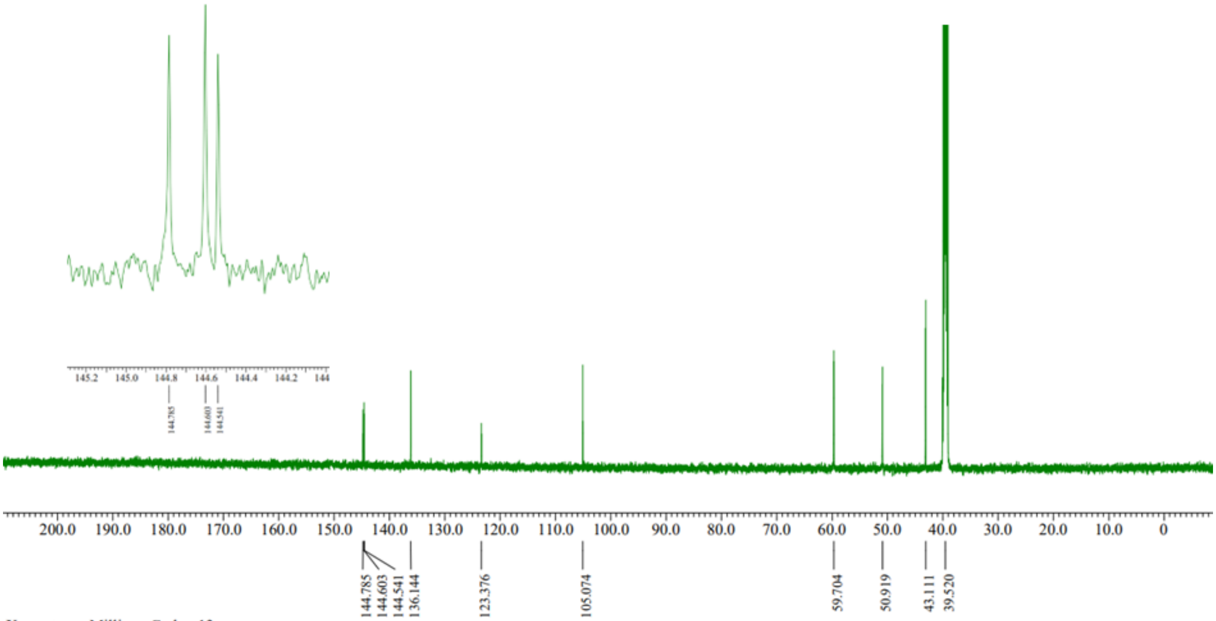

X : parts per Million : Carbon13

HRMS data for 1A

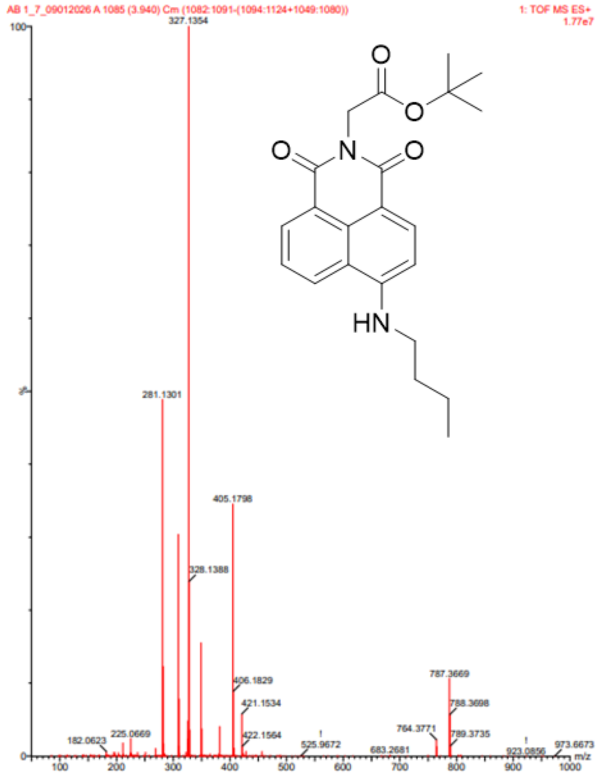

HRMS data for 1B

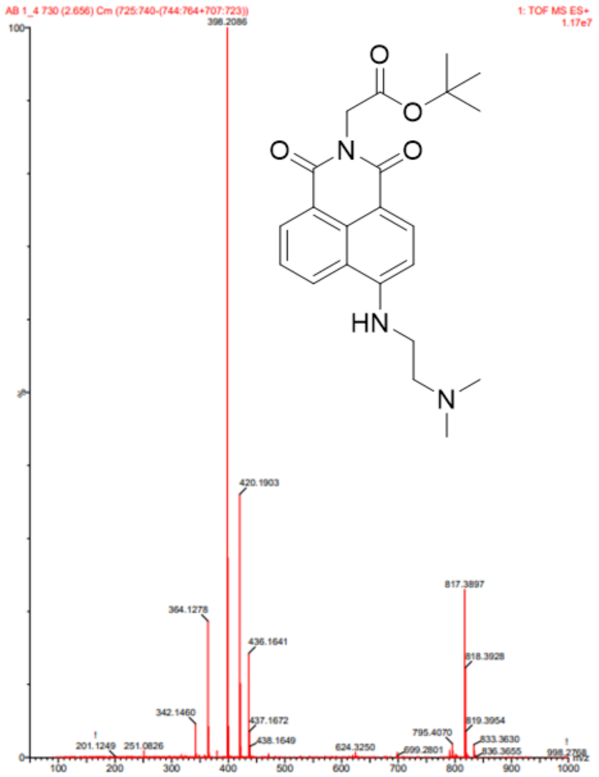

HRMS data for 1C

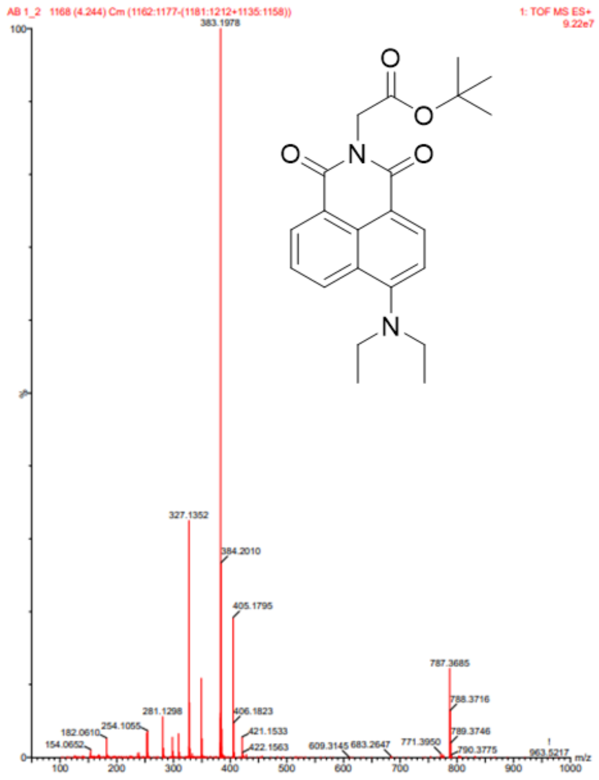

HRMS data for 1D

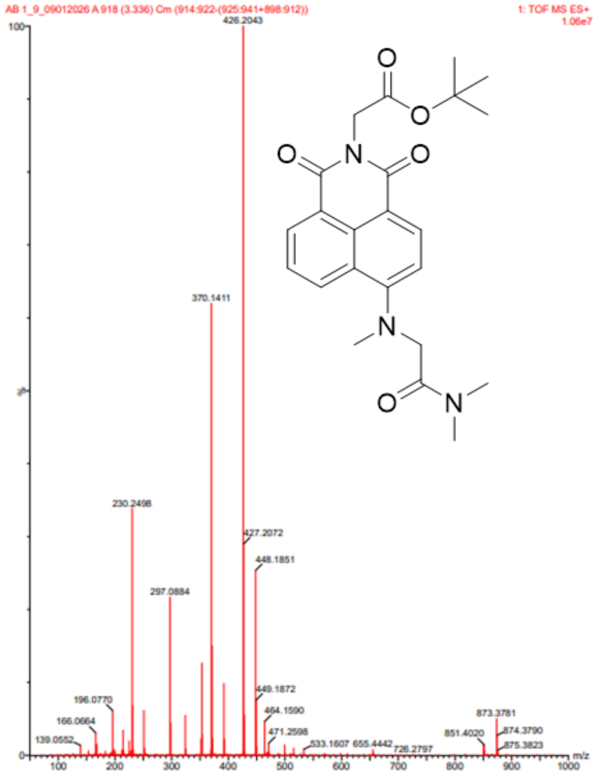

HRMS data for 1E

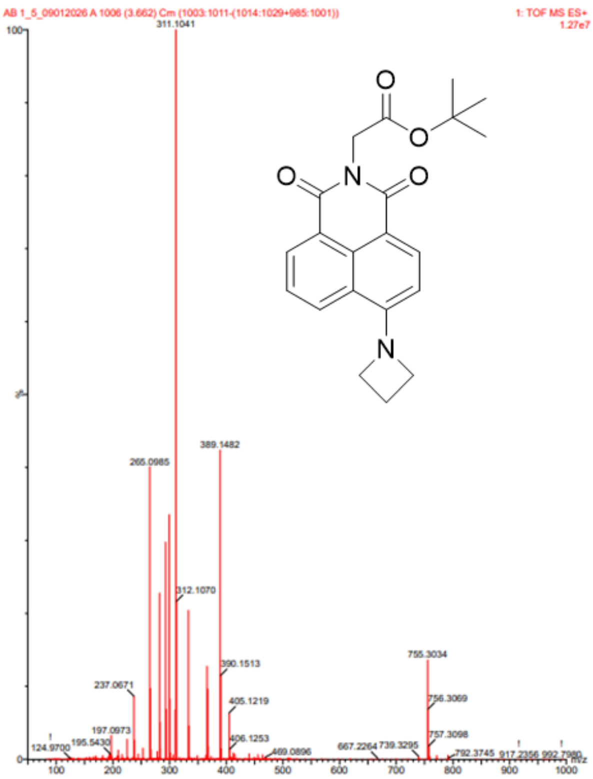

HRMS data for 1F

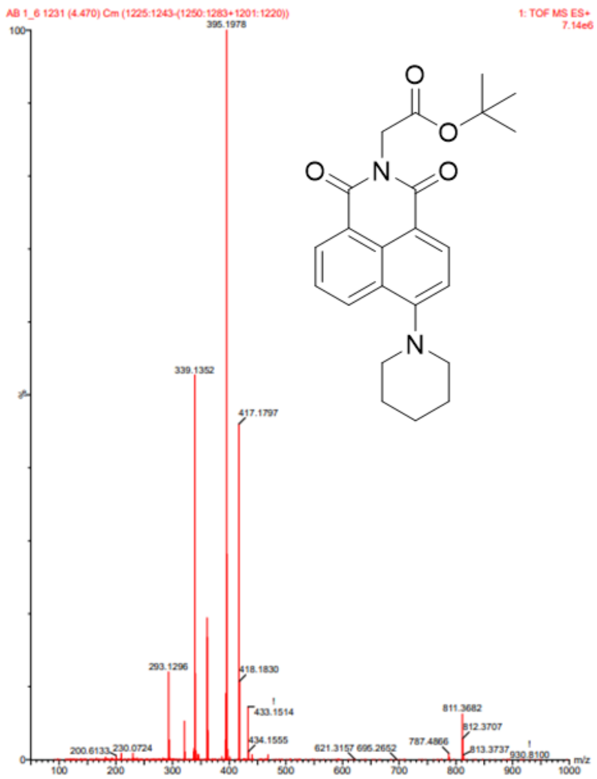

HRMS data for 1G

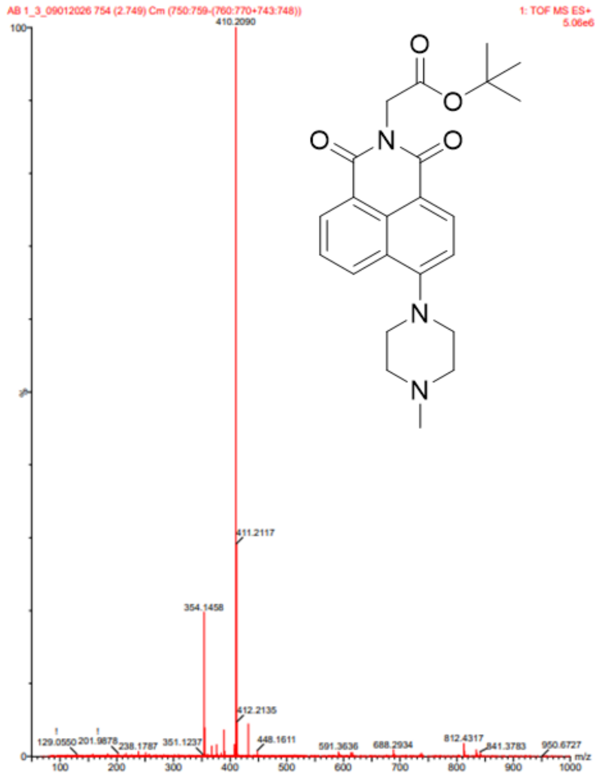

HRMS data for 1H

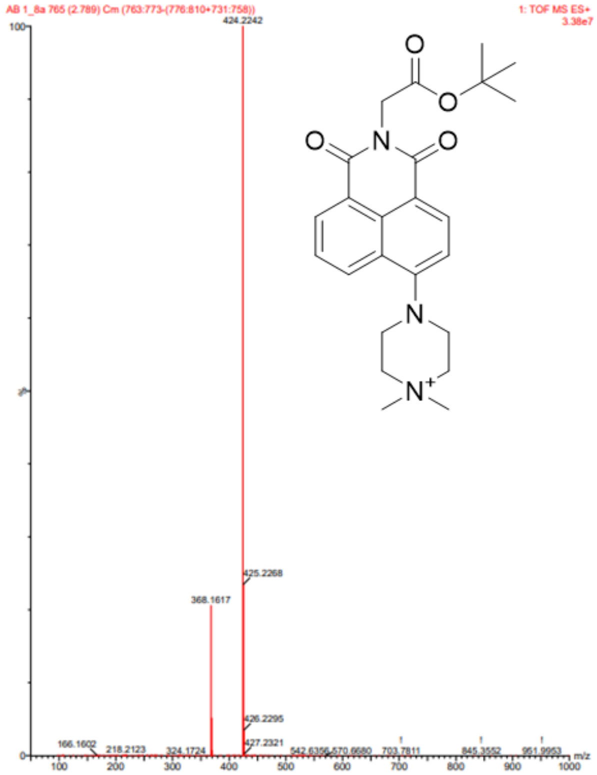

HRMS data for 2A

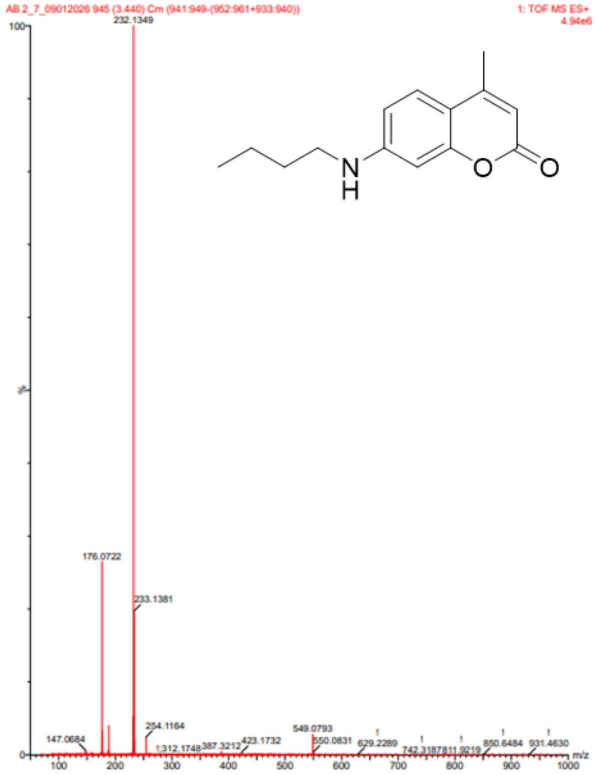

HRMS data for 2B

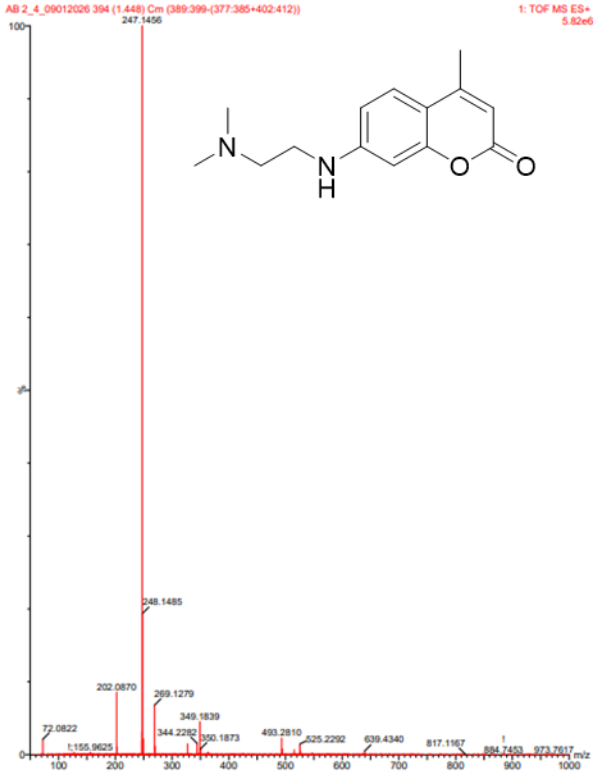

HRMS data for 2C

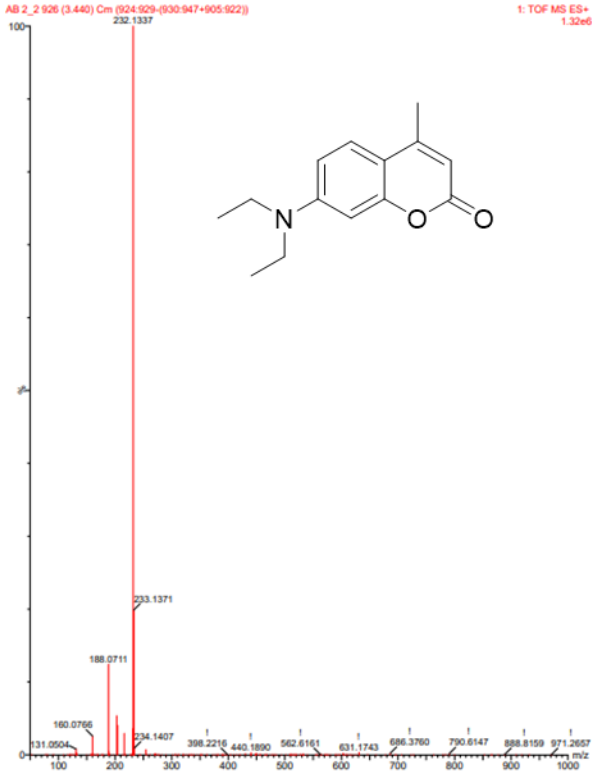

HRMS data for 2D

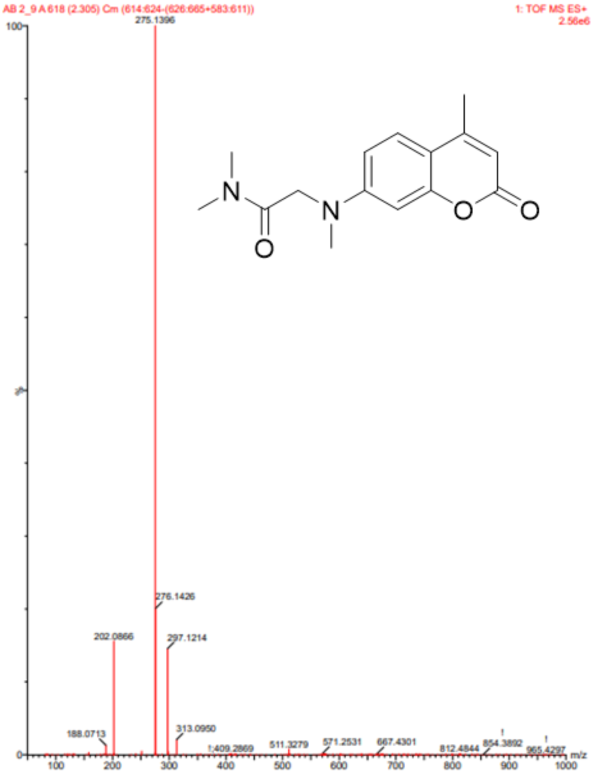

HRMS data for 2E

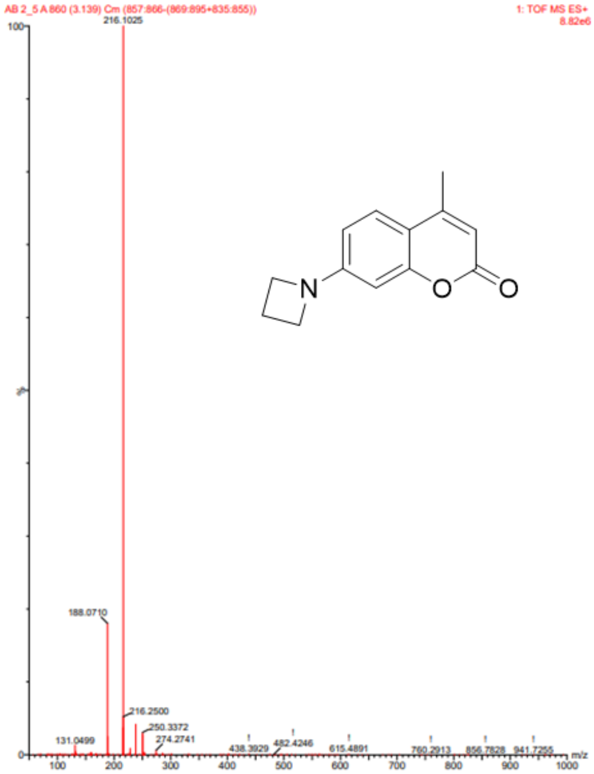

HRMS data for 2F

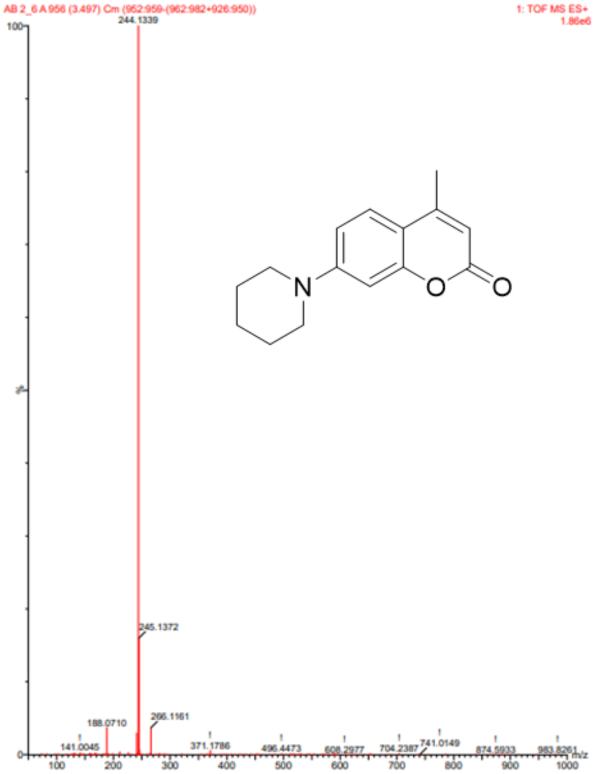

HRMS data for 2G

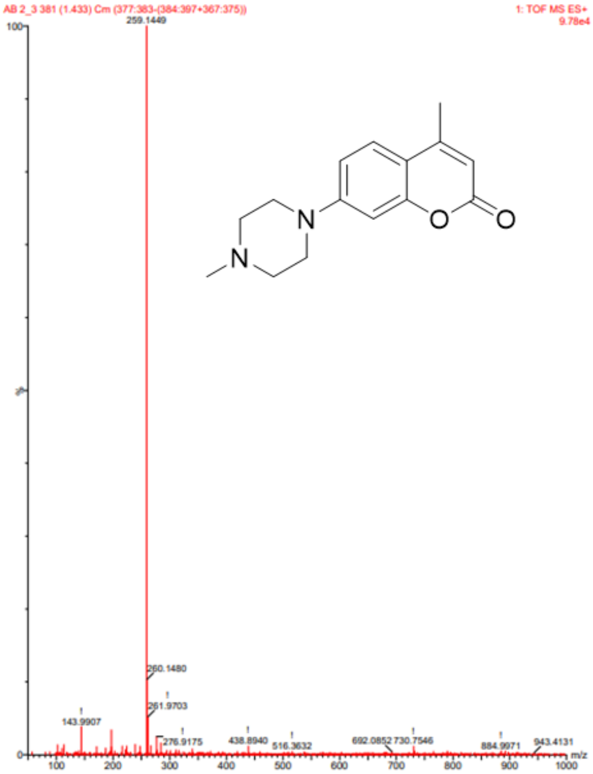

HRMS data for 2H

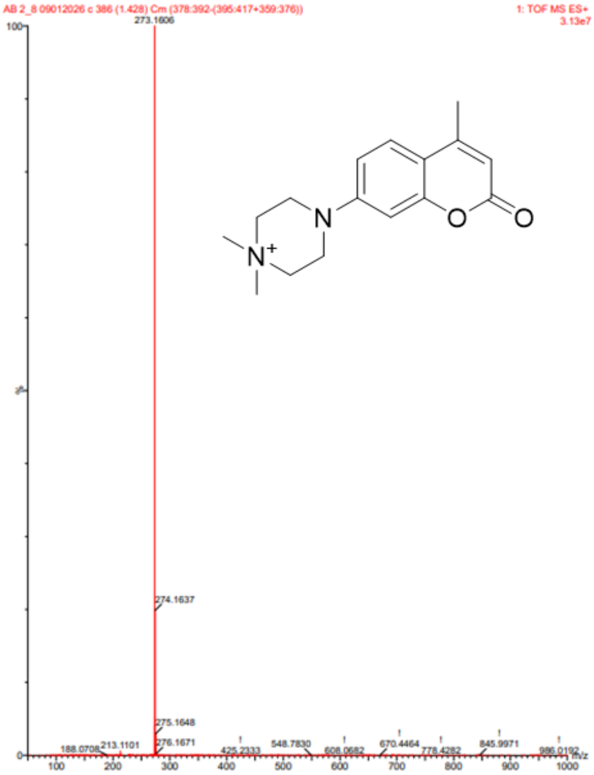

HRMS data for 3A

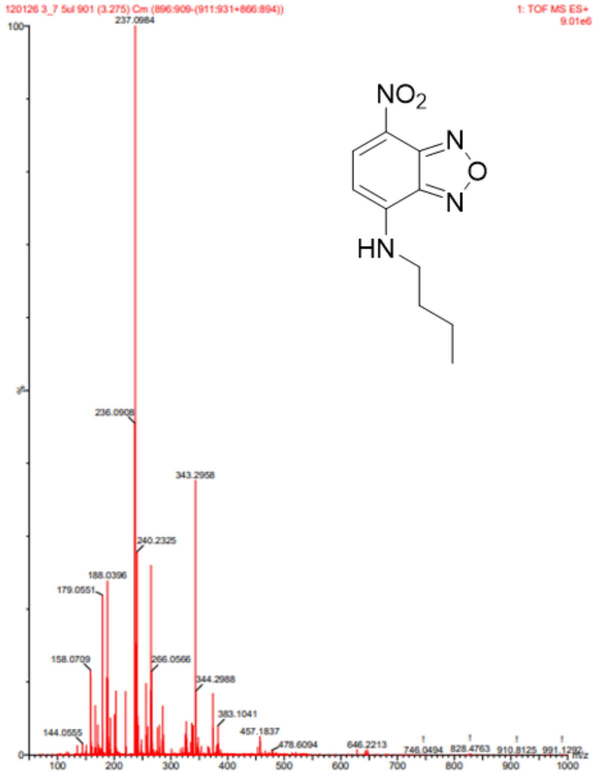

HRMS data for 3B

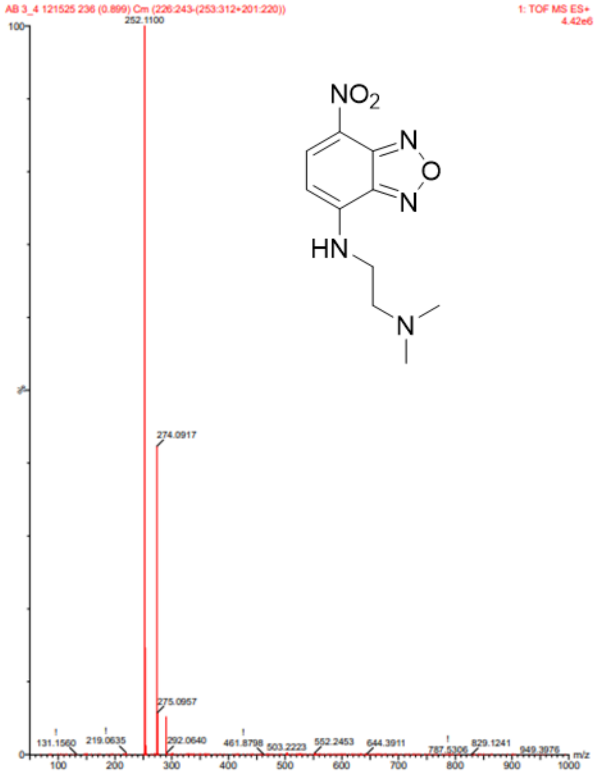

HRMS data for 3C

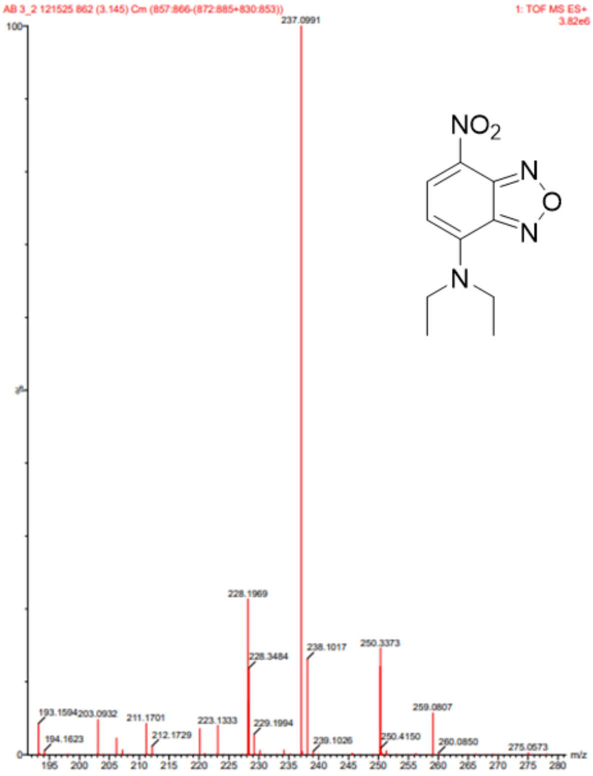

HRMS data for 3D

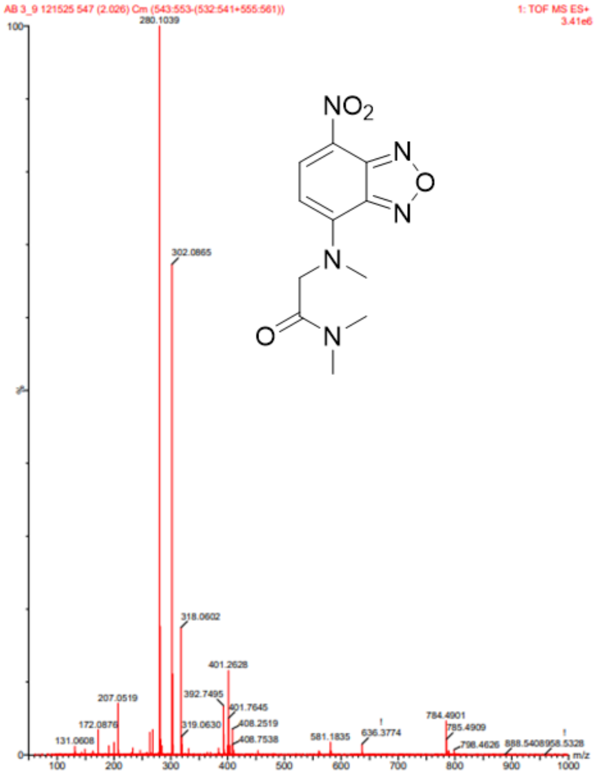

HRMS data for 3E

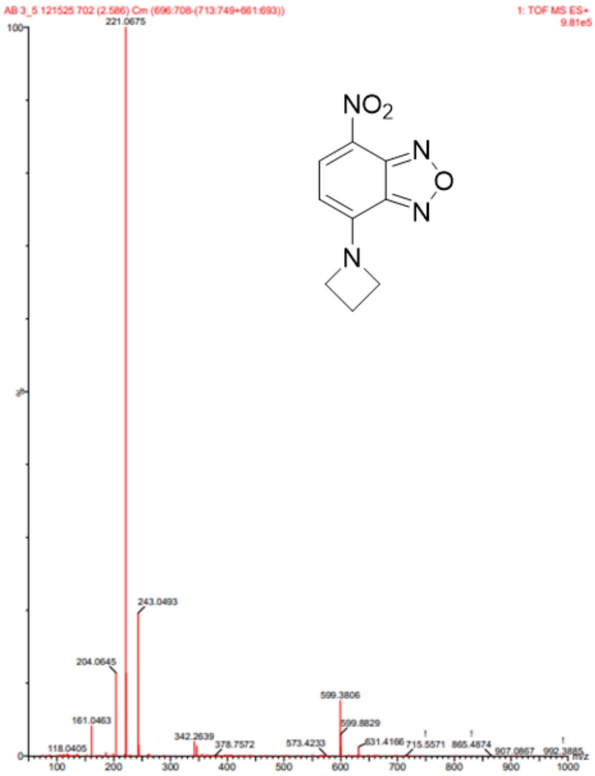

HRMS data for 3F

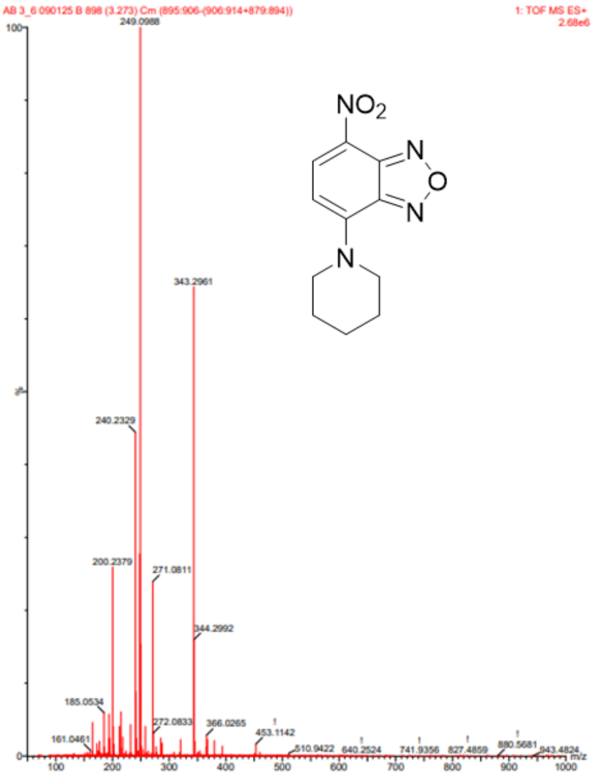

HRMS data for 3G

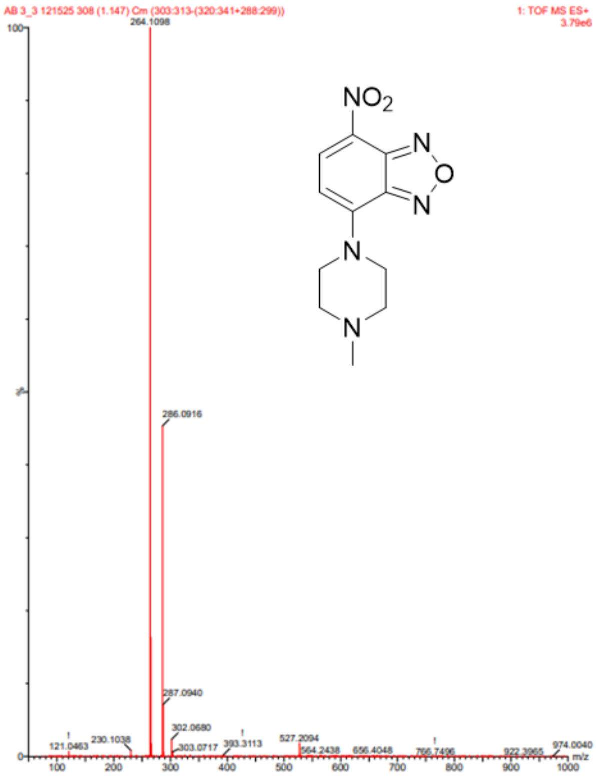

HRMS data for 3H

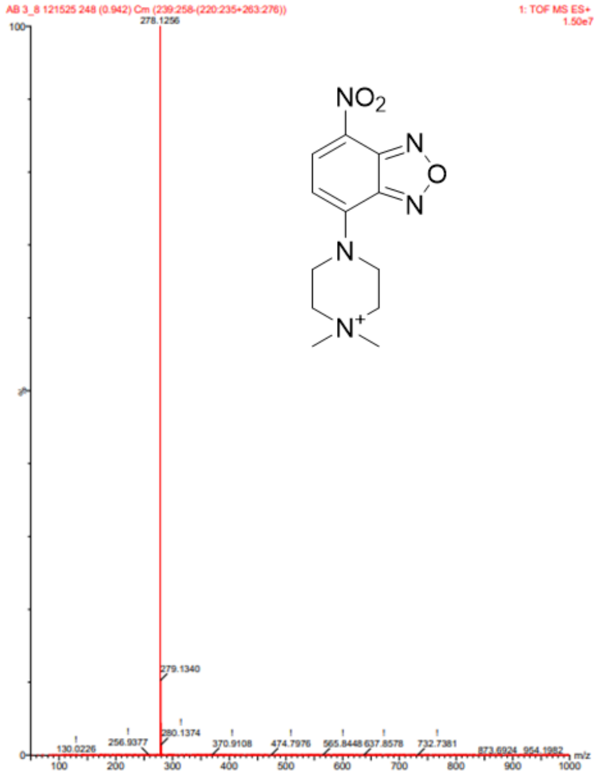

References

[1] J. B. Grimm, B. P. English, J. Chen, J. P. Slaughter, Z. Zhang, A. Revyakin, R. Patel, J. J. Macklin, D. Normanno, R. H. Singer, T. Lionnet, L. D. Lavis, "A general method to improve fluorophores for live-cell and single-molecule microscopy" *Nat. Methods* **2015**, *12*, 244–250.

[2] Z. Ye, W. Yang, C. Wang, Y. Zheng, W. Chi, X. Liu, Z. Huang, X. Li, Y. Xiao, "Quaternary Piperazine-Substituted Rhodamines with Enhanced Brightness for Super-Resolution Imaging" *J. Am. Chem. Soc.* **2019**, *141*, 14491–14495.

[3] J. Kövér, S. Antus, "Facile deoxygenation of hydroxylated flavonoids by palladium-catalysed reduction of its triflate derivatives" *Zeitschrift fur Naturforschung - Section B Journal of Chemical Sciences* **2005**, *60*, 792–796.

[4] K. Suzuki, A. Kobayashi, S. Kaneko, K. Takehira, T. Yoshihara, H. Ishida, Y. Shiina, S. Oishi, S. Tobita, "Reevaluation of absolute luminescence quantum yields of standard solutions using a spectrometer with an integrating sphere and a back-thinned CCD detector" *Physical Chemistry Chemical Physics* **2009**, *11*, 9850–9860.

[5] J. A. Levitt, P. H. Chung, M. K. Kuimova, G. Yahioglu, Y. Wang, J. Qu, K. Suhling, "Fluorescence Anisotropy of Molecular Rotors" *ChemPhysChem* **2011**, *12*, 662–672.
